# Polyphenolic Compounds from *Lespedeza bicolor* Protect Neuronal Cells from Oxidative Stress

**DOI:** 10.3390/antiox11040709

**Published:** 2022-04-03

**Authors:** Darya V. Tarbeeva, Evgeny A. Pislyagin, Ekaterina S. Menchinskaya, Dmitrii V. Berdyshev, Anatoliy I. Kalinovskiy, Valeria P. Grigorchuk, Natalia P. Mishchenko, Dmitry L. Aminin, Sergey A. Fedoreyev

**Affiliations:** 1 G.B. Elyakov Pacific Institute of Bioorganic Chemistry, Far Eastern Branch, Russian Academy of Science, 690022 Vladivostok, Russia; pislyagin_ea@piboc.dvo.ru (E.A.P.); menchinskaya_es@piboc.dvo.ru (E.S.M.); berdyshev@piboc.dvo.ru (D.V.B.); kaaniw@piboc.dvo.ru (A.I.K.); mischenkonp@mail.ru (N.P.M.); daminin@piboc.dvo.ru (D.L.A.); fedoreev-s@mail.ru (S.A.F.); 2 Federal Scientific Center of the East Asia Terrestrial Biodiversity, Far Eastern Branch, Russian Academy of Sciences, 690022 Vladivostok, Russia; kera1313@mail.ru

**Keywords:** polyphenolic compounds, pterocarpans, coumestans, Parkinson’s disease, oxidative stress, neuronal damage

## Abstract

Pterocarpans and related polyphenolics are known as promising neuroprotective agents. We used models of rotenone-, paraquat-, and 6-hydroxydopamine-induced neurotoxicity to study the neuroprotective activity of polyphenolic compounds from *Lespedeza bicolor* and their effects on mitochondrial membrane potential. We isolated 11 polyphenolic compounds: a novel coumestan lespebicoumestan A (**10**) and a novel stilbenoid 5’-isoprenylbicoloketon (**11**) as well as three previously known pterocarpans, two pterocarpens, one coumestan, one stilbenoid, and a dimeric flavonoid. Pterocarpans **3** and **6**, stilbenoid **5**, and dimeric flavonoid **8** significantly increased the percentage of living cells after treatment with paraquat (PQ), but only pterocarpan **6** slightly decreased the ROS level in PQ-treated cells. Pterocarpan **3** and stilbenoid **5** were shown to effectively increase mitochondrial membrane potential in PQ-treated cells. We showed that pterocarpans **2** and **3**, containing a 3’-methyl-3’-isohexenylpyran ring; pterocarpens **4** and **9**, with a double bond between C-6a and C-11a; and coumestan **10** significantly increased the percentage of living cells by decreasing ROS levels in 6-OHDA-treated cells, which is in accordance with their rather high activity in DPPH^•^ and FRAP tests. Compounds **9** and **10** effectively increased the percentage of living cells after treatment with rotenone but did not significantly decrease ROS levels.

## 1. Introduction

Parkinson’s disease (PD) is a common neurodegenerative disease of older age [1]. The pathogenesis of PD includes the death of neurons from oxidative damage as a result of an increase in the production of intracellular reactive oxygen species (ROS), which causes damage to lipids, proteins, and DNA. A combination of oxidative stress, mitochondrial dysfunction, protein misprocessing, and genetic factors plays a crucial role in the pathogenesis of age-related neurodegeneration [1,2]. The etiology of PD can be associated not only with aging but also with adverse environmental influences and neurotoxins. 6-hydroxydopamine (6-OHDA) and 1-methyl-4-phenyl-1,2,3,6-tetrahydropyridine (MPTP) were shown to cause PD symptoms [3]. Several pesticides and herbicides (rotenone, paraquat (PQ), maneb (MB), and mancozeb (MZ)) are also neurotoxins and cause pathologies similar to PD [3]. The neurotoxic properties of these compounds are primarily due to their ability to generate toxic free radicals and reactive oxygen species in cells. For example, PQ affects the redox cycle and activity of the enzyme nitric oxide synthase in neuronal cells, which leads to increased production of ROS and increased levels of α-synuclein and tau protein, α-tubulin hyperacetylation, inhibition of proteasomes, and dysfunction of axonal autophagy. All these factors eventually cause PD symptoms [3]. Being a structural analogue of dopamine, 6-OHDA selectively penetrates dopaminergic and noradrenergic neurons, accumulates in the cytosol, and changes into dihydrophenylacetic acid or oxidizes to form hydrogen peroxide and *para*-quinone, which leads to ROS formation and oxidative stress in neurons, followed by cell death [3]. Rotenone easily crosses all cellular membranes and inhibits the transition of electrons from the iron-sulfur centers in complex I to ubiquinone. By doing so, rotenone inhibits reduced nicotinamide adenine dinucleotide (NADH)-ubiquinone reductase activity and impairs oxidative phosphorylation [3]. Thus, these neurotoxins can be used to cause neuronal cell damage in models of PD [3,4,5]. Being natural antioxidants, flavonoids and isoflavonoids are promising for the study of their neuroprotective properties [6]. 

Among isoflavonoids, pterocarpans are the second largest group. They contain a tetracyclic ring system derived from the flavonoid skeleton and have two chiral centers at carbon atoms C-6a and C-11a [7]. Pterocarpans have been reported to exhibit numerous bioactivities, including antioxidant, antineuroinflammatory, antimalarial, anticancer, and antimicrobial effects [7,8]. Pterocarpans have been reported to possess remarkable neuroprotective properties. For example, maackiain, widespread in plants of the Fabaceae family, was shown to significantly reduce dopaminergic neuron damage in 6-OHDA-exposed worms and to diminish the accumulation of α-synuclein [9].

The neuroprotective activity of pterocarpans may be due to their ability to inhibit two isoforms of monoamine oxidase: MAO-A and MAO-B. Maackiain and medicarpin selectively inhibited MAO-B, with one of the lowest IC_50_ values reported so far [10,11], but they did not effectively inhibit MAO-A. Meanwhile, (–)-4-hydroxy-3-methoxy-8,9-methylenedioxypterocarpan was found to effectively inhibit both the MAO-A and MAO-B isoforms [10]. Maackiain also exhibited inhibitory effects on LPS-induced nitric oxide production in RAW 264.7 macrophages [12] and inhibited 5-lipoxygenase [13], thus preventing damage to dopaminergic neurons [12,13,14]. 

Naturally occurring coumestans contain an additional carbonyl group compared to pterocarpans. These compounds also showed protective effects against neuronal damage, hypoxia–ischemia-induced cognitive impairment, and neurodegenerative disorders, including PD. Coumestrol attenuated the neurotoxicity induced by LPS and amyloid-beta peptide (Aβ) and inhibited the production of inflammatory cytokines TNF-α, IL-1, and IL-6 [15]. This compound not only provided effective neuroprotection in a cerebral global-ischemia model [16] but also modulated mitochondrial activity and decreased oxidative stress in rats [17,18].

The Far Eastern medicinal plant *Lespedeza bicolor* and other Lespedeza species produce unusual biologically active prenylated pterocarpans and coumestans, possessing strong antioxidant and neuroprotective activity [19,20,21,22,23,24,25,26,27,28]. Pterocarpan 1-methoxylespeflorin G_11_ from *L. bicolor* has recently been reported to exhibit neuroprotective effects against glutamate-induced neurotoxicity in neuronal HT22 cells. This neuroprotective activity was shown to be due to inhibition of oxidative stress and apoptosis [25]. We should note that arylbenzofuran and coumestan compounds did not protect HT22 cells from glutamate-induced cell death, whereas the pterocarpan-type compounds exhibited a significant neuroprotective effect against glutamate neurotoxicity [25].

Here, we studied the neuroprotective effect of polyphenolic compounds from *L. bicolor* growing in the Russian Far East using models of PQ-, 6-OHDA-, and rotenone-induced neurotoxicity; we also studied their effects on mitochondrial potential.

## 2. Materials and Methods

### 2.1. Plant Material

*L. bicolor* was collected by academician Gorovoy P.G. in Khasansky District (Andreevka village) of the Primorye Territory (The Russian Federation) in August 2016. A voucher specimen (No. 103608) was deposited into the herbarium of the Laboratory of Chemotaxonomy (G.B. Elyakov Pacific Institute of Bioorganic Chemistry, FEB RAS, Vladivostok, Russia).

### 2.2. Extraction and Isolation

Air-dried root bark of *L. bicolor* (330 g) was extracted for 3 h at 60 °C twice under reflux with a CHCl_3_–EtOH mixture (3:1, *v/v*). The dried extract (3.4 g) was subjected to a polyamide (100 g, 50–160 µm, Sigma-Aldrich, St. Louis, MI, USA) column (15 × 5 cm) and eluted with a hexane–CHCl_3_ solution system with gradually increasing CHCl_3_ percentage (hexane/CHCl_3_, 1:0, 100:1, 50:1, 40:1, *v*/*v*) to give fractions 1–16 and then with a CHCl_3_–EtOH solution system with gradually increasing EtOH amounts (CHCl_3_/EtOH, 1:0, 100:1, 50:1, 40:1, *v*/*v*) to give fractions 17–23.

We subsequently purified the fractions containing polyphenolic compounds according to HPLC data. Fraction 19 (CHCl_3_–EtOH (50:1), 408 mg) was chromatographed twice over a silica gel (40–63 µm, Sigma-Aldrich, St. Louis, MI, USA) column (11 × 1.4 cm). The column was eluted with a benzene–ethylacetate solution system with gradually increasing ethylacetate percentage (benzene/EtOAc, 1:0, 200:1, 100:1, 50:1, 40:1, *v*/*v*) to yield compound **4** (14.4 mg). Fraction 20 (215 mg), washed out with CHCl_3_–EtOH (40:1), was also chromatographed over a silica gel column using the same solution system to obtain compounds **1** (12.1 mg), **4** (5.5 mg), and **10** (3.7 mg). Fraction 22 (427 mg), eluted with CHCl_3_−EtOH (20:1), was chromatographed on a silica gel (40–63 µm) column (11×1.4 cm) using the same solution system to obtain compounds **5** (6.9 mg), **7** (2.9 mg), **8** (7.9 mg), and **11** (6.3 mg).

Fraction 15 (CHCl_3_, 193 mg) was subsequently subjected to a silica gel (40–63 µm) column (11 × 1.4 cm) and eluted with a benzene–ethylacetate solution system with gradually increasing ethylacetate percentage (benzene/EtOAc, 1:0, 200:1, 100:1, 50:1, 40:1, *v*/*v*) twice to give compounds **6** (7.2 mg) and **9** (3.6 mg). Fraction 16 (89 mg), washed out with CHCl_3_, was also further chromatographed over a silica gel (40–63 µm) column twice using the same eluent system to afford compounds **2** (7.2 mg) and **3** (9.0 mg). 

A semi-preparative HPLC technique was used as a final step for purification of the isolated compounds.

*Lespebicoumestan A* (**10**): White, amorphous powder; UV (MeOH) λ_max_ 211, 257, 358, 371 nm; ECD (3.99 × 10^−4^ M, CH_3_CN) λ_max_ (Δε) 196 (+1.33), 216 (−0.77), 219 (−0.76), 280 (+0.17); ^1^H and ^13^C NMR data, see Table 1; HR-ESI-MS *m/z* 417.1337 [M-H]^−^ (calculated for [C_25_H_21_O_6_]^−^ 417.1344), *m/z* 419.1479 [M+H]^+^ (calculated for [C_25_H_23_O_6_]^+^ 419.1489).

*5′-isoprenylbicoloketone A* (**11**): Yellow, amorphous powder; UV (MeOH) λ_max_ 220, 293, 345 nm; ^1^H and ^13^C NMR data, see Table 2; HR-ESI-MS *m/z* 493.2217 [M-H]^−^ (calculated for [C_29_H_33_O_7_]^−^ 493.2232), *m/z* 495.2401 [M+H]^+^ (calculated for [C_29_H_35_O_7_]^+^ 495.2377).

### 2.3. General Experimental Procedures

We recorded the UV spectra on a UV-1601 PC spectrophotometer (Shimadzu, Kyoto, Japan). The CD spectra were recorded using a Chirascan-Plus Quick Start CD Spectrometer (Applied Photophysics Limited, Leatherhead, UK) (acetonitrile, 20 °C). The ^1^H, ^13^C, and two-dimensional NMR spectra (HSQC, HMBC, COSY, ROESY) were recorded in CDCl_3_ on a Bruker AVANCE III DRX-700 NMR spectrometer (Bruker, Karlsruhe, Germany). The chemical shift values (*δ*) and the coupling constants (*J*) are given in parts per million and Hz, respectively. 

### 2.4. Analytical and Semi-Preparative HPLC

We carried out analytical HPLC using an Agilent Technologies 1260 Infinity II HPLC system (Agilent Technologies, Waldbronn, Germany) equipped with a VWD detector (λ = 280 nm). The extracts and fractions were analyzed at a flowrate of 0.8 mL/min using an HS-C18 column (3 μm, 4.6, 75 mm, Supelco Analytical, Bellefonte, PA, USA). The column was thermostated at 30 °C. We used a mixture of 1% aqueous acetic acid (A) and acetonitrile containing 1% acetic acid (B) as mobile phase. We programmed the following gradient steps for elution: 0–2 min–5% B, 2–4 min–5–20% B, 5–17 min–20–50% B, 18–23 min–50–90% B, 24–25 min–90–100% B, 16–27 min–100% B, and 28–33 min–100–5% B. The data were analyzed using OpenLab CDC software v2.4 (Agilent Technologies, Waldbronn, Germany).

Semi-reparative HPLC was performed using a Shimadzu HPLC system equipped with an LC-20AT pump and SPD-20A detector (λ = 280 nm) (Shimadzu, Kyoto, Japan). We purified polyphenolic compounds using a silica gel YMC-Pack SIL (Supelco Analytical, Bellefonte, PA, USA) column (5 μm, 10, 250 mm) at a flowrate of 4.5 mL/min. The mobile phase consisted of hexane (97%) and isopropanol (3%). We used Shimadzu LCMS Solution software v5.93 (Shimadzu, Kyoto, Japan) to acquire and process the data.

### 2.5. HR-ESI-MS

We performed HR-ESI-MS analysis on a Shimadzu hybrid ion-trap–time-of-flight mass spectrometer (Shimadzu, Kyoto, Japan). The following instrument settings were applied for analysis: drying gas (N_2_) pressure—200 kPa; nebulizer gas (N_2_) flow—1.5 L/min; electrospray ionization (ESI) source potential—3.8 kV for negative polarity ionization and 4.5 kV for positive polarity ionization; temperature for the curved desolvation line (CDL) and heat block—200 °C; detector voltage—1.5 kV, detection range—100–900 *m/z*. The mass accuracy was below 4 ppm. We acquired and processed the data using Shimadzu LCMS Solution software v3.60.361 (Shimadzu, Kyoto, Japan).

### 2.6. Antiradical Activity

The 2,2-diphenyl-1-picrylhydrazyl (DPPH^•^) radical-scavenging effect of polyphenolic compounds **7**–**11** was determined as described in [24]. Polyphenolic compounds **7**, **8**, **9**, **10**, and **11** were added to DPPH^•^ solution in MeOH (10^−4^ M) at concentrations from 6 to 34 µM. The reacting mixture was kept in the dark for 30 min at room temperature. Then, we measured the absorbance at 517 nm using a Shimadzu UV 1240 spectrophotometer (Shimadzu, Kyoto, Japan). Equation (1) was used to calculate the DPPH^•^ radical-scavenging effect (%):

(1) $${\mathrm{DPPH}}^{•}\;\mathrm{scavenging}\;\mathrm{effect},\%=\frac{A_{0}-A_{x}}{A_{0}}$$

where *A*_0_ is the absorbance of DPPH^•^ solution of a blank sample (without polyphenolic compounds); *A_X_* is the absorbance of DPPH^•^ solution in the presence of different concentrations of polyphenolic compounds.

Quercetin was used as a reference compound. All experiments were performed in triplicate. We calculated the half-maximal scavenging concentration (SC_50_) for polyphenolic compounds by plotting the DPPH^•^ scavenging effect (%) against the concentrations of polyphenolic compounds. SC_50_ values are given as the mean value ± SEM.

### 2.7. Ferric Reducing Antioxidant Power (FRAP) Assay

We performed the FRAP assay as described in [29]. We prepared the FRAP reagent by mixing 2.5 mL of TPTZ (2,4,6-tris(2-pyridyl)-*s*-triazine) solution (10 mM) in 40 mM HCl and 25 mL of FeCl_3_ solution (20 mM) in acetate buffer solution (300 mM, pH 3.6). Polyphenolics **7**–**11** were added to 3 mL of FRAP reagent at concentrations from 6 to 34 µM. The mixture was kept in the dark at room temperature for 4 min. Then, we measured the absorbance at 595 nm using a Shimadzu UV 1240 spectrophotometer. Equation (2) was used to calculate the FRAP values for polyphenolic compounds **7**–**11**:

(2) $$\mathrm{FRAP}=\frac{C_{Fe}}{C_{x}}$$

where *C_Fe_* is the concentration of Fe^2+^ (µM) formed in the reaction; *C_X_* is the concentration of polyphenolic compounds in the reacting mixture.

The concentration of Fe^2+^ (µM) formed in the reaction was determined using the calibration curve obtained for different concentrations of FeSO_4_·7H_2_O.

### 2.8. Quantum-Chemical Modeling

We applied density functional theory (DFT) with the nonlocal exchange-correlation functional B3LYP [30], the polarization continuum model (PCM) [31], and split-valence basis set 6-311G(d), implemented in the Gaussian 16 package of programs [32] to perform the quantum-chemical calculations for compounds **9** and **10** in acetonitrile solvent. The molecular cavity was modeled according to unified force field (*radii = UFF*). The detailed conformational analysis of compounds **9** and **10** preceded the following calculations of their chiroptical properties.

The statistical weights (*g_im_*) of different conformations were calculated according to Equation (3):

(3) $$g_{im}=\frac{e^{\frac{-\Delta G_{im}}{RT}}}{\sum_{i}e^{\frac{-\Delta G_{im}}{RT}}}$$

where the summation was performed over all found stable conformations of the stereoisomer under study, and Δ*G_im_* = *G_i_* − *G*_m_; *G* = *E*_el_ + *G*_tr,T_ + *G*_rot,T_ + *G*_vib,T_ is the sum of electronic, translational, rotational, and vibrational contributions to the Gibbs free energy, respectively, calculated at temperature T = 298.15 K; the subscript “m” denotes the conformation, for which *G* is minimal. 

The excitation energies and the rotatory strengths were calculated using time-dependent density functional theory (TDDFT), cam-B3LYP functional theory [33], and a PCM model and basis set, used previously for conformational analysis. Each individual transition from the electronic ground state to the *i*-th calculated excited electronic state (1 ≤ *i* ≤ 115) was simulated as a Gaussian-type function. The bandwidths, taken at 1/*e* peak heights, were chosen to be σ = 0.24 eV.

The total theoretical ECD spectrum was obtained after statistical averaging over all selected conformations using Equation (4):

(4) $$\Delta \varepsilon_{calc}\left(\lambda \right)=\underset{i}{\sum }g_{i}\times \Delta \varepsilon_{i,\;\;calc}\left(\lambda \right)$$

where *i* denotes different conformations of the stereoisomer under study. Conformations with Gibbs free energies in the region of Δ*G_im_* ≤ 5 kcal/mol were accounted for.

### 2.9. Cell Line and Culture Conditions

The murine neuroblastoma cell line Neuro-2a (CCL-131) was purchased from American Type Culture Collection (ATCC^®^) (Manassas, VA, USA). Cells were cultured in Dulbecco’s Modified Eagle Medium (Biolot, St. Petersburg, Russia). The medium contained 10% fetal bovine serum (Biolot, St. Petersburg, Russia) and 1% penicillin/streptomycin (Biolot, St. Petersburg, Russia). Cells were incubated at 37 °C in a humidified atmosphere containing 5% (*v*/*v*) CO_2_.

### 2.10. Cell Viability Assay (MTT Method)

Stock solutions of polyphenolic compounds were prepared in DMSO at a concentration of 10 mM. We diluted the solutions of all tested compounds in PBS to a volume of 20 μL and added them to the wells of the plates at the following final concentrations: 0.01, 0.1, 1.0, and 10.0 µM (final concentration of DMSO <1%).

We incubated Neuro-2a cells (1 × 10^4^ cells/well) at 37 °C in a CO_2_ incubator for 24 h until they formed an adherent monolayer. Then, 20 μL of the tested solution was loaded onto the cells and incubated for 24 h. After incubation, the medium containing the polyphenolic test compounds was replaced by 100 μL of fresh medium. Then, we added 10 μL of MTT (3-(4,5-dimethylthiazol-2-yl)-2,5-diphenyltetrazolium bromide) (Sigma-Aldrich, St. Louis, MO, USA) stock solution (5 mg/mL) to each well and incubated the microplate for 4 h. Then, 100 μL of SDS-HCl solution (1 g SDS/10 mL dH_2_O/17 μL 6 M HCl) was added to each well, followed by incubation for 18 h. We measured the absorbance of the converted dye formazan on a Multiskan FC microplate photometer (Thermo Scientific, Waltham, MA, USA) at a wavelength of 570 nm [34]. We performed all experiments in triplicate and expressed the cytotoxic activity as percent of living cells.

### 2.11. In Vitro Model of PQ-, Rotenone-, and 6-OHDA-Induced Neurotoxicity 

After 24 h of adhesion, Neuro-2a cells (1 × 10^4^ cells/well) were treated with polyphenolic compounds at concentrations of 0.01–10 μM for 1 h. Subsequently, 1 mM of PQ, 10 μM rotenone or 80 μM of 6-OHDA (Sigma-Aldrich, St. Louis, MO, USA) were added. We used MTT assay to determine the percentage of living cells (cell viability) after 24 h of incubation. Cells incubated without inductors or with inductors were used as positive and negative control, respectively. The results are presented as percentages of the positive control value. 

### 2.12. Reactive Oxygen Species (ROS) Level Evaluation in PQ-Treated Cells

After 24 h of adhesion, Neuro-2a cells (1 × 10^4^ cells/well) were incubated with compounds (0.01–10 µM) for 1 h. Then, we added rotenone (10 μM), 6-OHDA (80 μM), or PQ (1 mM) to each well and incubated cells for 1 h, 1 h, or 3 h, respectively. To evaluate the level of intracellular ROS, we added 20 µL of 2,7-dichlorodihydrofluorescein diacetate solution (10 µM, H2DCF-DA, Molecular Probes, Eugene, OR, USA) to each well, so that the final concentration was 10 µM, and incubated the microplate at 37 °C for an additional 30 min. Quercetin was used as a reference compound.

### 2.13. Mitochondrial Membrane Potential (MMP) Detection

The cells were incubated for 1 h in a 96-well plate (1 × 10^4^ cells/well) with polyphenolic compounds (1 and 10 µM). Then, PQ (500 µM) was added, and the cell suspension was incubated for 1 h. Cells incubated without PQ and compounds were used as positive control, and cells with PQ alone were used as negative control. We added the tetramethylrhodamine methyl (TMRM) (Sigma-Aldrich, St. Louis, MO, USA) solution (500 nM) to each well, and incubated cells for 30 min at 37 °C. After the incubation, we measured the intensity of fluorescence with a PHERAstar FSplate reader (BMG Labtech, Ortenberg, Germany) at λ_ex_ = 540 nm and λ_em_ = 590 nm. We processed the data using MARS Data Analysis v3.01R2 (BMG Labtech, Ortenberg, Germany) and presented results as percentages of the positive control value.

### 2.14. Statistical Analysis

We carried out all the experiments in triplicate and performed Student’s t-test using SigmaPlot 14.0 (Systat Software Inc., San Jose, CA, USA) to determine statistical significance.

## 3. Results

### 3.1. Isolation and Structure Elucidation of Compounds ***10*** and ***11***

**Figure 1:**
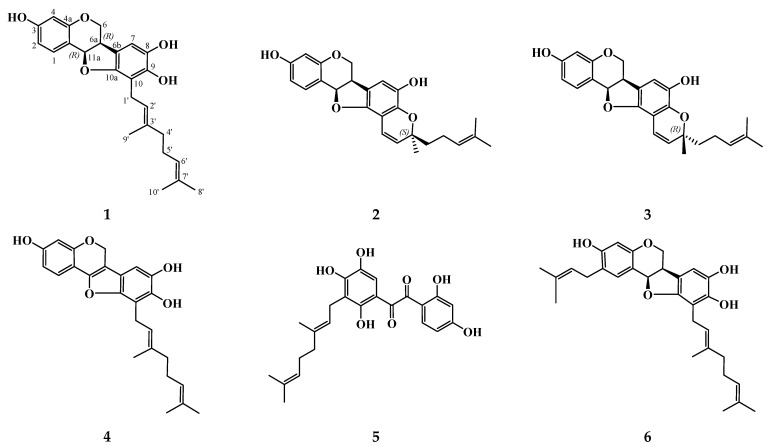

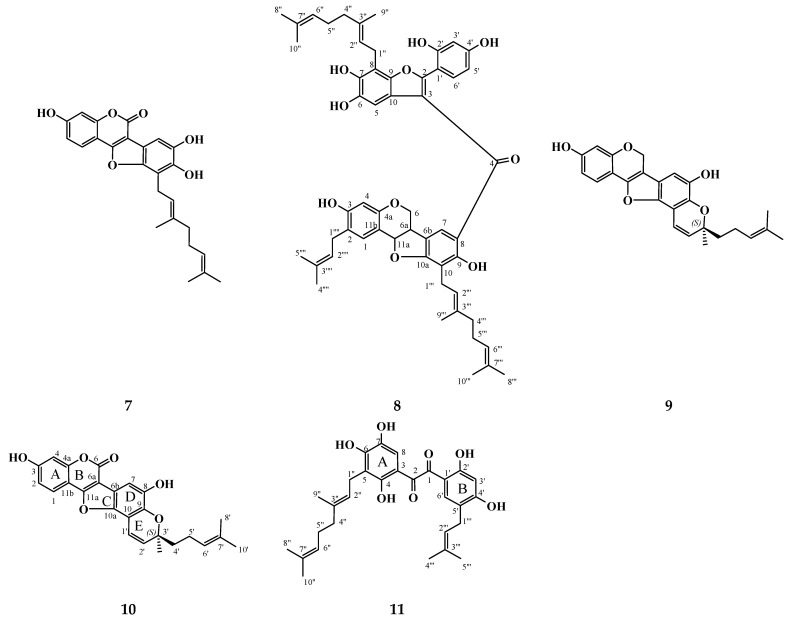
Structures of polyphenolic compounds isolated from *L. bicolor* root bark.

We managed to isolate a new coumestan **10** and stilbenoid **11** as well as previously known pterocarpans: (6a*R*,11a*R*)-6a,11a-dihydrolespedezol A_2_ (**1**), (6a*R*,11a*R*,3′*S*)-6a,11a-dihydrolespedezol A_3_ (**2**), 6a*R*,11a*R*,3′*R*-6a,11a-dihydrolespedezol A_3_ (**3**), (6a*R*,11a*R*)-2-isoprenyl-6a,11a-dihydrolespedezol A_2_ (**6**), pterocarpens: lespedezol A_2_ (**4**), lespedezol A_3_ (**9**), coumestan lespedezol A_6_ (**7**), stilbenoid bicoloketone (**5**), and dimeric flavonoid lespebicolin B (**8**) from *L. bicolor* root bark (Figure 1). Compounds **1**–**9** were identified by comparison of their HPLC-PDA-MS and NMR spectra with previously published data [21,22,24,26].

Compound **10** was obtained as a white amorphous powder. The molecular formulae of **10** was determined to be C_25_H_22_O_6_ based on the presence of [M-H]^-^ and [M+H]^+^ ions at *m/z* 417.1337 (calculated for C_25_H_21_O_6_^-^ 417.1344) and 419.1479 (calculated for C_25_H_23_O_6_^+^ 419.1489), respectively, in the HR-MS-ESI spectrum of **10**. The ^13^C spectrum of **10** contained 25 signals of carbon atoms (Table 1). Fifteen carbon atoms belonged to the coumestan skeleton (rings A–D), and 10 atoms formed a 3′-methyl-3′-isohexenylpyran ring (E). An ester carbonyl carbon signal was observed in the ^13^C NMR spectrum of **10** at *δ_C_* 159.0 and was assigned to C-6 of the coumestan skeleton [22]. The ^1^H NMR spectra of **10** showed the presence of an ABX spin system: the signals at *δ_H_* 7.85 (d, *J* = 8.5), 6.95 (d, *J* = 8.5), and 7.13 (s) were attributed to protons H-1, H-2, and H-4 of ring A, respectively (Table 1). A singlet signal at *δ_H_* 7.44 was ascribed to the H-7 proton (ring D of the coumestan skeleton). We also observed two broad singlet signals at *δ_H_* 6.54 and 5.50 due to OH-3 and OH-8 protons, respectively, in the ^1^H NMR spectra of **10** (Table 1). The ^1^H NMR spectra of **10** revealed signals at *δ_H_* 6.89 (1H, d, *J* = 9.9 Hz) and 5.78 (1H, d, *J* = 9.9 Hz) due to the H-1′ and H-2′ protons of the AX-type olefinic proton system, suggesting that **10** had an oxidatively cyclized geranyl side chain similar to the structures of compounds **2** and **9** (Table 1) [24]. 

We completely assigned all signals in the ^1^H and ^13^C NMR spectra of **10** on the basis of COSY, HMBC, and ROESY spectral data. Thus, compound **10** was determined to be a derivative of lespedezol A_6_ (**7**), previously isolated from *L. homoloba* but containing a cyclized geranyl side chain [22]. Compound **10** was named lespebicoumestan A. 

Compounds **9** and **10** had a 3′-methyl-3′-isohexenylpyran ring (E) and, hence, an asymmetric center at C-3′. Although lespedezol A_3_ (**9**) had previously been isolated from *L. homoloba* [21], the absolute configuration of the asymmetric center at C-3′ had not yet been determined. In order to determine the absolute configuration of the asymmetric center at C-3′ in compounds **9** and **10**, we compared their calculated theoretical ECD spectra with corresponding experimental ECD data. We used the cam-B3LYP exchange-correlation functional set [33] along with the 6-311G(d) basis set and polarization continuum model (PCM) [31], implemented in the Gaussian 16 suit of programs [32], to calculate the energies and rotatory strengths of vertical electronic transitions. First, conformational analysis was performed for each compound at the B3LYP/6-311G(d)_PCM level of theoretical modeling; optimized geometries and relative Gibbs free energies as well as statistical weights were thus obtained. 

The choice of the cam-B3LYP functional model for excited states is approved because it accounts for long-range interactions, to some extent better than the B3LYP functional model. Finally, statistically averaged ECD spectra were obtained as a weighted superposition of Gaussian-type functions and chosen for simulation of the Δε*_i_*(λ) function shapes for individual electronic transitions. The same value of σ = 0.24 eV for the bandwidths at 1/*e* peak heights was used. One hundred fifteen excited states were calculated for each conformation. 

**Figure 2:**
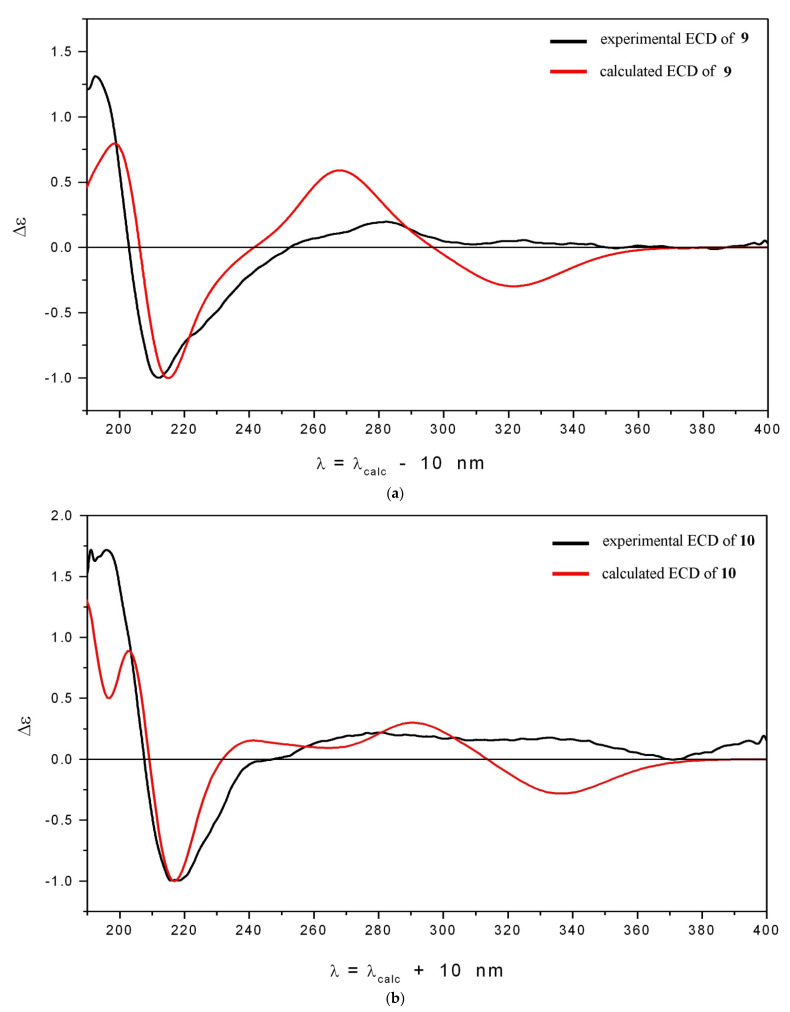
Experimental and calculated ECD spectra for compounds **9** (**a**) and **10** (**b**).

A comparison of the experimental and theoretical ECD spectra obtained for **9** and **10** is presented in Figure 2a,b. The positions and relative intensities of individual bands in the characteristic region 200 ≤ λ ≤ 300 nm are well-reproduced. The discrepancies in properties of Δε_calc_ (λ) and Δε_exp_ (λ) occur in the long-wave region λ ≥ 300 nm, caused to some extent by underestimation of the contribution to the Δε_calc_ (λ) from the E^+^ conformations (Schemes S60–S61, Figure S62, Supplementary data). Thus, we performed several simulations of the averaged ECD spectrum for **9**, in which relative amounts of the E^−^ and E^+^ conformations were varied manually. We found that the shapes, positions, and relative intensities of individual bands in the characteristic region 200 ≤ λ ≤ 300 nm are refractory to these variations, while they change dramatically in the λ ≥ 300 nm region Schemes S60–S61, Figure S62 (S61–S62, Supplementary data). We previously observed analogous behavior for compound **2** [24]. 

The good qualitative coincidence between theoretical and experimental ECD spectra allowed us to determine the absolute configuration of the asymmetric center at C-3′ in compounds **9** and **10** as C3′-*S.*

We obtained compound **11** as a yellow, amorphous powder. We elucidated the structure of the new compound using extensive spectroscopic analyses. The molecular formula C_29_H_34_O_7_ was confirmed by the presence of the [M-H]^−^ molecular ion at *m/z* 493.2217 (negative ion mode, calculated for C_29_H_33_O_7_^−^ 493.2232) and the [M+H]^+^ molecular ion at *m/z* 495.2401 (positive ion mode, calculated for C_29_H_35_O_7_^+^ 495.2377) in the HR-ESI-MS spectrum of **11**. The ^13^C NMR spectrum of **11** showed the presence of 12 carbon atoms of two aromatic rings, 10 carbon atoms of a geranyl side chain, 5 carbon atoms of an isoprenyl side chain, and 2 carbon atoms of two carbonyl groups. The ^1^H NMR spectrum of **11** exhibited resonances at *δ_H_* 6.44 (1H, s) and 7.18 (1H, s) of protons H-3′ and H-6′, respectively (ring B), and a singlet at *δ_H_* 6.84 (1H) was attributed to H-8 (ring A) (Table 2). The downfield-shifted chemical shift values of hydroxyl groups OH-4 and OH-2′ at *δ_H_* 11.92 (1H, s) and 11.59 (1H, s) confirmed that they formed hydrogen bonds with carbonyl groups at C-2 and C-1, respectively. The signals of the geranyl side chain protons were observed at *δ_H_* 3.51 (2H, d, *J* = 7.3 Hz, H-1″), 5.33 (1H, t, *J* = 7.3 Hz, H-2″), 2.09 (2H, m, H-4″), 2.13 (2H, m, H-5″), 5.07 (1H, m, H-6″), 1.68 (3H, s, H-8″), 1.84 (3H, s, H-9″), and 1.60 (3H, s, H-10″) (Table 2). The geranyl substituent was determined to be located at C-5 because in the HMBC spectrum of **11** we observed cross-peaks between the proton signal at *δ_H_* 3.51 (1H, d, *J* = 7.3 Hz) of H-1″ and the C-4, C-5, and C-6 carbon signals at *δ_C_* 158.9, 115.3, and 152.5, respectively (Table 2). The signals of the isoprenyl side chain protons were observed at *δ_H_* 3.23 (2H, d, *J* = 7.2 Hz, H-1‴), 5.20 (1H, t, *J* = 7.2 Hz, H-2‴), 1.73 (3H, s, H-4‴), and 1.71 (3H, s, H-5‴) (Table 2). The isoprenyl substituent was determined to be located at C-5′ because in the HMBC spectrum of **11** we observed cross-peaks between the proton signal at *δ_H_* 3.23 (1H, d, *J* = 7.2 Hz) of H-1‴ and the C-4′, C-5′, and C-6′ carbon signals at *δ_C_* 163.7, 119.9, and 133.9, respectively (Table 2). Thus, compound **11** was shown to be a stilbenoid, and the structure of **11** differed from that of bicoloketone (**5**) only by the presence of an additional isoprenyl side chain at C-5′. Compound **11** was named 5′-isoprenylbicoloketone. 

### 3.2. Antioxidant Activity of Polyphenolic Compounds

The data on antioxidant activity (DPPH^•^-scavenging effect and FRAP assay) of compounds **1**–**6** have been previously published in [24]. Here, we evaluated the DPPH^•^-scavenging effect and FRAP of polyphenolic compounds **7**–**11** isolated from *L. bicolor*. In the FRAP assay, polyphenolic antioxidants reduced the light blue Fe^3+^–TPTZ complex to the dark blue Fe^2+^–TPTZ complex. The change in color resulted in an increase in absorbance at 595 nm. The results of both tests are shown in Table 3. 

Compounds **1**–**11** exhibited a moderate DPPH-scavenging effect, which was comparable to the effect of ascorbic acid but smaller than that of quercetin (Table 3). Lespedezol A_2_ (**4**) and lespedezol A_3_ (**9**) possessed the most-significant DPPH-scavenging effect and FRAP among compounds **1**-**11** (Table 3) [24]. In the FRAP assay, all tested polyphenolics also showed moderate effects but were less active than quercetin and ascorbic acid. Lespedezol A_2_ (**4**) was the most effective in the FRAP assay.

### 3.3. Cytotoxicity of Polyphenolic Compounds from L. bicolor against Neuro-2a Cells

The cytotoxic activity of 11 polyphenolic compounds from *L. bicolor* root bark was evaluated using the MTT assay (Table 4). The cytotoxicity of the isolated compounds was determined on neuroblastoma Neuro-2a cells. Compounds **3** (EC_50_ = 44.0 µM), **6** (EC_50_ = 40.6 µM), and **11** (EC_50_ = 44.0 µM) possessed moderate toxicity. Polyphenolics **1**, **2**, **4**, **9**, and **11** showed low toxicity (EC_50_ = 72–87 µM). It should be noted that compound **4** has previously been shown to be rather cytotoxic against three cancer cell lines (human triple-negative breast cancer HTB-19, esophageal squamous cell carcinoma Kyse-30, and hepatocellular carcinoma HEPG-2) and two normal cell lines (retina pigmented epithelium cells RPE-1 and human embryonic kidney cells HEK-293) [11]. The other compounds did not demonstrate cytotoxic effects at concentrations up to 100 µM.

### 3.4. Influence on Viability and ROS Level in PQ-Treated Neuro-2a Cells 

**Figure 3:**
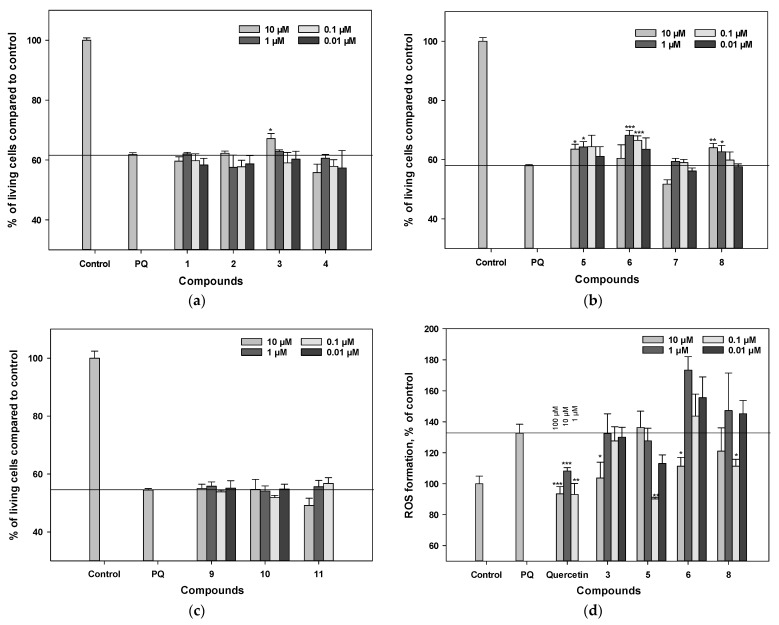

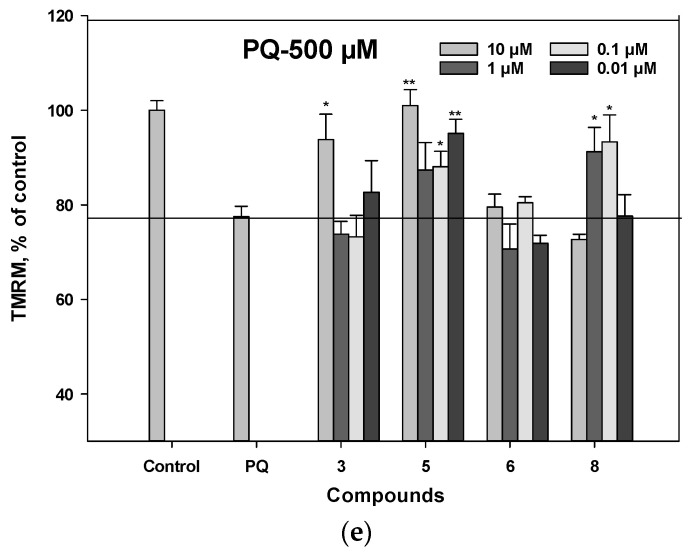
The influence of polyphenolic compounds from *L. bicolor* on cell viability (**a**–**c**), ROS levels (**d**), and mitochondrial membrane potential (**e**) in Neuro-2a cells treated with PQ (1 mM). The percentage of living cells treated with various compounds and PQ was measured by MTT assay. Each bar represents the mean ± SEM of three independent replicates. (*), (**), and (***) indicate, respectively, *p* < 0.05, *p* < 0.005, and *p* < 0.001 versus PQ-treated cells. The difference between control and PQ-treated cells was considered significant.

The percentage of living cells after treatment with neurotoxin was assessed by the MTT test. The percentage of living cells after treatment with PQ significantly increased when polyphenolic compounds **3**, **5**, **6**, and **8** were added (Figure 3a–c). The neuroprotective effect of compound **6**, measured by the MTT assay, was detected in the concentration range of 0.01–1 μM, and at a concentration of 1 μM this compound increased the percentage of living cells by 17%. Compound **3** increased the percentage of living cells after treatment with PQ by 8% at a concentration of 10 μM in the MTT assay. Compounds **5** and **8** increased the percentage of living cells after PQ treatment by 8–10% at concentrations of 1 and 10 μM in the MTT assay. Compounds **1**, **2**, **4**, and **9**–**11** did not exhibit any effect on PQ-treated Neuro-2a cell viability in this test. Polyphenolics from *L. bicolor* did not significantly reduce ROS levels in PQ-treated cells (Figure 3d).

### 3.5. Mitochondrial Membrane Potential (MMP) Detection

We studied the effect of polyphenolic compounds from *L. bicolor* on PQ-induced mitochondrial dysfunction. The 23% decrease in tetramethylrhodamine methyl (TMRM) fluorescence after a 1 h exposure of Neuro-2a cells with PQ indicates that PQ causes depolarization of the mitochondrial membrane (Figure 3e). Among the tested compounds, pterocarpan **3** and stilbenoid **5** at a concentration of 10 µM were the most effective in this assay and increased the value of mitochondrial membrane potential by 16% and 23%, respectively.

### 3.6. Influence on Viability and ROS Levels in 6-OHDA-Treated Neuro-2a Cells

**Figure 4:**
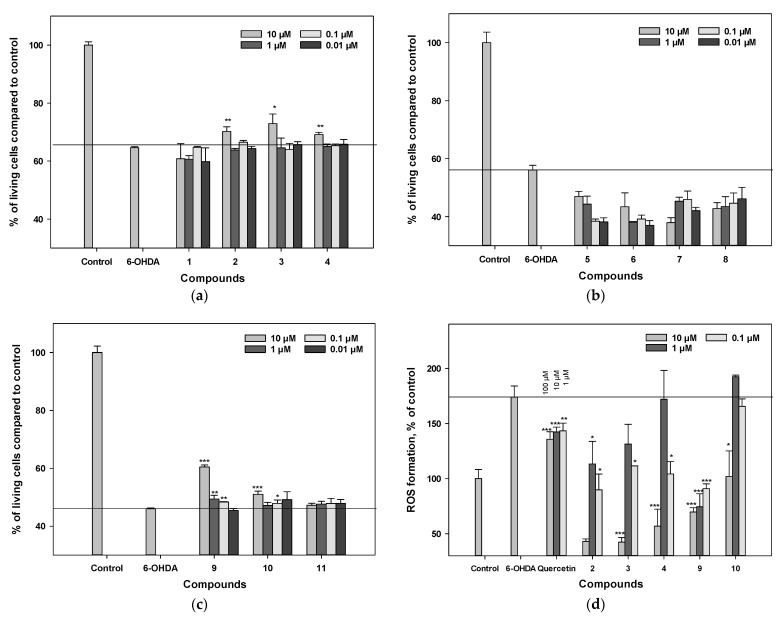
The influence of polyphenolic compounds from *L. bicolor* on cell viability (**a**–**c**) and ROS levels (**d**) in Neuro-2a cells treated with 6-OHDA (80 µM). The percentage of living cells treated with compounds and 6-OHDA was measured by MTT assay. Each bar represents the mean ± SEM of three independent replicates. (*), (**), and (***) indicate, respectively, *p* < 0.05, *p* < 0.005, and *p* < 0.001 versus 6-OHDA-treated cells. The difference between control and 6-OHDA-treated cells was considered significant.

We evaluated the effects of the polyphenolic-compound set on cell viability in a 6-OHDA-induced neurotoxicity model. The percentage of living Neuro-2a cells after 6-OHDA treatment increased from 45% to 65% in the presence of polyphenolic compounds, compared with the control (Figure 4a–c). Pre-treatment of cells with the test compounds for 1 h before 6-OHDA addition provided an increase in the percentage of living cells with varying levels of statistical confidence.

Compounds **9** and **10** increased the percentage of living Neuro-2a cells after 6-OHDA treatment by 31.4% and 10.9% versus 6-OHDA-treated cells, respectively (*p* < 0.05). Compounds **2**, **3**, and **4** increased the viability of 6-OHDA-treated cells by 8.7%, 12.9%, and 7.0%, respectively (*p* < 0.05). The other compounds did not show significant improvement in this assay. Polyphenolic compounds **2**–**4**, **9**, and **10** significantly decreased ROS levels in 6-OHDA-treated cells (Figure 4d). Pterocarpans **2** and **3** were the most-active in this test and decreased the ROS level 4.5 times compared to that of 6-OHDA-treated cells. All tested polyphenolic compounds decreased the intracellular ROS level much more effectively than quercetin (Figure 4d).

### 3.7. Influence on Viability and ROS Levels in Rotenone-Treated Neuro-2a Cells

**Figure 5:**
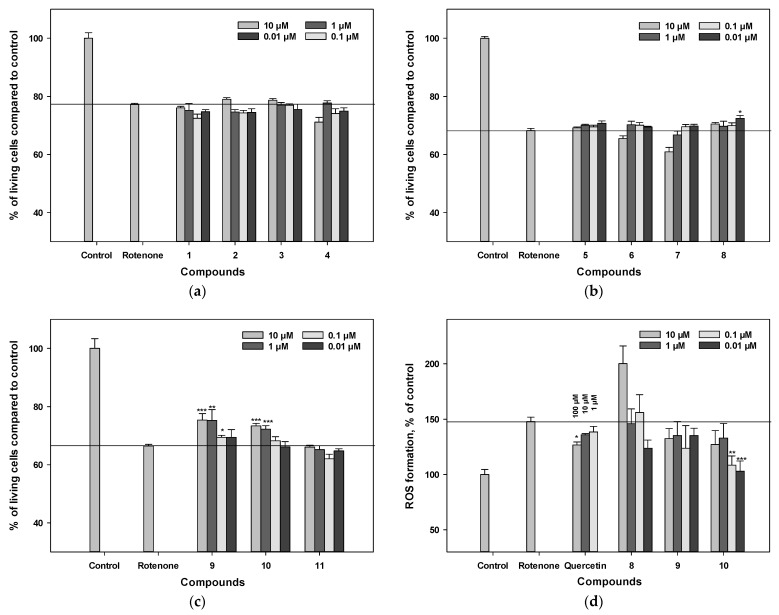
The influence of polyphenolic compounds from *L. bicolor* on cell viability (**a**–**c**) and ROS levels (**d**) in Neuro-2a cells treated with rotenone (10 µM). The percentage of living cells treated with compounds and rotenone was measured by the MTT assay. Each bar represents the mean ± SEM of three independent replicates. (*), (**), and (***) indicate, respectively, *p* < 0.05, *p* < 0.005, and *p* < 0.001 versus rotenone-treated cells. The difference between control and rotenone-treated cells was considered significant.

We examined the effect of polyphenolic compounds from *L. bicolor* on cell viability in a rotenone-induced neurotoxicity model. The percentage of living Nuero-2a cells treated with rotenone was 68% compared to the control (Figure 5a–c). Pre-treatment of cells with polyphenolic compounds from *L. bicolor* for 1 h before rotenone addition provided an increase in the percentage of living Neuro-2a cells with different levels of statistical confidence.

Compounds **9** and **10** increased the percentage of living Neuro-2a cells after treatment with rotenone by 10.4% and 13.2%, respectively, compared to rotenone-treated cells (*p* < 0.05). The other compounds did not show significant improvement in this assay (Figure 3a–d).

## 4. Discussion

We continued to study the chemical composition of polyphenolic compounds from *L. bicolor* root bark and isolated a new coumestan, lespebicoumestan A (**10**), and a stilbenoid, 5′-isoprenylbicoloketon (**11**), as well as previously known pterocarpans **1**–**3**, and **6**; pterocarpens **4** and **9**; coumestan **7**; stilbenoid **5;** and dimeric flavonoid **8** (Figure 1). There was a considerable difference between the chemical compositions of polyphenolic compounds of *L. bicolor* growing in the Primorskiy region (Russian Far East) and in the Republic of Korea. Lee P.J. and coworkers isolated from *L. bicolor* 11 pterocarpans, 2 coumestans, and 2 arylbenzofurans with isoprenyl and geranyl substituents in their structures [25,27,28]. These compounds contained a methoxy group at C-1, whereas the C-1 position in pterocarpans and coumestans from stem bark and root bark of *L. bicolor* collected in the Primorskiy region was unsubstituted [24,26]. Besides, some pterorarpans and coumestans from *L. bicolor* growing in the Russian Far East had a 3′-methyl-3′-isohexenylpyran ring (E), presumably formed by oxidative cyclization of a geranyl side chain. However, pterocarpans and coumestans from *L. bicolor* growing in the Republic of Korea contained a 3′,3′-dimethylpyran ring (E) produced by oxidative cyclization of an isoprenyl side chain. In contrast to Far-Eastern *L. bicolor*, Korean *L. bicolor* did not contain stilbenoids and dimeric flavonoids. 

Being natural antioxidants, pterocarpans and coumestans are promising candidates for the study of neuroprotective properties [10,11,25]. Nerve cells treated with various inducers of oxidative stress (PQ, 6-OHDA, rotenone) are often used as one of the generally accepted models for studying neurotoxic disorders, including PD [35,36]. 

In our study, pterocarpans **3** and **6**, stilbenoid **5**, and dimeric flavonoid **8** significantly increased the percentage of living Neuro-2a cells after treatment with PQ, but only pterocarpan **6** slightly decreased the ROS level in PQ-treated cells, which is in accordance with its rather high activity in the DPPH^•^ and FRAP tests [24]. It is known that the effect of PQ on neurons is accompanied by impaired functioning of mitochondria due to changes in mitochondrial membrane permeability, membrane potential, and depolarization of mitochondrial membranes [37]. We presumed that these compounds could also increase cell viability by preventing depolarization of the mitochondrial membrane. In fact, pterocarpan **3** and stilbenoid **5** were shown to effectively increase the mitochondrial membrane potential of PQ-treated neuronal cells.

We also examined the effects of polyphenolic compounds on cell viability in a 6-OHDA-induced neurotoxicity model. We showed that pterocarpans **2** and **3**, containing a 3′-methyl-3′-isohexenylpyran ring (E); pterocarpens **4** and **9**, with a double bond between C-6a and C-11a; and lespebicoumestan A (**10**) significantly increased the percentage of living Neuro-2a cells and decreased ROS levels after treatment with 6-OHDA. Notably, compounds **9** and **10** both contain a 3′-methyl-3′-isohexenylpyran ring (E) and a double bond between C-6a and C-11a. Pterocarpans **2** and **3** were the most active in this assay and decreased the ROS level 4.5 times compared to 6-OHDA-treated cells. Notably, polyphenolic compounds from *L. bicolor* decreased the level of intracellular ROS much more effectively than quercetin (Figure 4d). Thus, pterocarpans and coumestans with an additional 3′-methyl-3′-isohexenylpyran ring demonstrated the most-significant activity.

We also studied the effect of polyphenolic compounds from *L. bicolor* on cell viability in a rotenone-induced neurotoxicity model. Pre-treatment of cells with polyphenolic compounds from *L. bicolor* before rotenone addition resulted in an increase in the percentage of living Neuro-2a cells, with compounds **9** and **10** being the most active. The presence of a 3′-methyl-3′-isohexenyl pyran ring (E) and a double bond between C-6a and C-11a in **9** and **10** may be responsible for the increase in cell viability. The other compounds did not show significant improvement of cell viability in this assay. 

Previously, researchers from the College of Pharmacy, the Research Institute of Pharmaceutical Science and Technology (The Republic of Korea), and the Korea Research Institute of Standards and Science demonstrated that pterocarpan-type compounds (1-methoxylespeflorin G11, bicolosin A, 1-methoxyerythrabyssin II, 8-methoxybicolosin C, 2-geranyl-1-methoxylespeflorin G11, and 2-geranylbicolosin A) exhibited significant neuroprotective effects against glutamate neurotoxicity in neuronal HT22 hippocampal cells [25]. The compound 2-geranyl-1-methoxylespeflorin G11—which has a geranyl group at C-2, a prenyl group at C-10, and a methyl group at C-8—was shown to attenuate apoptosis in HT22 cells by inhibiting intracellular ROS generation and mitochondrial dysfunction. Although in our study coumestan **10** effectively increased the percentage of living Neuro-2a cells after treatment with 6-OHDA- and rotenone, arylbenzofurans and coumestan isolated from Korean *L. bicolor* exhibited no protective effects [25].

Considering that isoflavonoids can quickly penetrate the blood–brain barrier [38], such compounds may be prospective agents in the treatment of PD.

## 5. Conclusions

Thus, pterocarpans **2**, **3**, and **6** and pterocarpens **4** and **9**, as well as coumestan **10** from *L. bicolor* effectively protected PQ- and 6-OHDA-treated Neuro-2a cells from oxidative stress. The effect of polyphenolic compounds **3** and **5** from *L. bicolor* is mainly due to their ability to impair PQ-induced depolarization of the mitochondrial membrane, whereas compounds **2**–**4**, **9**, and **10** decreased ROS levels in 6-OHDA-treated Neuro-2a cells more effectively than quercetin. The effect of polyphenolic compounds on the viability of rotenone-treated cells was less dramatic.

## Figures and Tables

**Table 1:** ^1^H (700 MHz), ^13^C (175 MHz), HMBC, COSY, and ROESY NMR data for compound **10** (*δ* in ppm, *J* in Hz, CDCL_3_).

Position	^13^C	^1^H	HMBC	COSY	ROESY
1	122.7	7.85, d, *J* = 8.5, 1H	C-3, 4, 4a	H-2	H-2
2	113.6	6.95, d, *J* = 8.5, 1H	C-3, 4, 11b	H-1	H-1
3	159.2				
4	104.0	7.13, s, 1H	C-2, 3, 4a, 11b		
4a	154.8				
6	159.0				
6a	103.8				
6b	116.0				
7	105.4	7.44, s, 1H	C-6a, 6b, 8, 9, 10, 10a		OH-8
8	143.1				
9	138.7				
10	106.4				
10a	145.2				
11a	160.1				
11b	106.3				
1′	115.9	6.89, d, *J* = 9.9, 1H	C-8, 9, 10, 10a, 3′, 9′	H-2′	H-2′
2′	130.6	5.78, d, *J* = 9.9, 1H	C-9, 10, 3′, 4′, 9′	H-1′	H-1′, 4′, 5′, 9′
3′	80.6				
4′	40.9	1.78, m, 1H	C-2′, 3′, 5′, 6′, 9′	H-5′	H-2′, 5′, 9′
		1.84, m, 1H	C-2′, 3′, 5′, 6′, 9′	H-5′	H-2′, 5′, 9′
5′	22.8	2.13, m, 1H	C-4′, 6′, 7′	H-4′, 6′	H-2′, 4′, 9′
		2.15, m, 1H	C-4′, 6′, 7′	H-4′, 6′	H-2′, 4′, 9′
6′	123.6	5.10, t, *J* = 6.9, 1H	C-4′, 6′, 8′, 10′	H-5′, 8′, 10′	H-5′, 8′
7′	132.2				
8′	25.6	1.67, s, 3H	C-4′ (weak), 6′, 7′, 10′	H-6′	H-6′
9′	26.3	1.50, s, 3H	C-2′, 3′, 4′, 5′ (weak)		H-2′, 4′, 5′
10′	17.6	1.57, s, 3H	C-4′ (weak), 6′, 7′, 8′	H-6′	
OH-3		6.54, bs, 1H			
OH-8		5.50, bs, 1H	C-7		H-7

**Table 2:** ^1^H (700 MHz), ^13^C (175 MHz), HMBC, COSY, and ROESY NMR data for compound **11** (*δ* in ppm, *J* in Hz, CDCL_3_).

Position	^13^C	^1^H	HMBC	COSY	ROESY
1	194.7				
2	194.6				
3	109.1				
4	158.9				
5	115.3				
6	152.5				
7	136.8				
8	113.7	6.84, s, 1H	C-2, 4, 6, 7		OH-7
1′	111.1				
2′	165.2				
3′	104.3	6.44, s, 1H	C-1′, 2′, 4′, 5′		
4′	163.7				
5′	119.9				
6′	133.9	7.18, s, 1H	C-1, 1′, 2′, 5′, 1‴		H-1‴
1″	22.0	3.51, d, *J* = 7.3, 2H	C-4, 5, 6, 2″, 3″	H-2″, 9″	H-2″, 9″, OH-6
2″	120.5	5.33, t, *J* = 7.3, 1H	C-1″	H-1″, 9″	H-1″, 4″, OH-6
3″	139.7				
4″	39.7	2.09, m, 2H	C-2″, 3″, 5″, 6″		H-2″, 6″
5″	26.4	2.13, m, 2H	C-3″, 6″, 7″	H-6″	H-6″
6″	123.8	5.07, m, 1H		H-5″, 8″, 10″	H-4″, 5″
7″	132.1				
8″	25.7	1.68, s, 3H	C-6″, 7″, 10″	H-6″	
9″	16.3	1.84, s, 3H	C-2″, 3″, 4″	H-1″, 2″	H-1″
10″	17.7	1.60, s, 3H	C-6″, 7″, 8″	H-6″	
1‴	29.1	3.23, d, *J* = 7.2, 2H	C-4′, 5′, 6′, 2‴, 3‴	H-2‴, 4‴, 5‴	H-2‴, 4‴, 5‴, 6′, OH-4′
2‴	120.8	5.20, t, *J* = 7.2, 2H		H-1‴, 4‴, 5‴	H-1‴, 5‴
3‴	136.4				
4‴	25.7	1.73, s, 3H	C-2‴, 3‴, 5‴	H-1‴, 2‴	H-1‴
5‴	17.9	1.71, s, 3H	C-2‴, 3‴, 4‴	H-1‴, 2‴	H-1‴, 2‴
OH-4		11.92, s, 1H	C-3, 4, 5		
OH-6		6.68, s, 1H	C-5, 6, 7		H-2″, 1″, OH-7
OH-7		5.05, bs, 1H			H-8, OH-6
OH-2′		11.59, s, 1H	C1′, 2′, 3′, 4′		
OH-4′		6.11, s, 1H	C-3′, 4′, 5′		H-1‴

**Table 3:** DPPH-scavenging activity and FRAP of compounds **7**–**11**.

Compound	DPPH-Scavenging Effect	FRAP
	SC_50_ µM, 30 min	mol (Fe^2+^)/mol (polyphenolic compound X)
Quercetin	8.9 *±* 0.2	5.11 *±* 0.31
Ascorbic acid	30.5 *±* 2.1	3.43 *±* 0.33
**1**	24.3 *±* 3.7 *	1.78 *±* 0.16 ***
**2**	25.0 *±* 2.2 **	1.31 *±* 0.02 **
**3**	23.7 *±* 2.1 **	1.60 *±* 0.08 **
**4**	18.0 *±* 0.2 ***	2.78 *±* 0.18 **
**5**	26.1 *±* 3.0 *	0.86 *±* 0.04 *
**6**	21.8 *±* 2.0 *	1.43 *±* 0.06 **
**7**	25.7 *±* 3.4 *	1.83 *±* 0.16 *
**8**	26.7 *±* 3.8 *	1.95 *±* 0.19 *
**9**	13.5 *±* 1.2 **	0.75 *±* 0.05 **
**10**	22.7 *±* 3.5 *	1.75 *±* 0.20 *
**11**	19.4 *±* 2.2 **	1.45 *±* 0.08 **

^1^ Data are presented as the mean ± SEM, n = 3. *** *p* < 0.001, ** *p* < 0.005, and * *p* < 0.05 compared to quercetin. ^2^ Data for compounds **1**–**6** have been previously published in [24].

**Table 4:** Cytotoxic activity of polyphenolic compounds **1**–**11** from *L. bicolor*.

Compound	EC_50_, µM
**1**	72.0
**2**	75.0
**3**	44.0
**4**	76.0
**5**	100.0
**6**	40.6
**7**	>100
**8**	>100
**9**	75.0
**10**	44.0
**11**	87.0

## Data Availability

The data are contained within the article and Supplementary Materials.

## Supplementary Materials

The following supporting information can be downloaded at: https://www.mdpi.com/article/10.3390/antiox11040709/s1, Figures S1–S2: Mass-spectra of compound **10**; Figures S3–S25: NMR spectra of compound **10**; Figures S26–S27: Mass-spectra of compound **11**; Figures S28–S57: NMR spectra of compound **11**; Figure S58: The geometrical structure and atom numeration for compounds **9** and **10**; Scheme S59: Abbreviations for conformations of compounds **9** and **10**; Scheme S60: The influence of inversion of ring B on the geometrical structure of compound **9**; Scheme S61: Equation for recalculation of the relative contributions of the E^−^ and E^+^ conformations to the ECD spectra of compounds **9** and **10**; Figure S62: The influence of variation in the relative amounts of E^−^ and E^+^ conformations on the shape of the statistically averaged scaled ECD spectrum of compound **9**.
