# Supplementary material for: Polyphenolic Compounds from Lespedeza bicolor Protect Neuronal Cells from Oxidative Stress
Source: Antioxidants (Basel). 2022 Apr 3;11(4):709. doi: 10.3390/antiox11040709 (PMC9025851; doi:10.3390/antiox11040709)
Supplement: Supplementary file 1 [file antioxidants-11-00709-s001.zip › antioxidants-1647270-supplementary.pdf]

Supplementary materials for manuscript

Polyphenolic Compounds from *Lespedeza bicolor* Prevent Neuronal Cells from Oxidative Stress

Darya V. Tarbeeva[1], Evgeny A. Pislyagin[1], Ekaterina S. Menchinskaya[1], Dmitrii V. Berdyshev[1], Anatoliy I. Kalinovskiy[1], Valeria P. Grigorchuk[2], Natalia P. Mischenko[1], Dmitry L. Aminin[1],Sergey A. Fedoreyev[1]

[1]G.B. Elyakov Pacific Institute of Bioorganic Chemistry, Far-Eastern Branch of the Russian Academy of Science, 690022 Vladivostok, Russia; pislyagin@hotmail.com (E.P.), ekaterinamenchinskaya@gmail.com (E.M.), berdyshev@piboc.dvo.ru (D.B.), kaaniv@piboc.dvo.ru (A.K.), daminin@piboc.dvo.ru (D.A.), fedoreev-s@mail.ru (S.F.)

[2]Federal Scientific Center of the East Asia Terrestrial Biodiversity, Russian Academy of Sciences, 690022 Vladivostok, Russia; kera1313@mail.ru (V.G.)

*Correspondence: tarbeeva1988@mail.ru (D.T.); Tel.: +791434464441

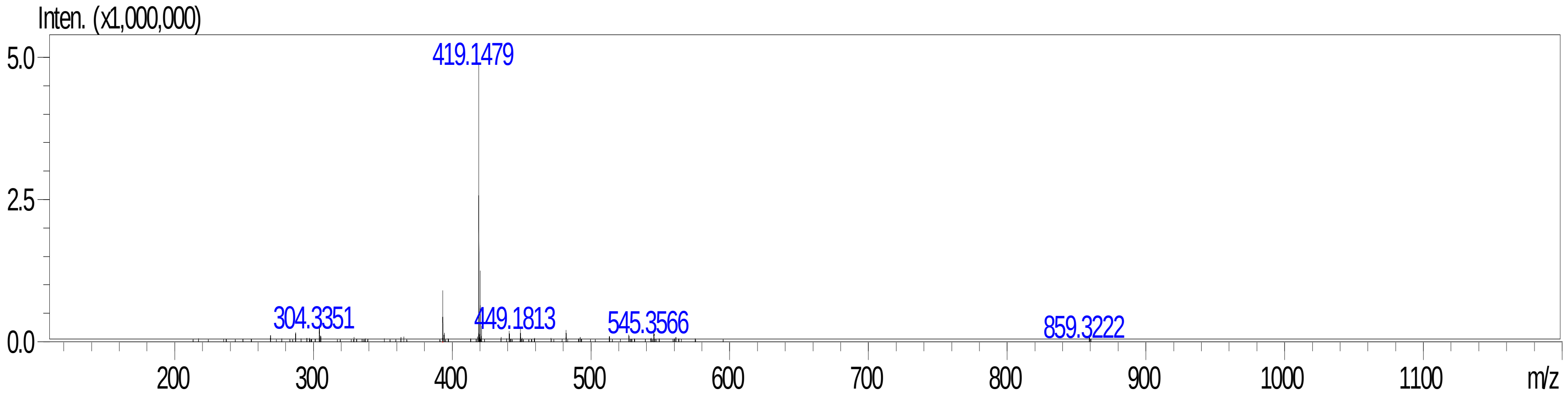

Figure S1. HR-ESI mass-spectrum of **10** $[M+H]^+$.

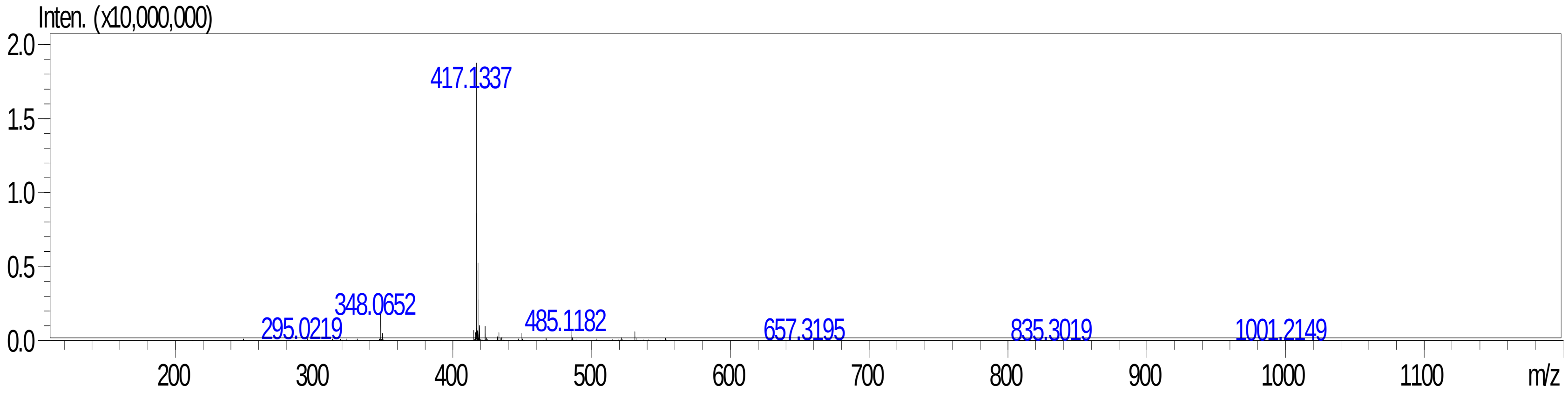

Figure S2. HR-ESI mass-spectrum of **10** [M-H]$^-$.

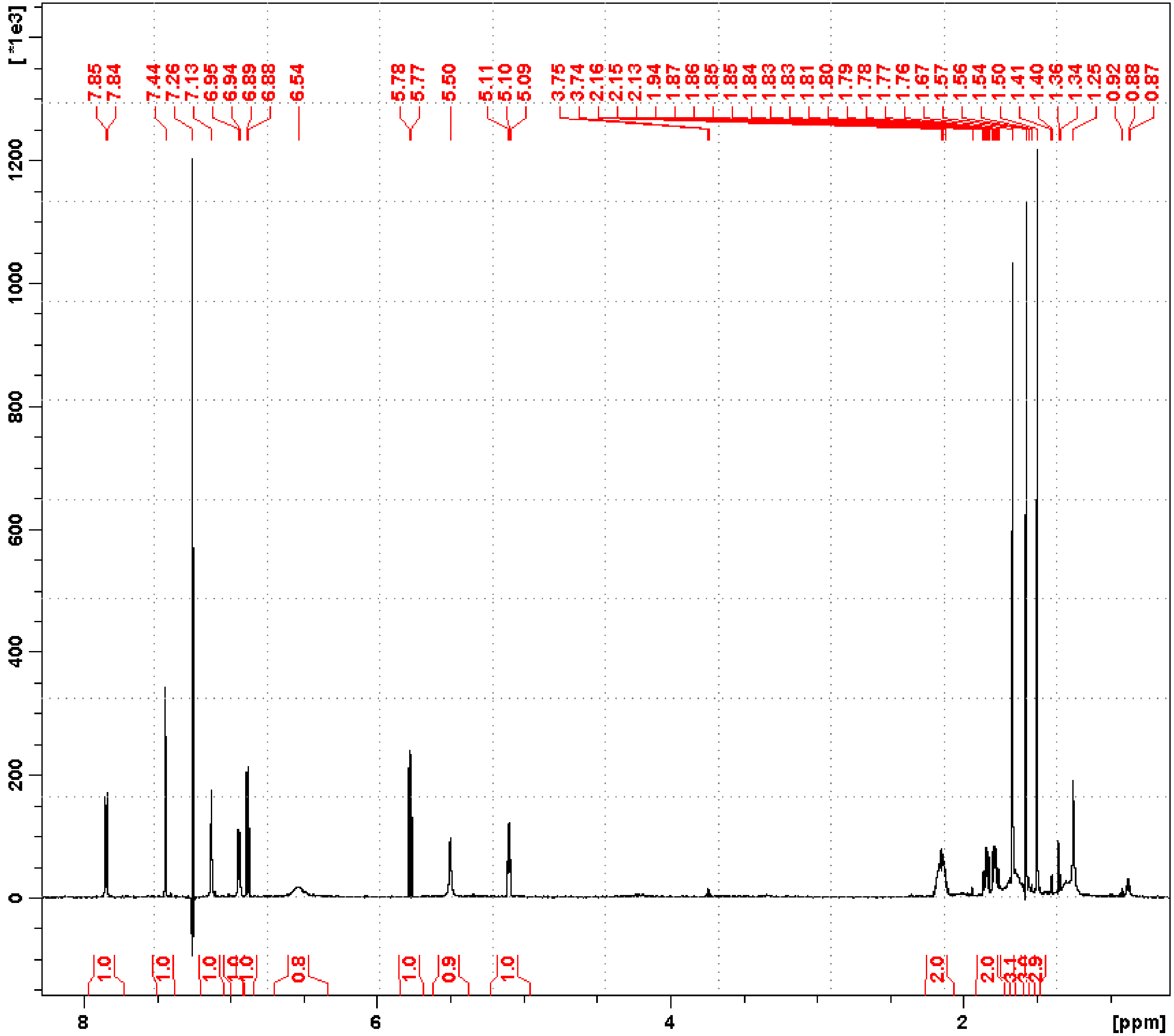

Figure S3. ^{1}H NMR spectrum of **10**.

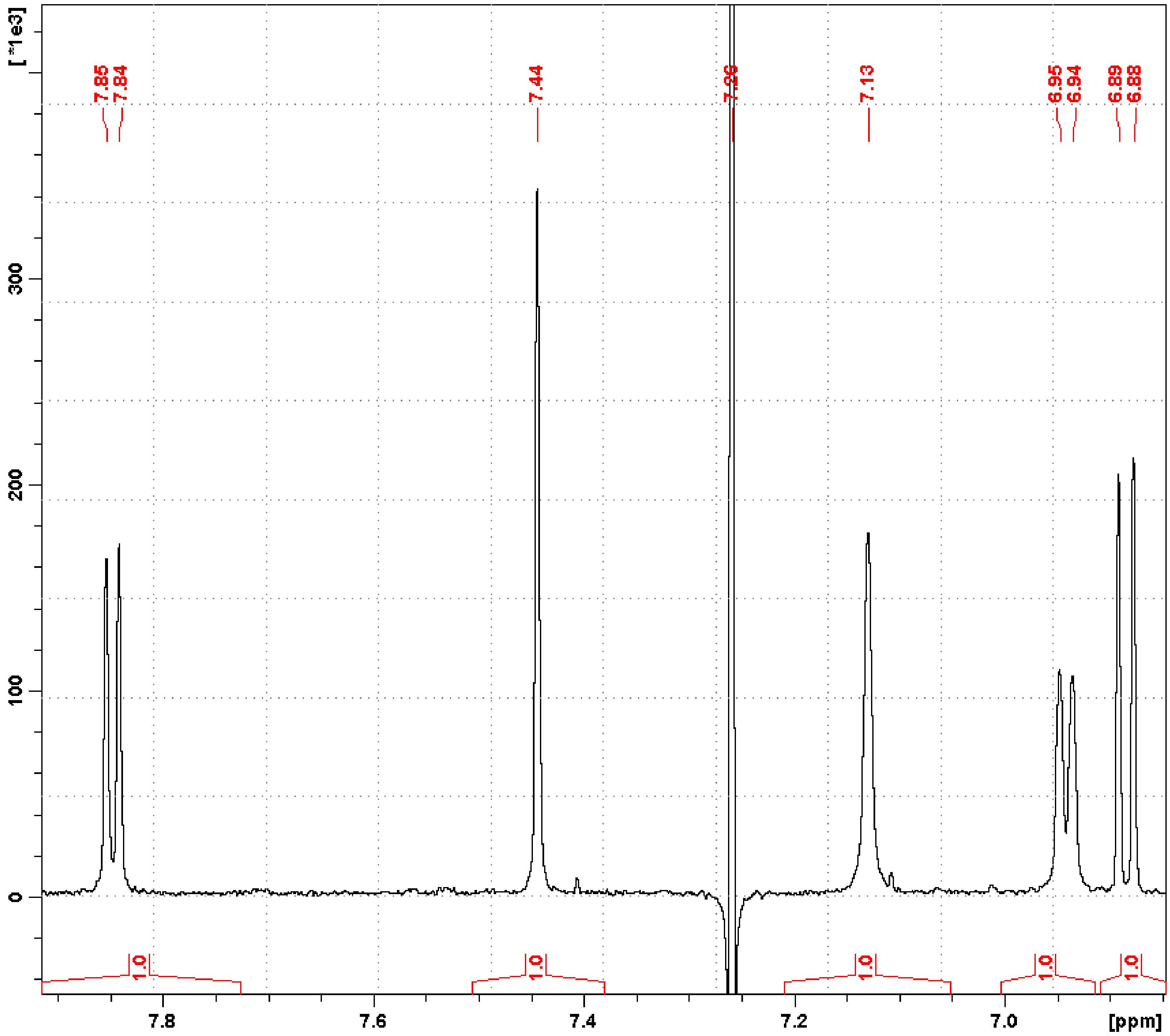

Figure S4. ^{1}H NMR spectrum of **10** (enlarged).

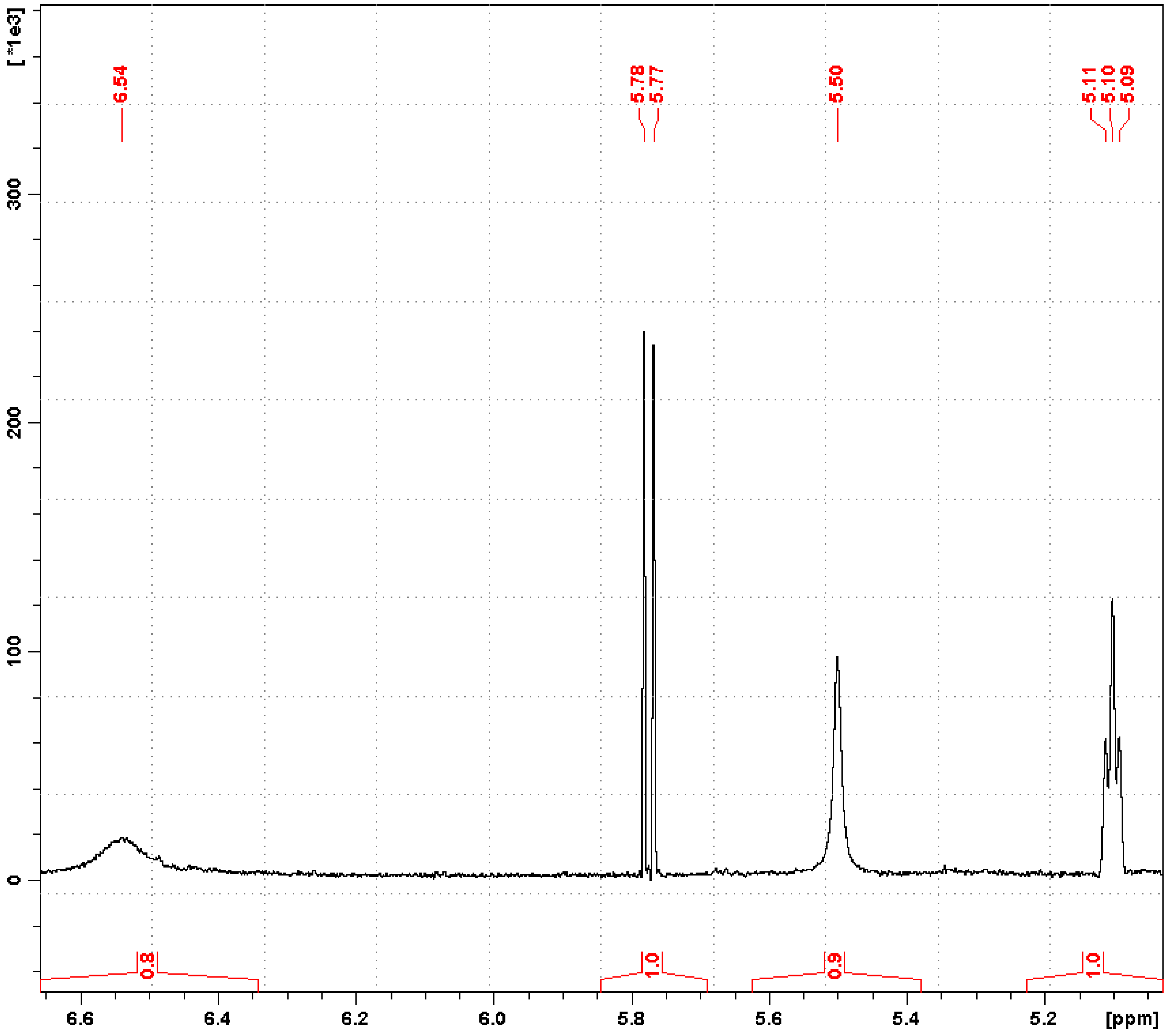

Figure S5. ^{1}H NMR spectrum of **10** (enlarged).

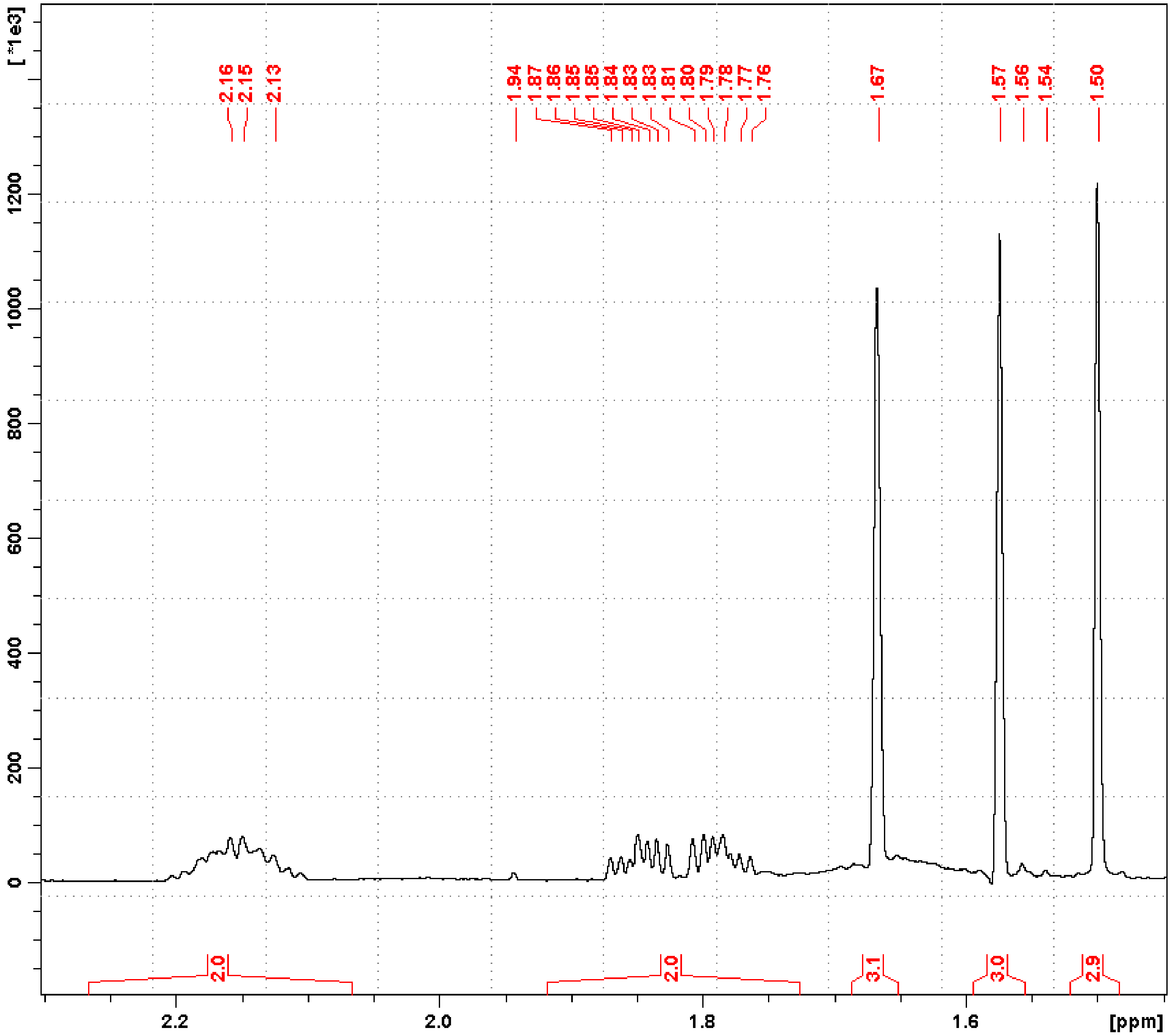

Figure S6. ¹H NMR spectrum of **10** (enlarged).

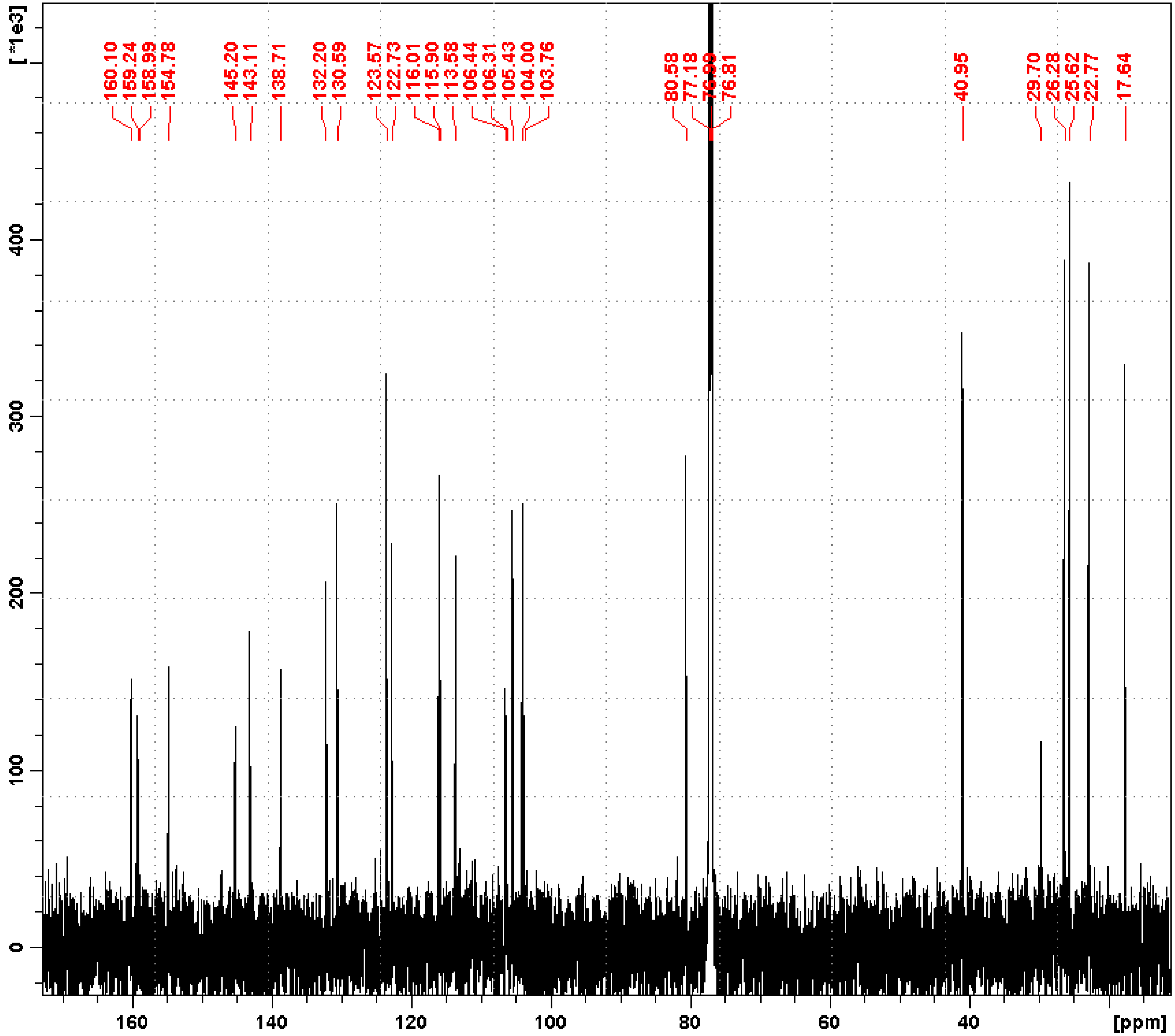

Figure S7. [1]C NMR spectrum of **10**.

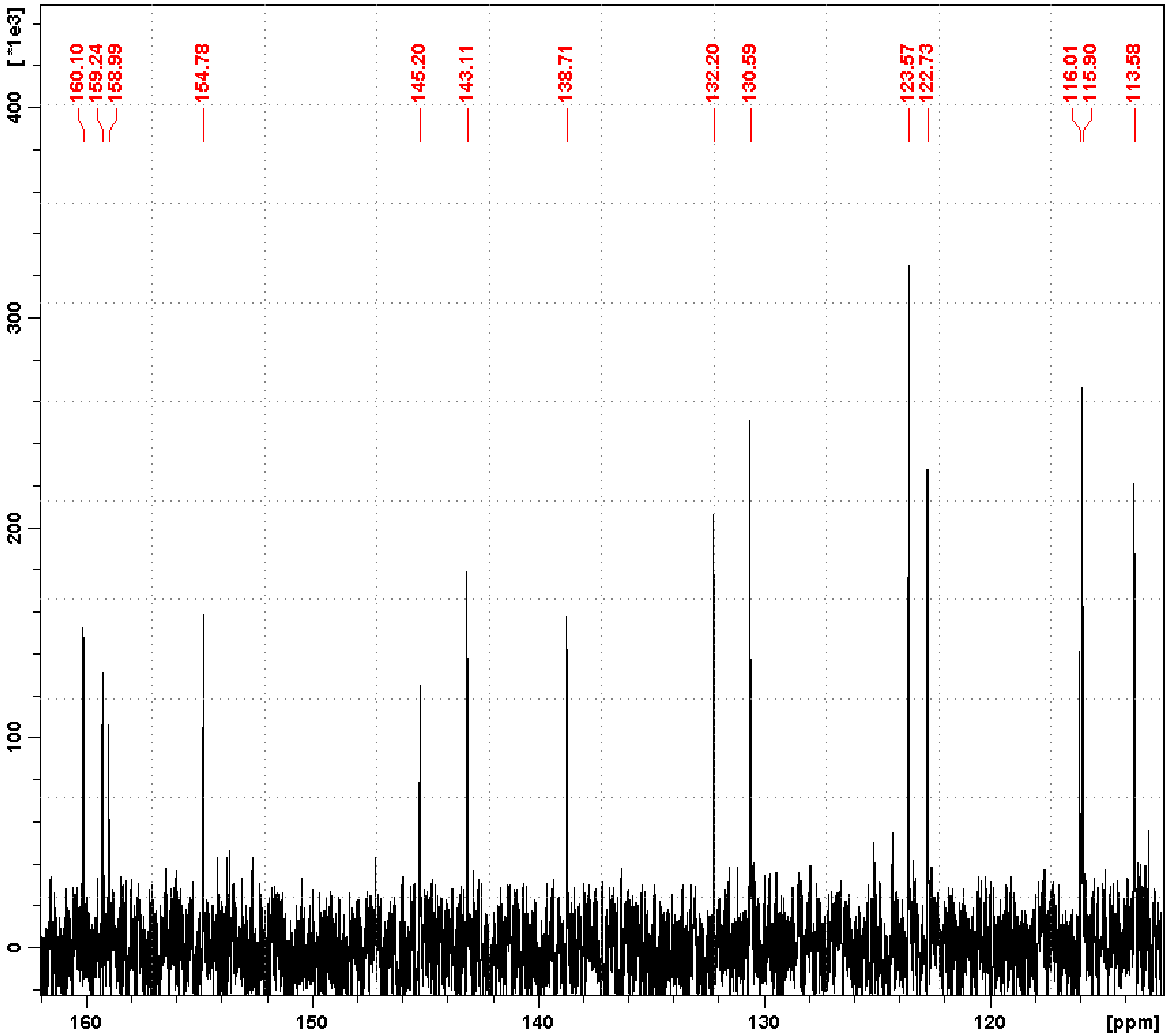

Figure S8. [1]C NMR spectrum of **10** (enlarged).

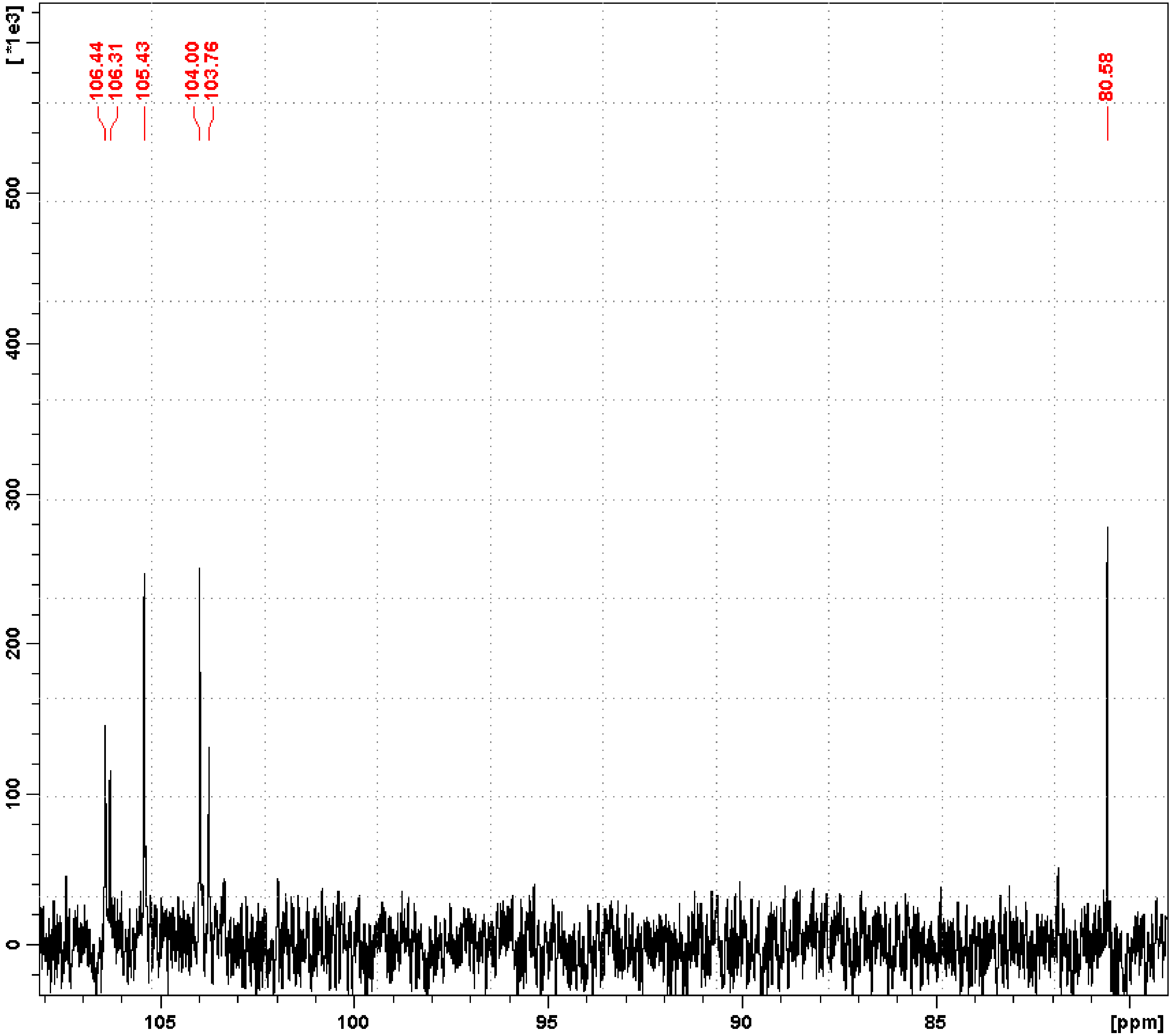

Figure S9. 1C NMR spectrum of **10** (enlarged).

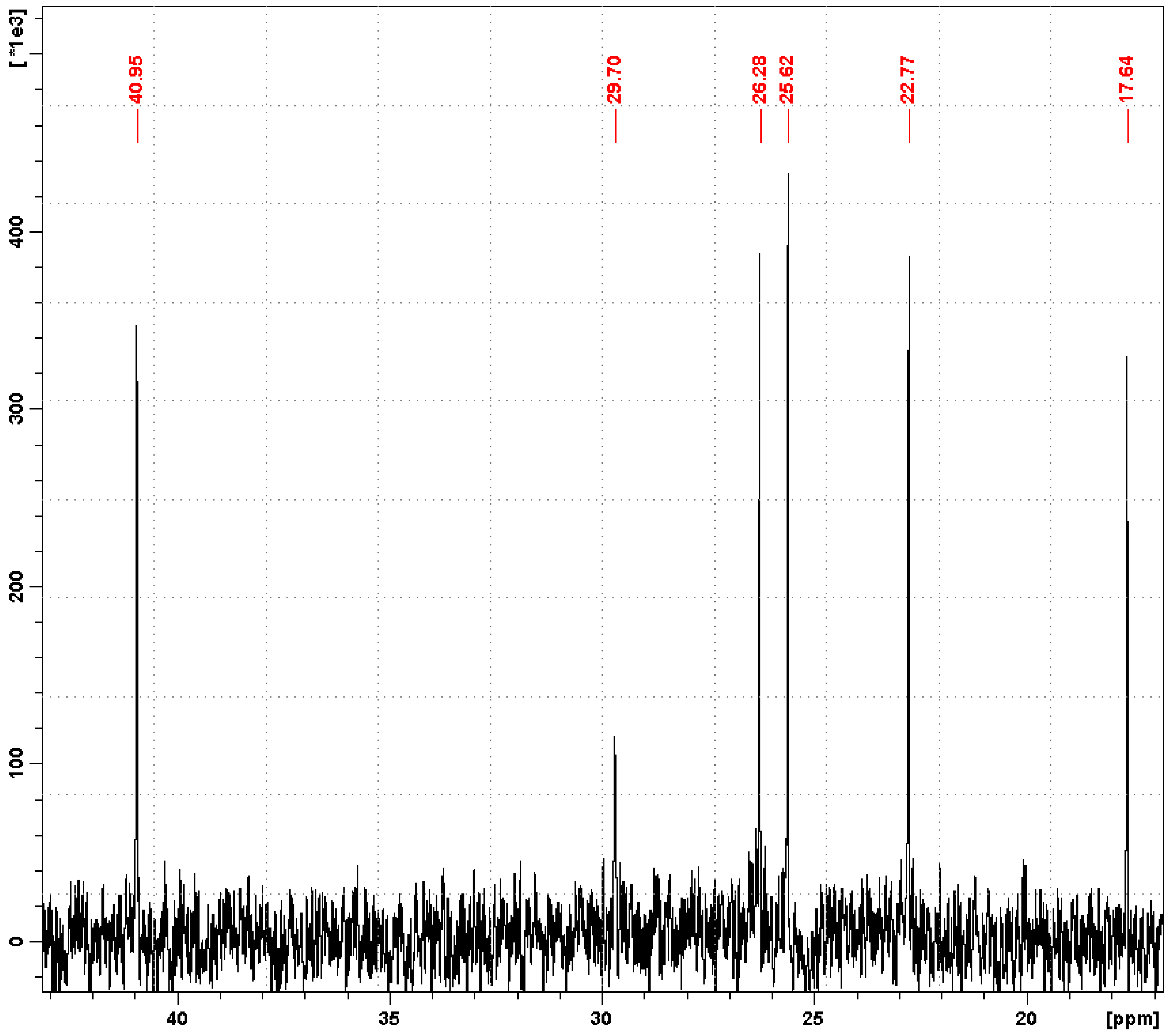

Figure S10. [1]C NMR spectrum of **10** (enlarged).

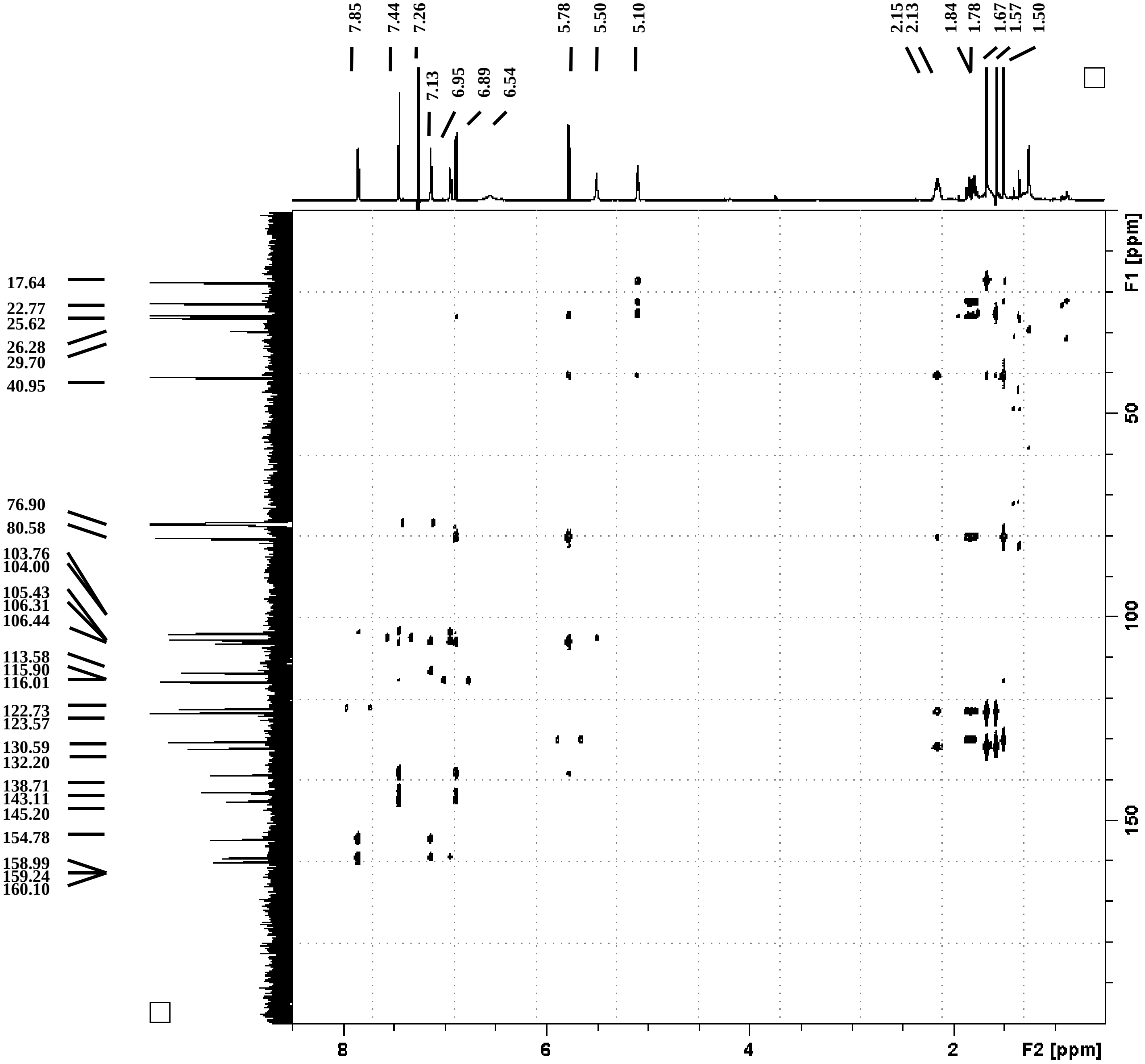

Figure S11. HMBC NMR spectrum of **10**.

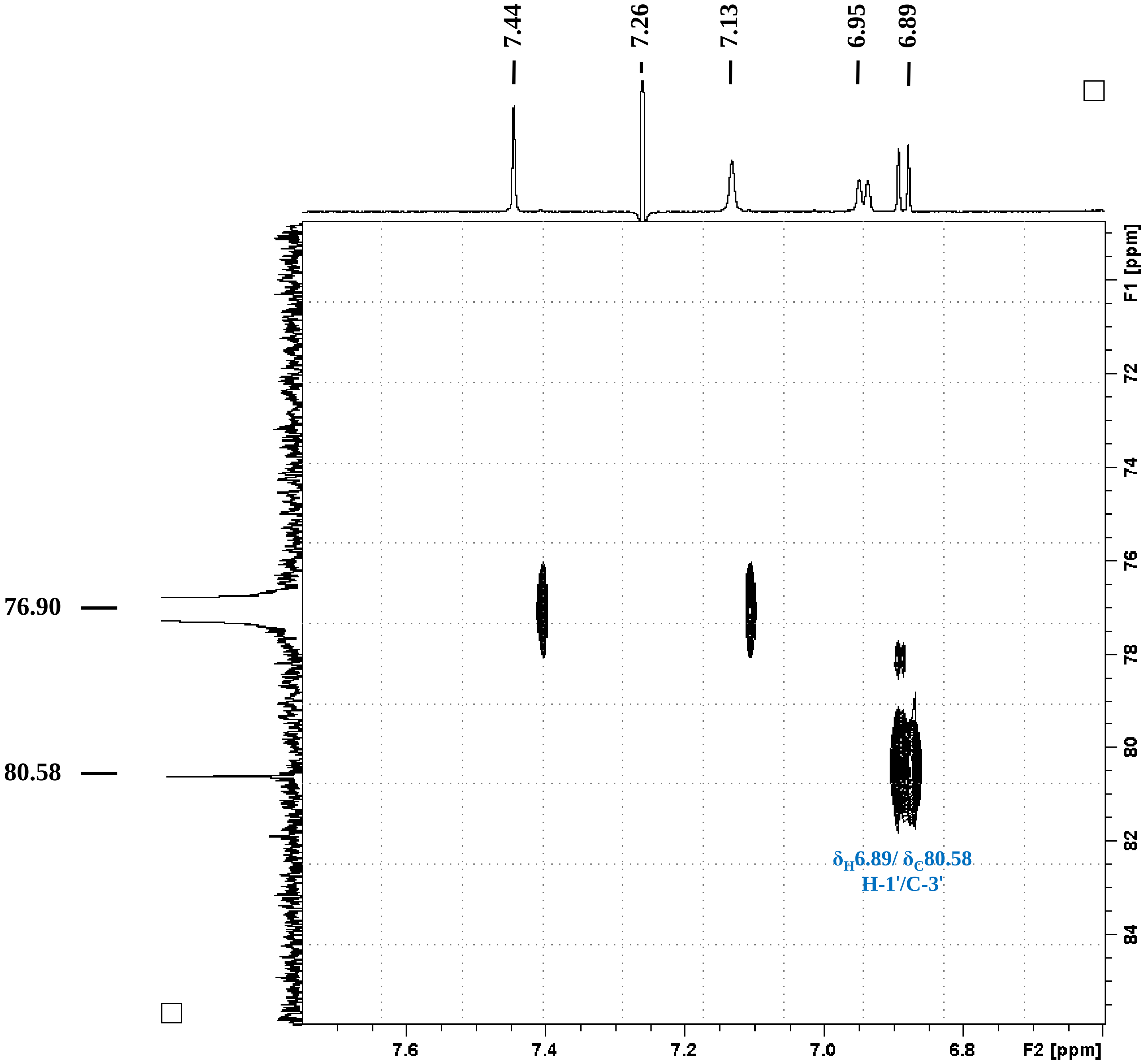

Figure S12. HMBC NMR spectrum of **10** (enlarged).

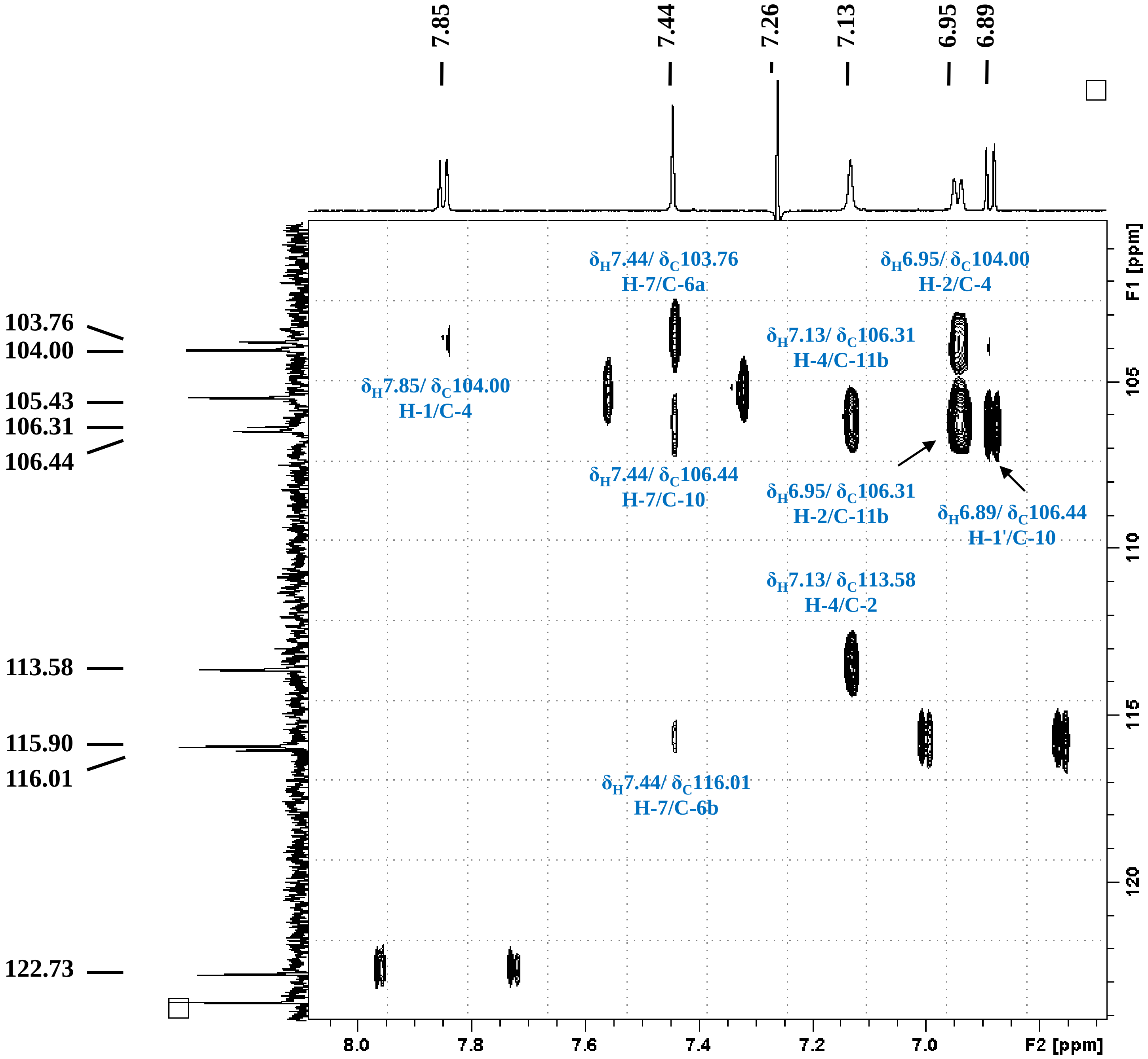

Figure S13. HMBC NMR spectrum of **10** (enlarged).

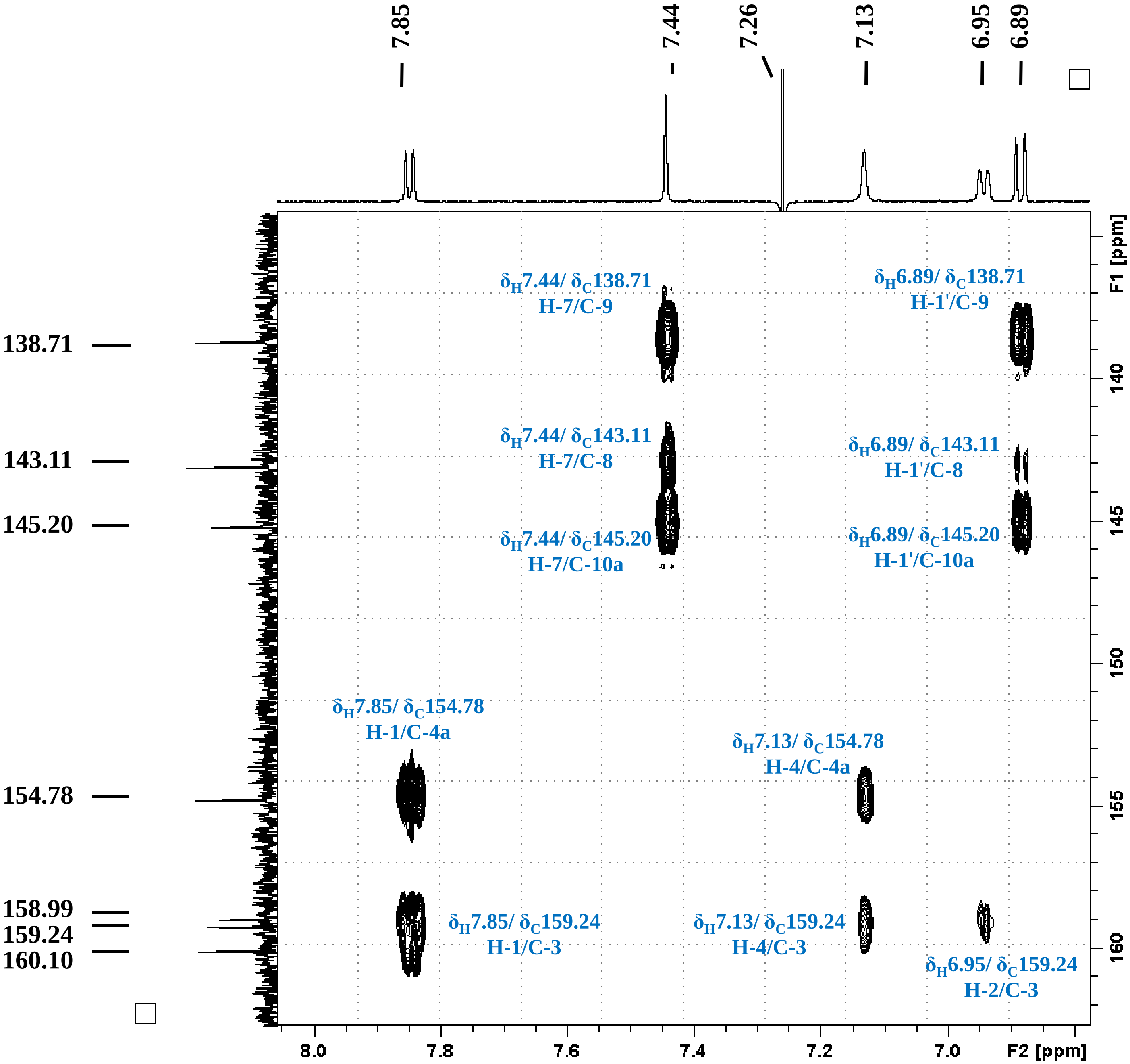

Figure S14. HMBC NMR spectrum of **10** (enlarged).

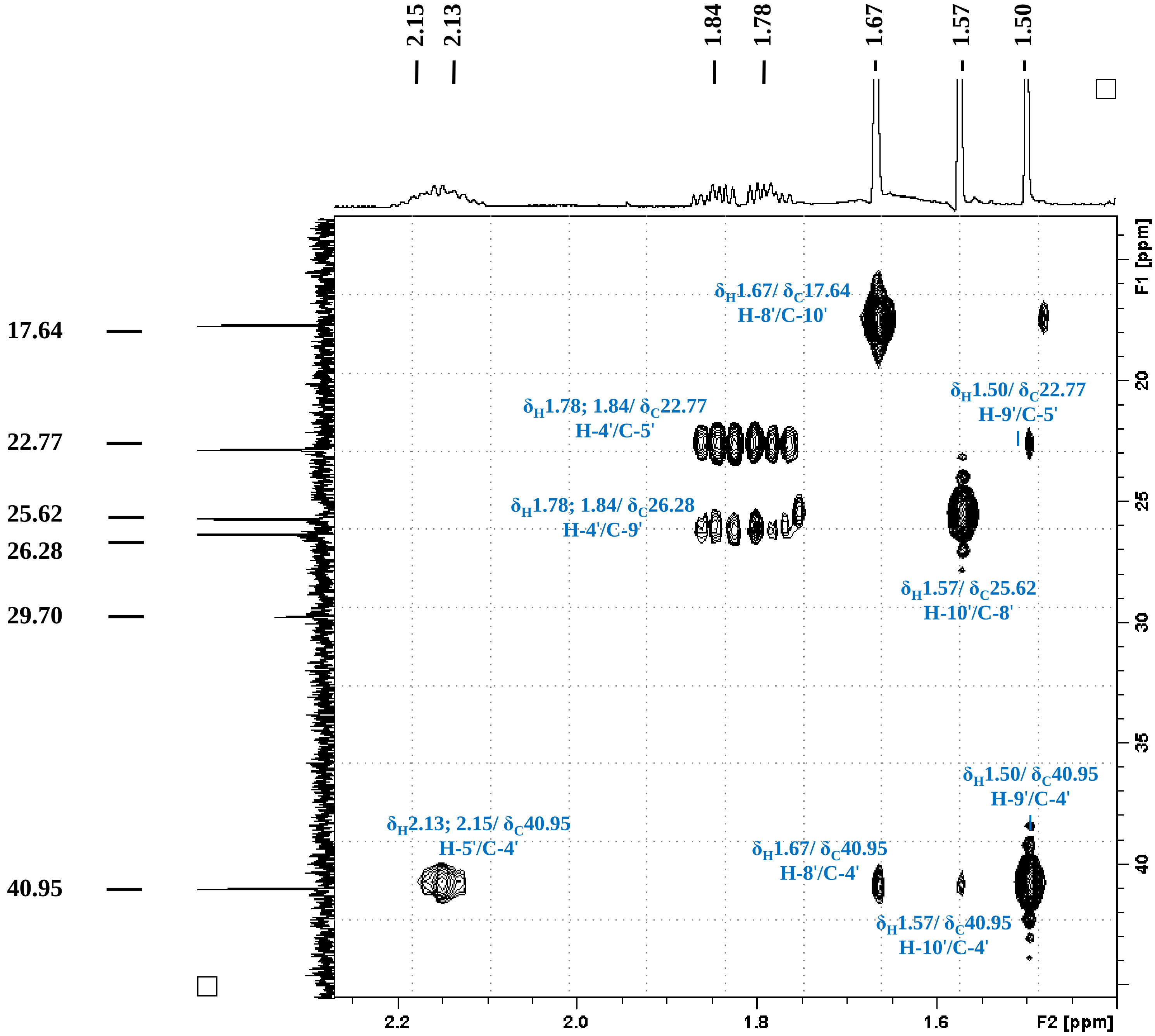

Figure S15. HMBC NMR spectrum of **10** (enlarged).

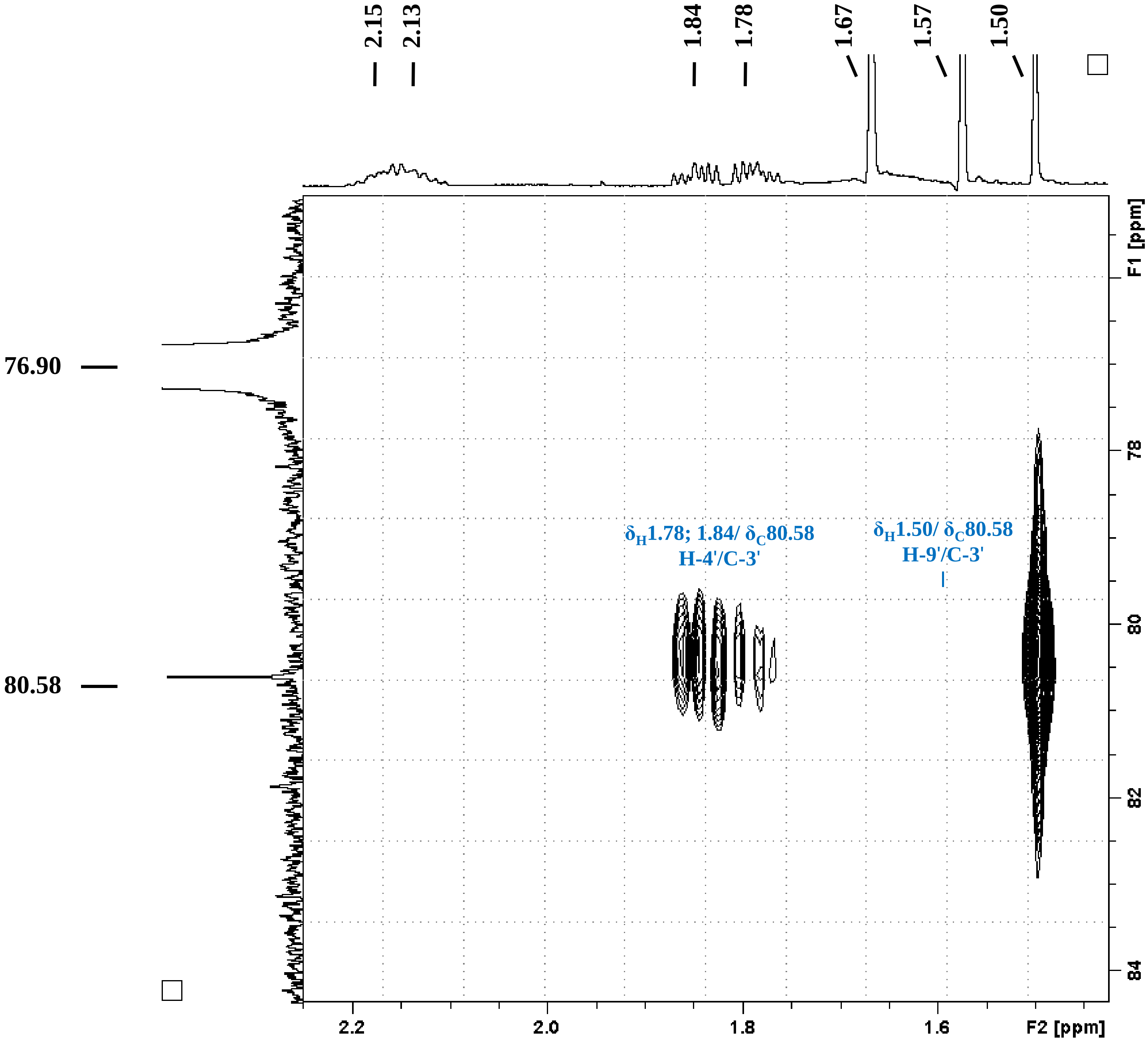

Figure S16. HMBC NMR spectrum of **10** (enlarged).

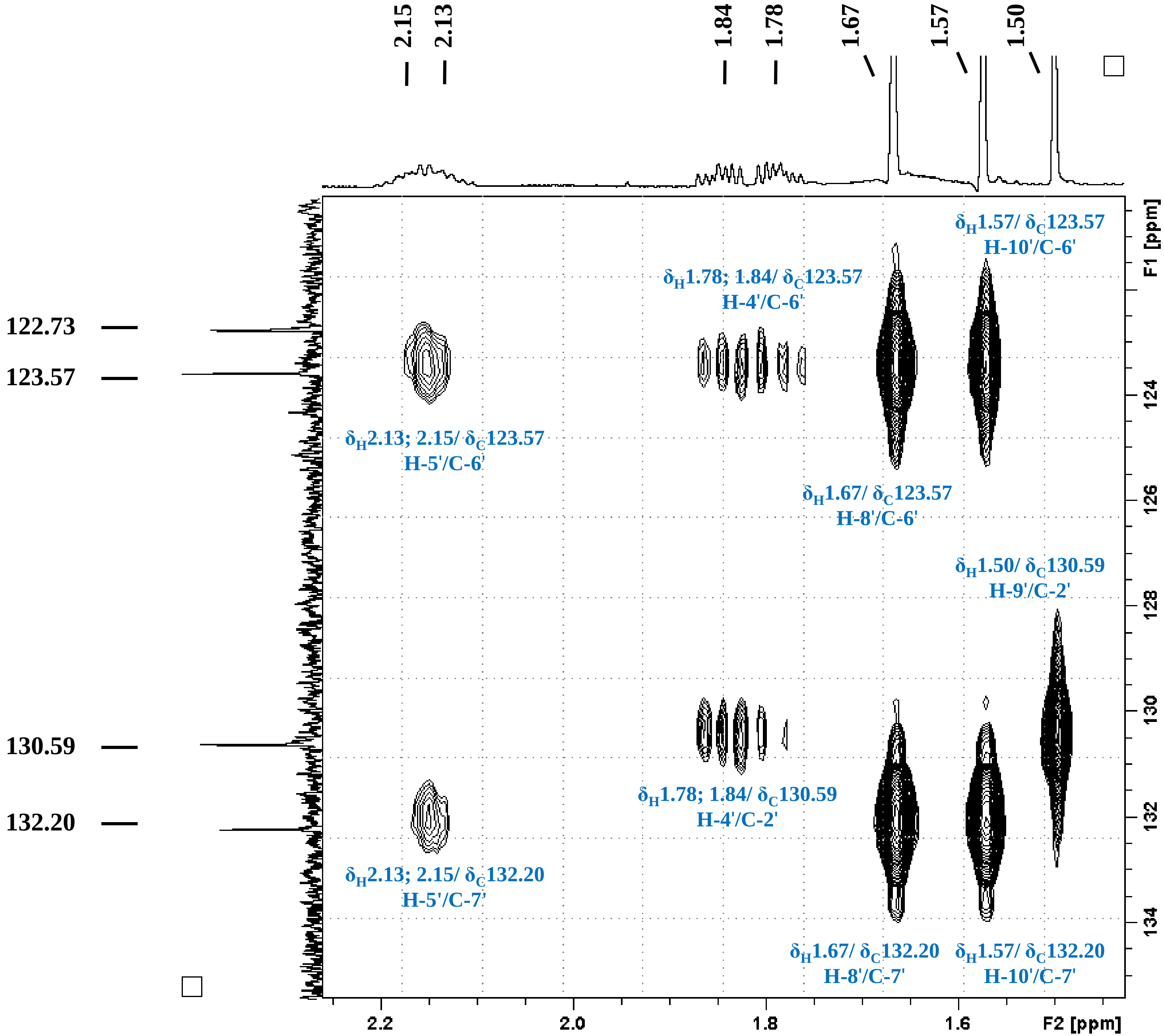

Figure S17. HMBC NMR spectrum of **10** (enlarged).

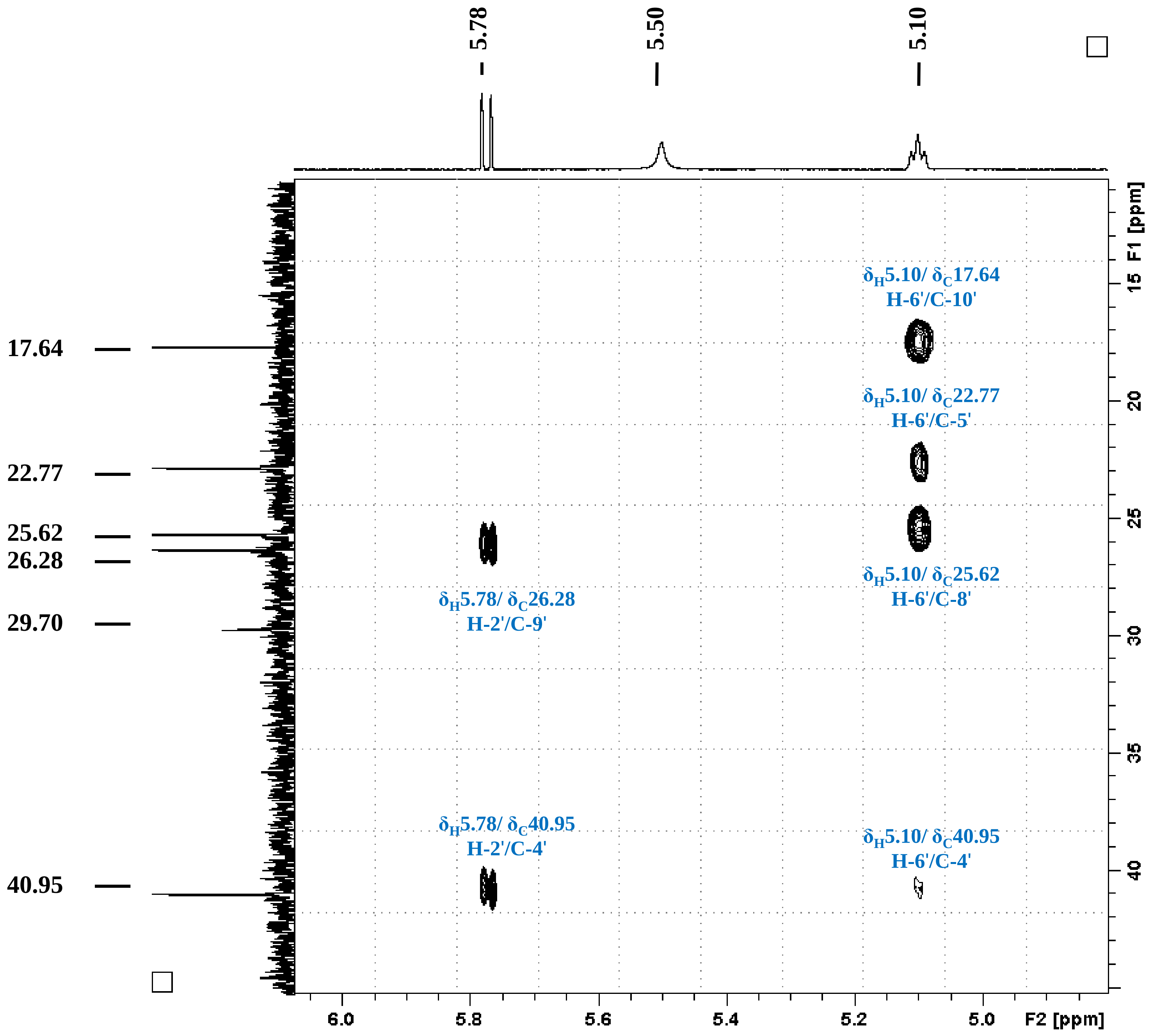

Figure S18. HMBC NMR spectrum of **10** (enlarged).

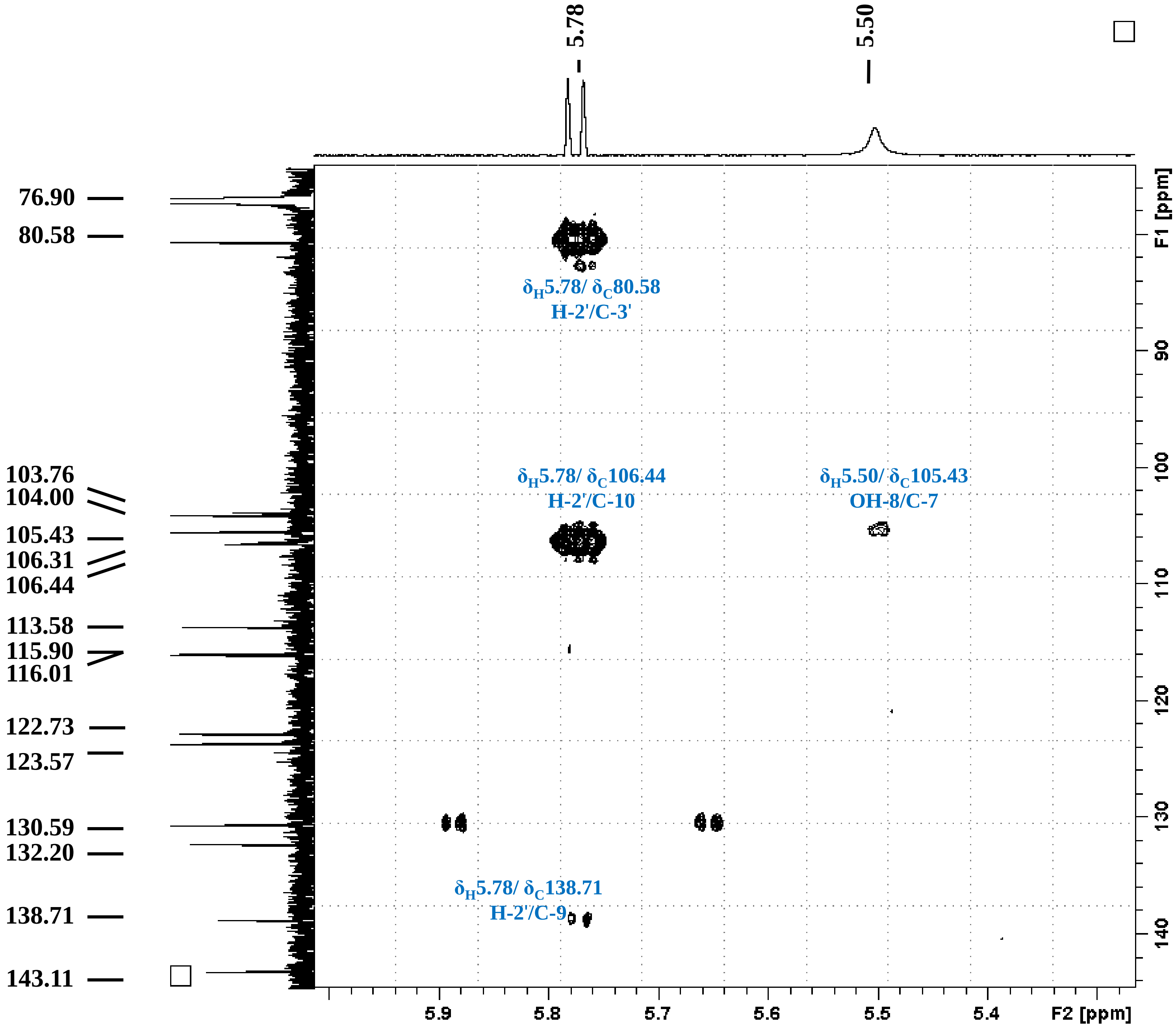

Figure S19. HMBC NMR spectrum of **10** (enlarged).

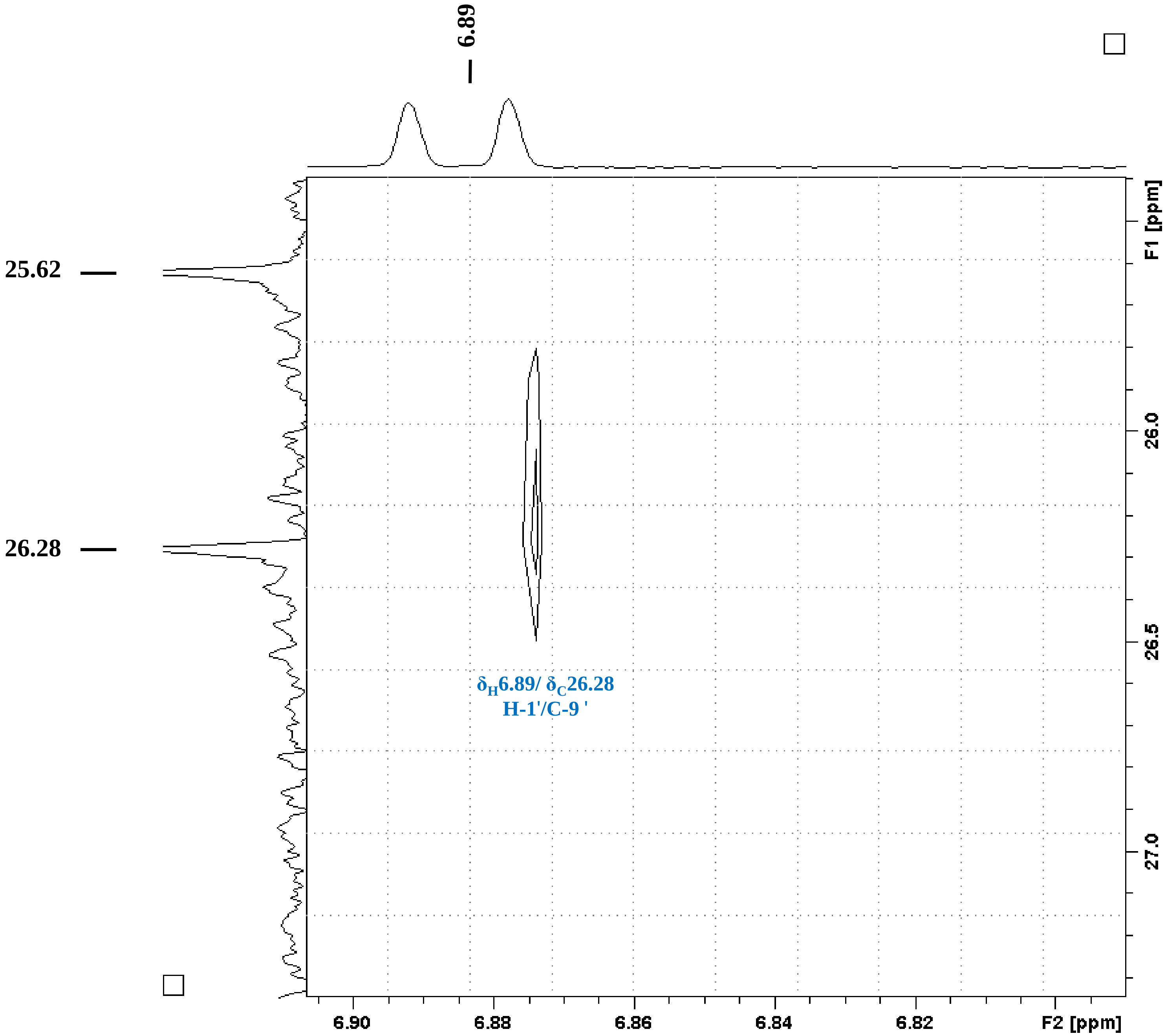

Figure S20. HMBC NMR spectrum of **10** (enlarged).

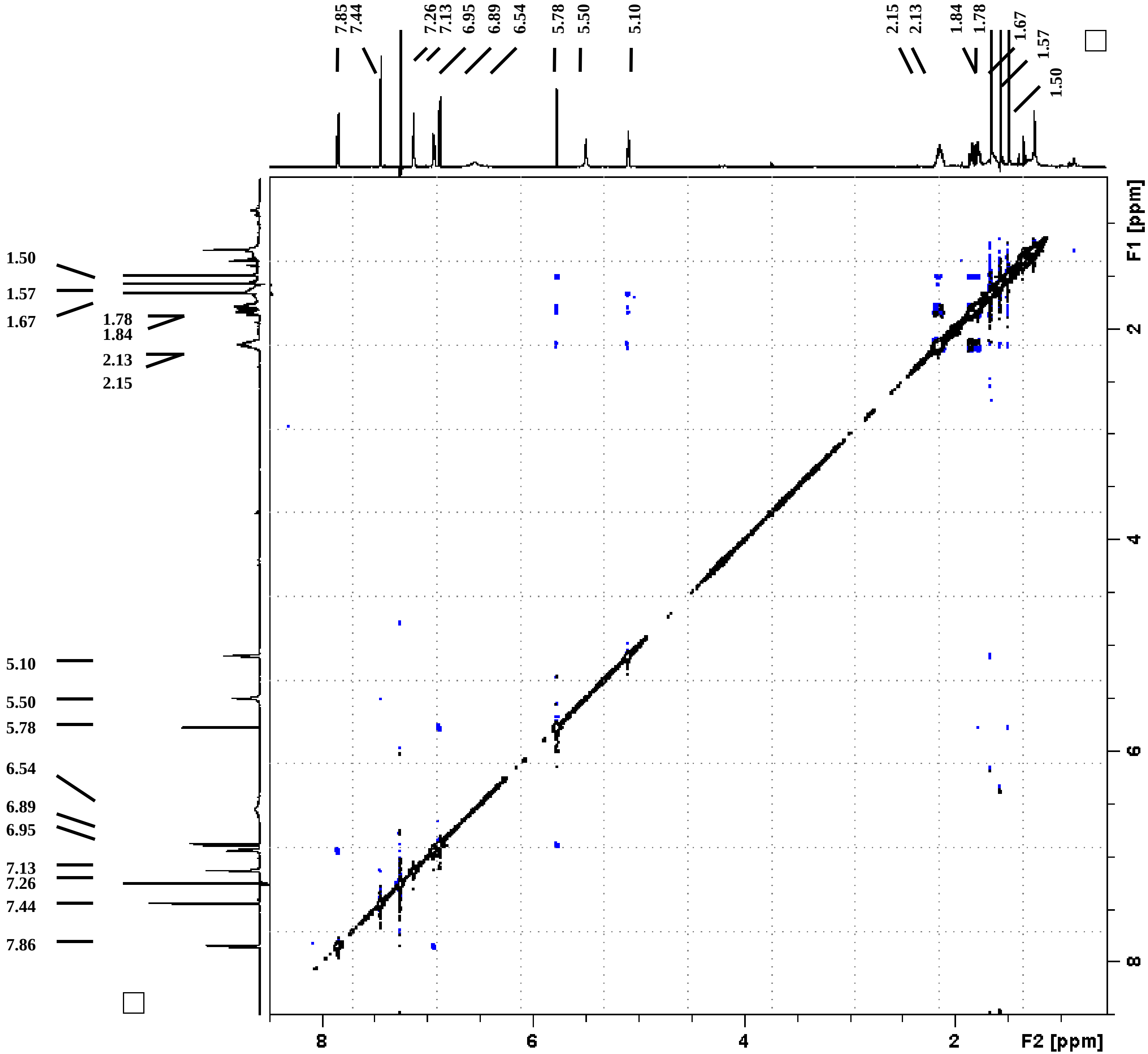

Figure S21. ROESY NMR spectrum of **10**.

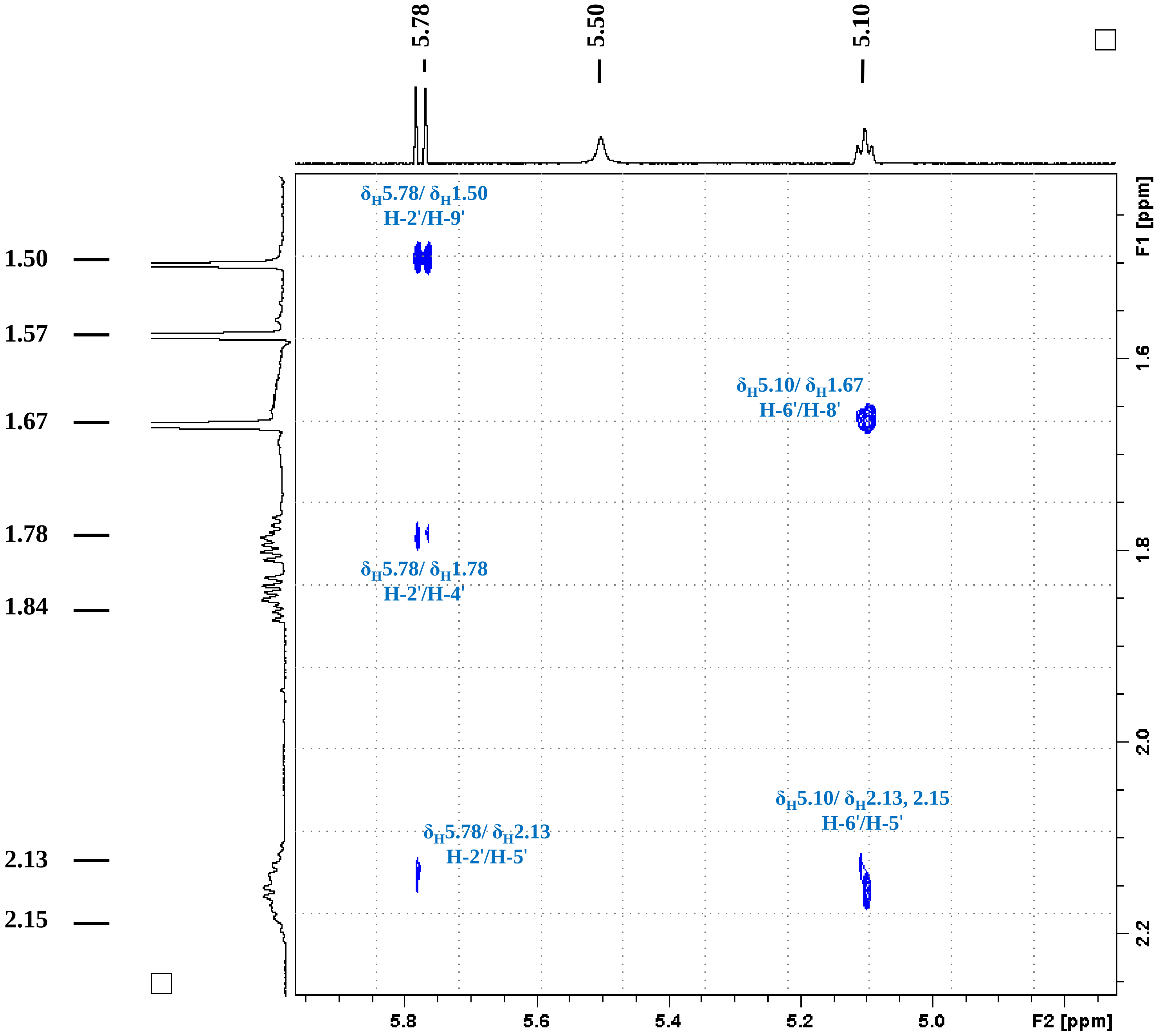

Figure S22. ROESY NMR spectrum of **10** (enlarged).

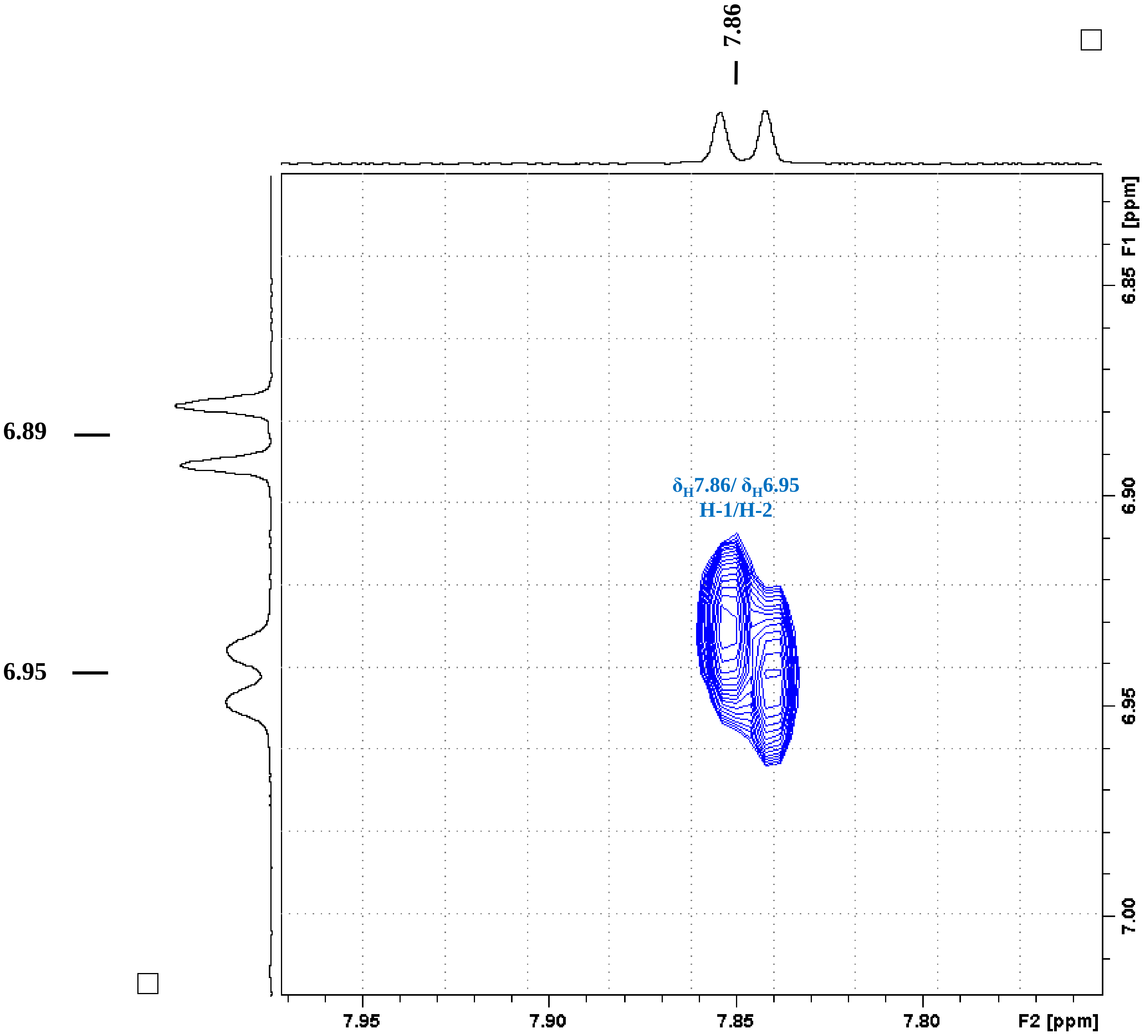

Figure S23. ROESY NMR spectrum of **10** (enlarged).

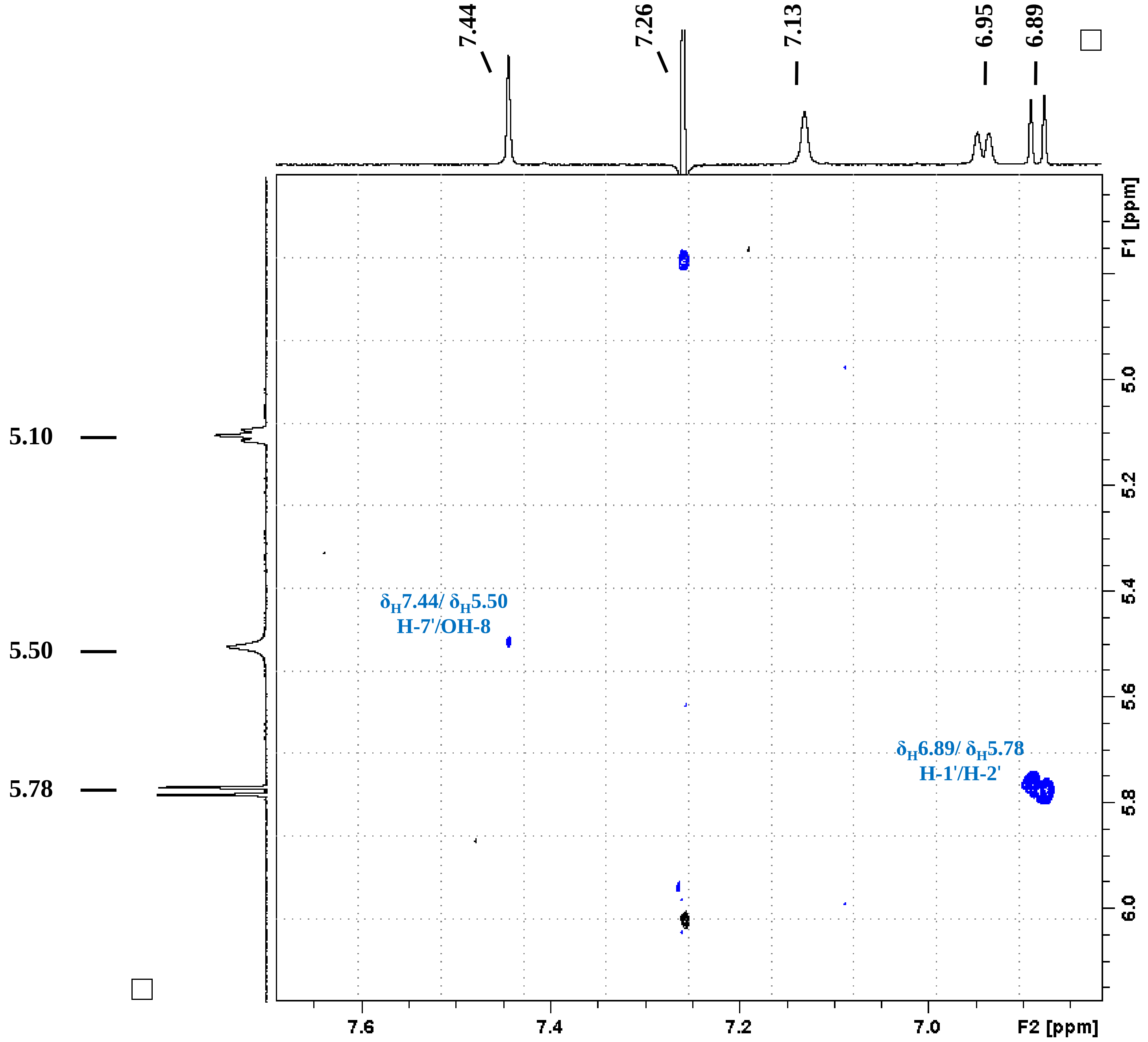

Figure S24. ROESY NMR spectrum of **10** (enlarged).

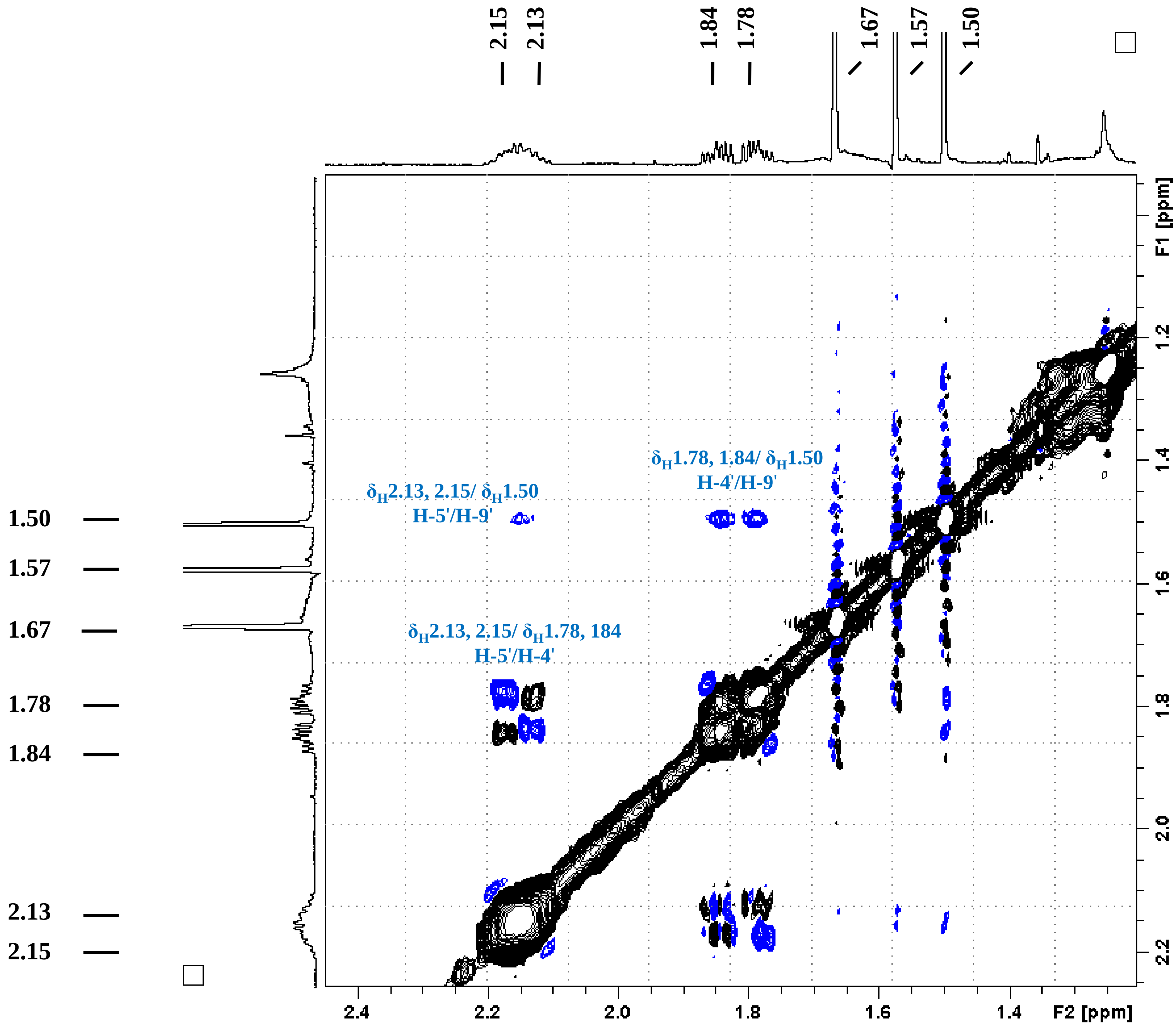

Figure S25. ROESY NMR spectrum of **10** (enlarged).

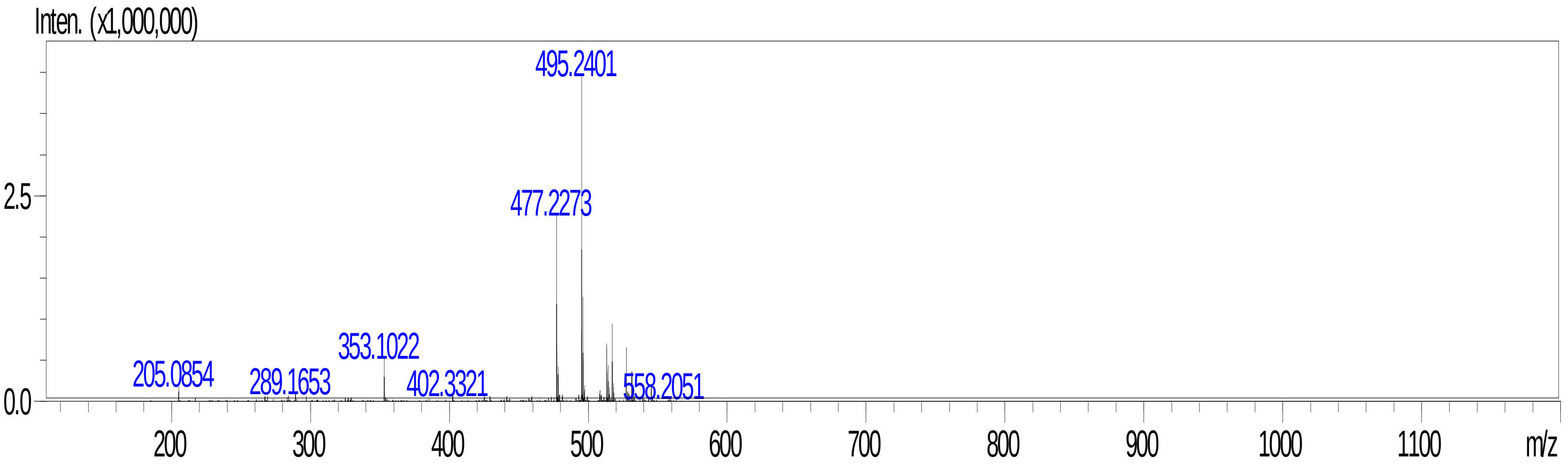

Figure S26. HR-ESI mass-spectrum of **11** $[M+H]^+$.

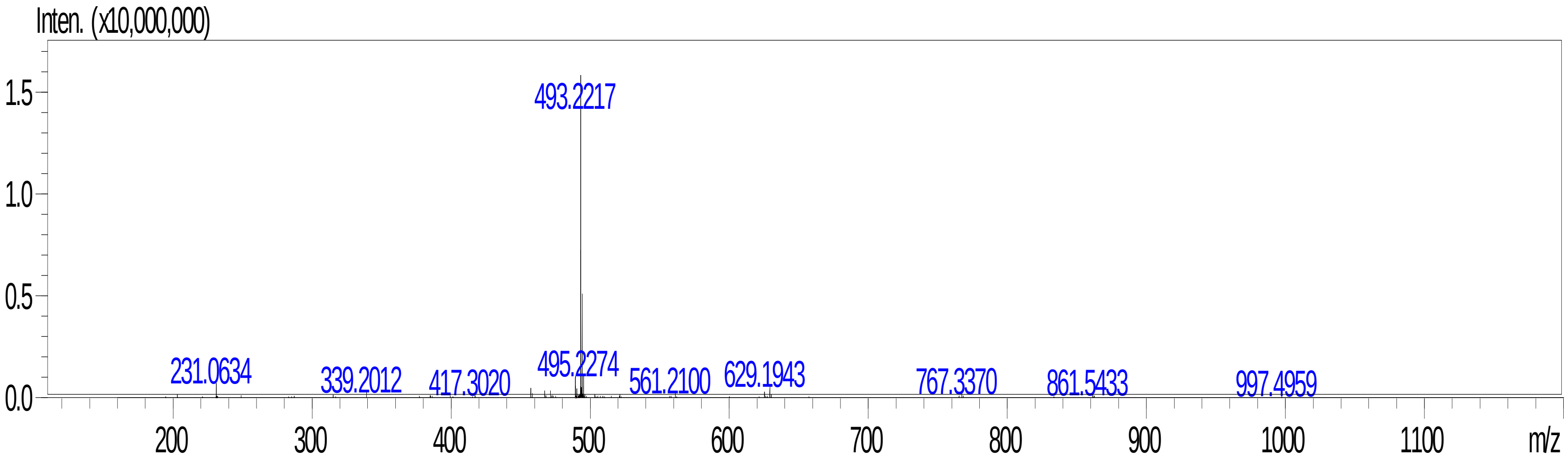

Figure S27. HR-ESI mass-spectrum of **11** [M-H]$^-$.

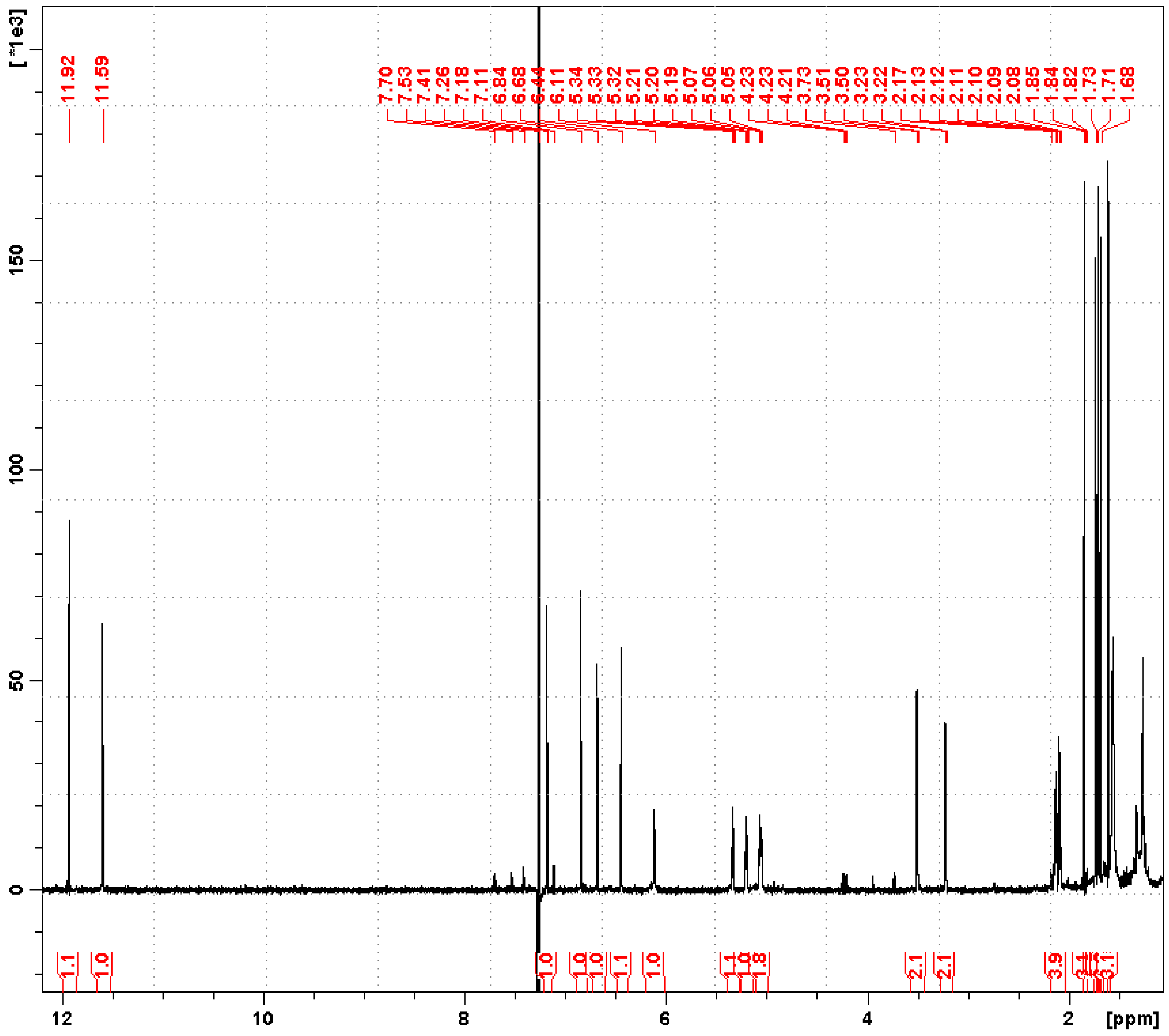

Figure S28. ^{1}H NMR spectrum of **11**.

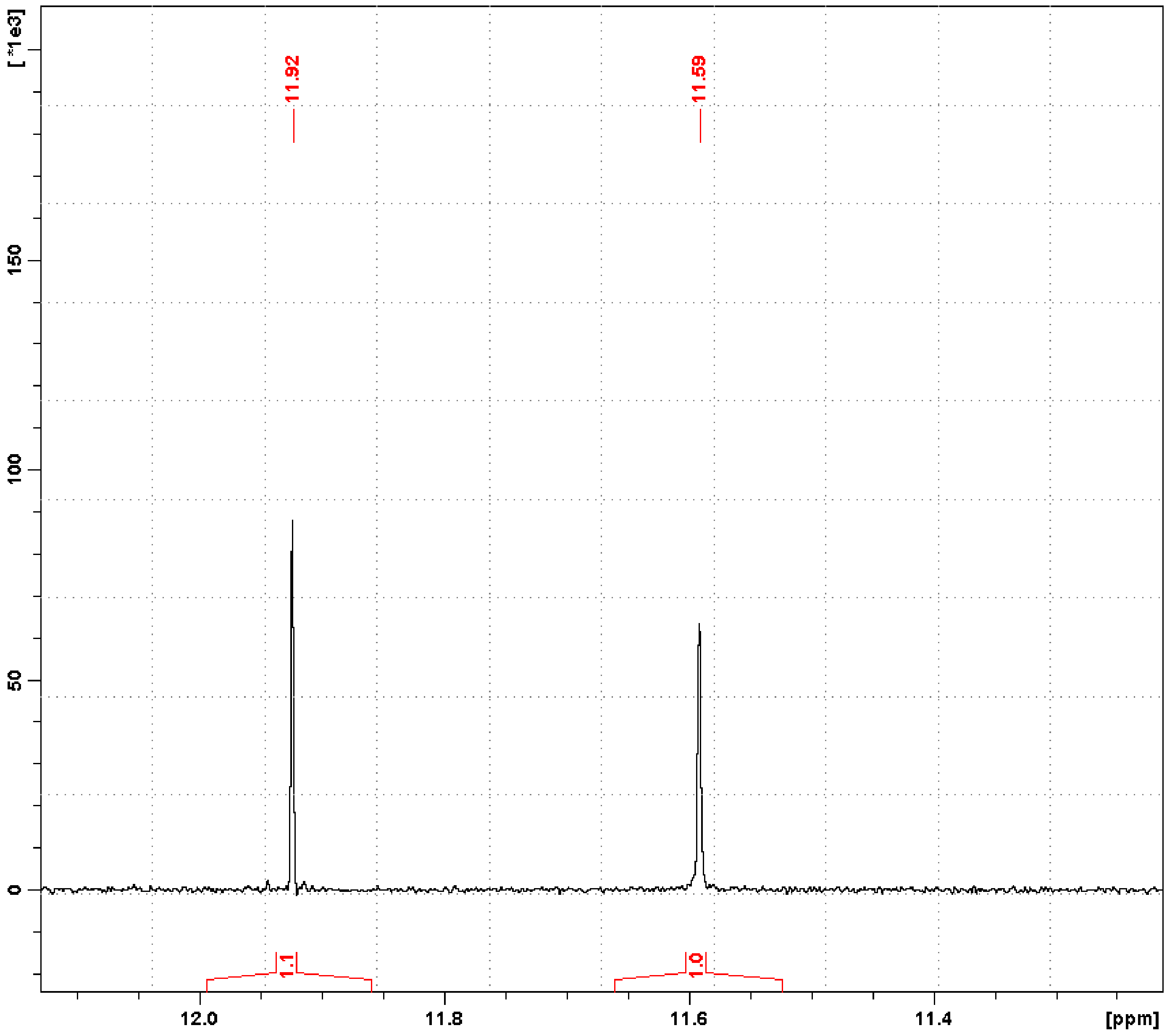

Figure S29. ^{1}H NMR spectrum of **11** (enlarged).

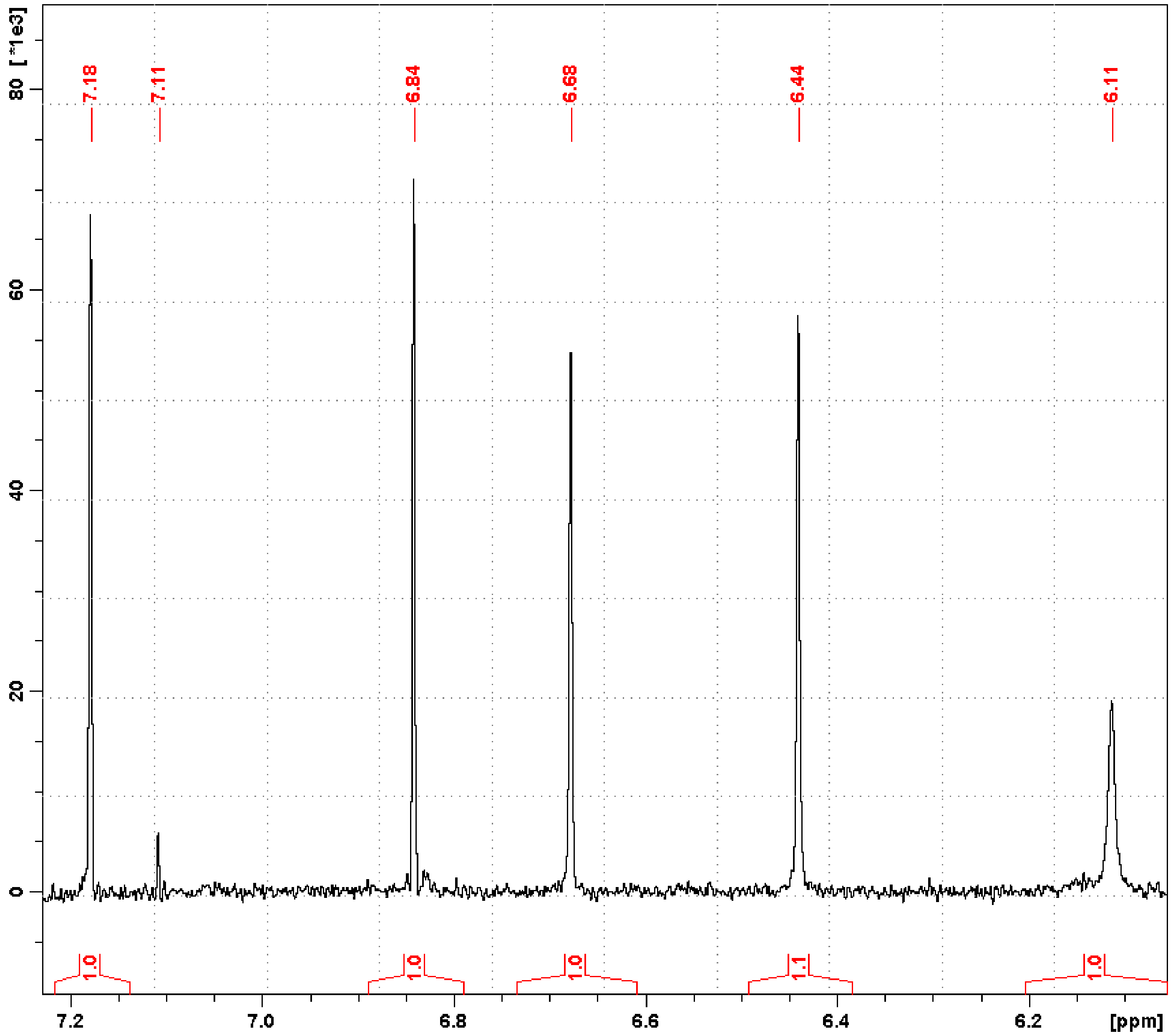

Figure S30. ^{1}H NMR spectrum of **11** (enlarged).

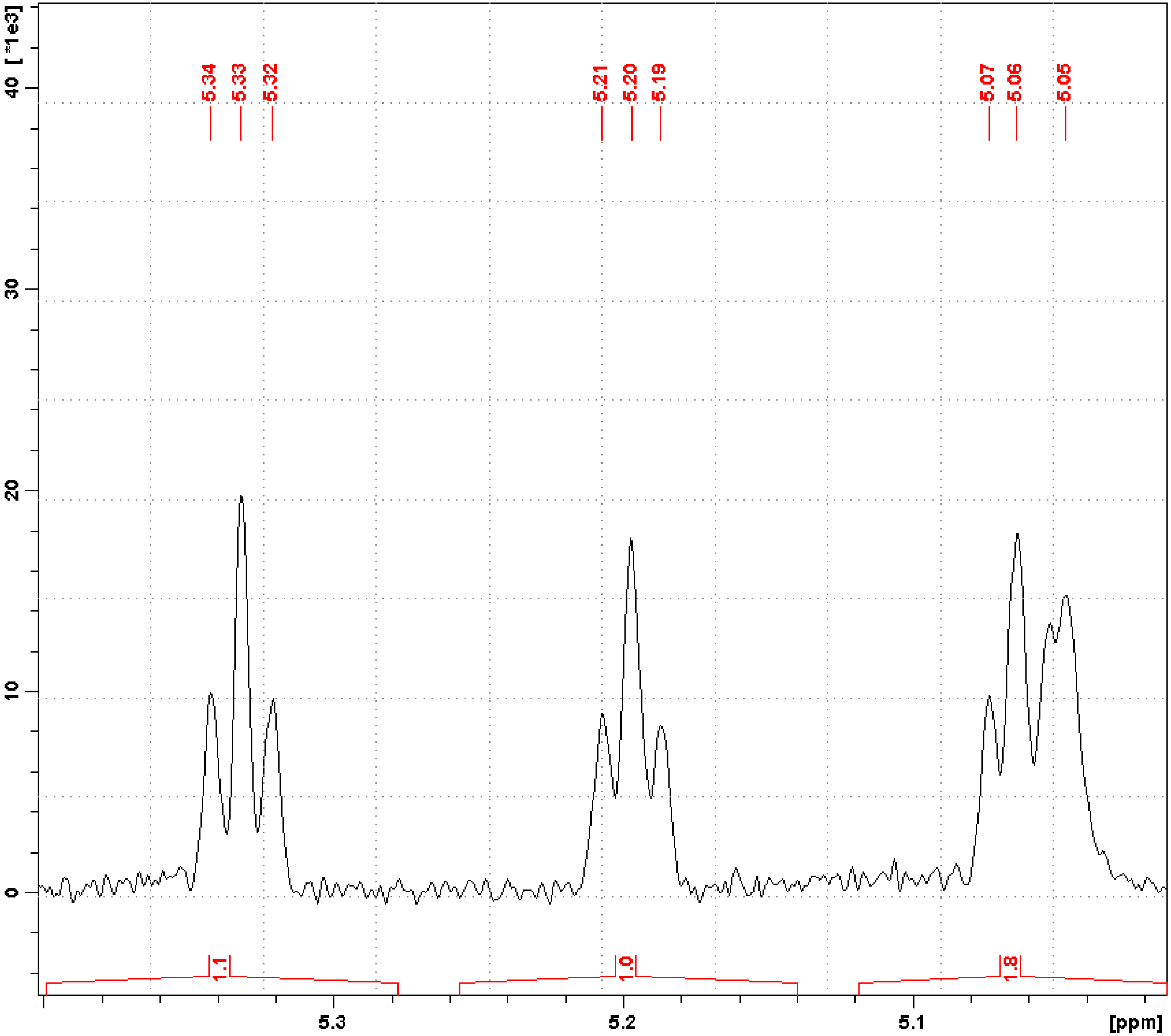

Figure S31. ^{1}H NMR spectrum of **11** (enlarged).

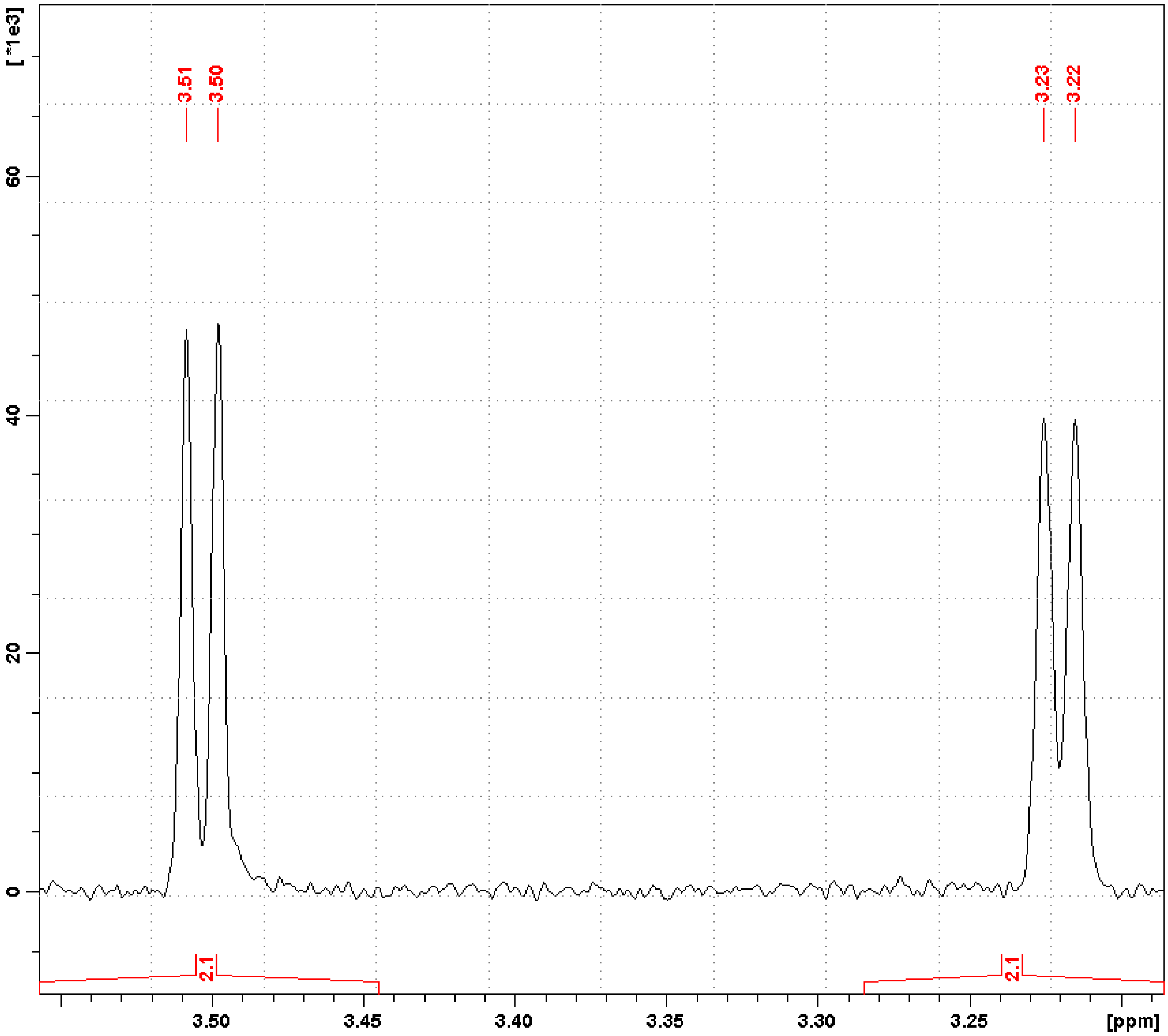

Figure S32. ^{1}H NMR spectrum of **11** (enlarged).

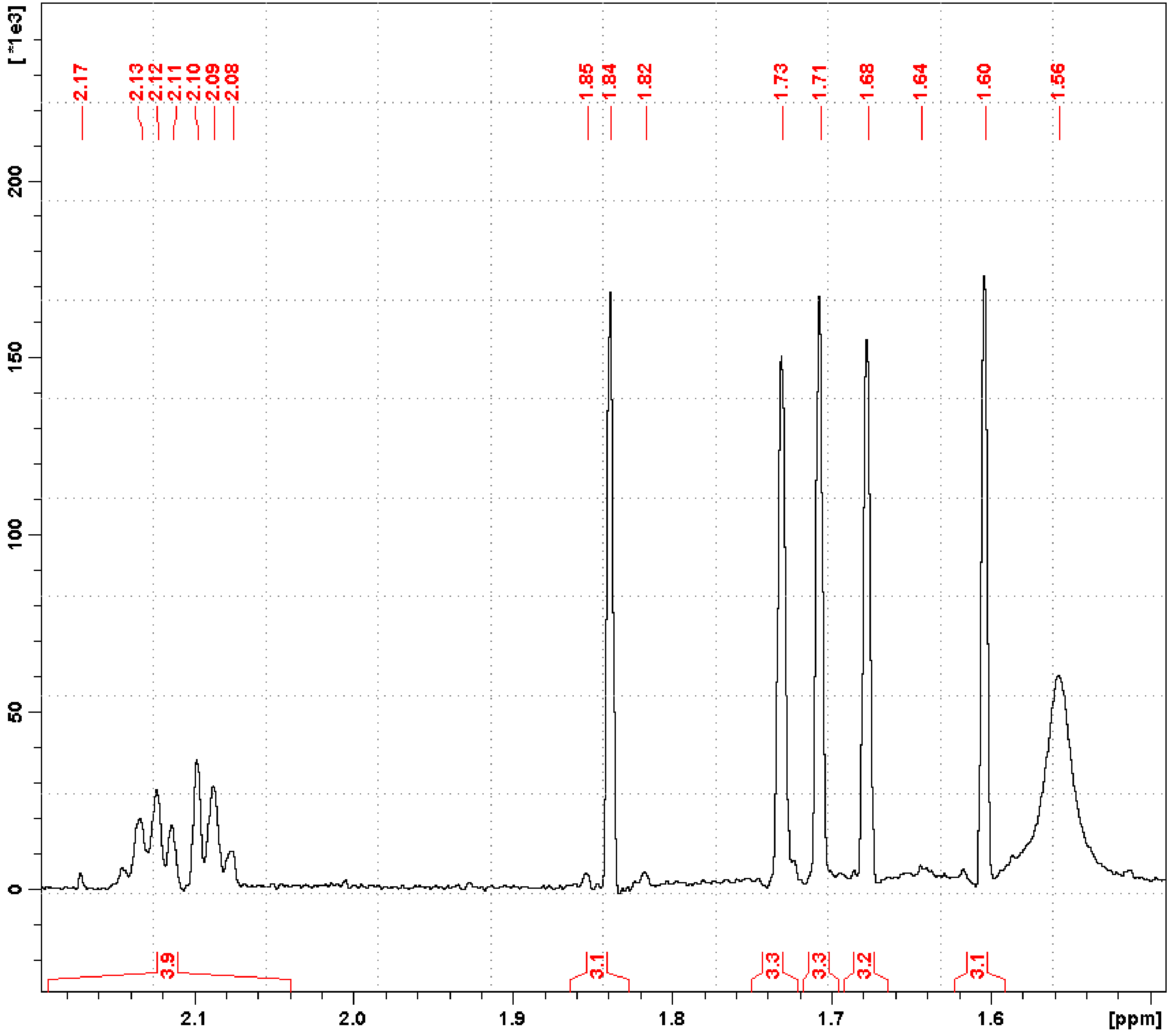

Figure S33. ^{1}H NMR spectrum of **11** (enlarged).

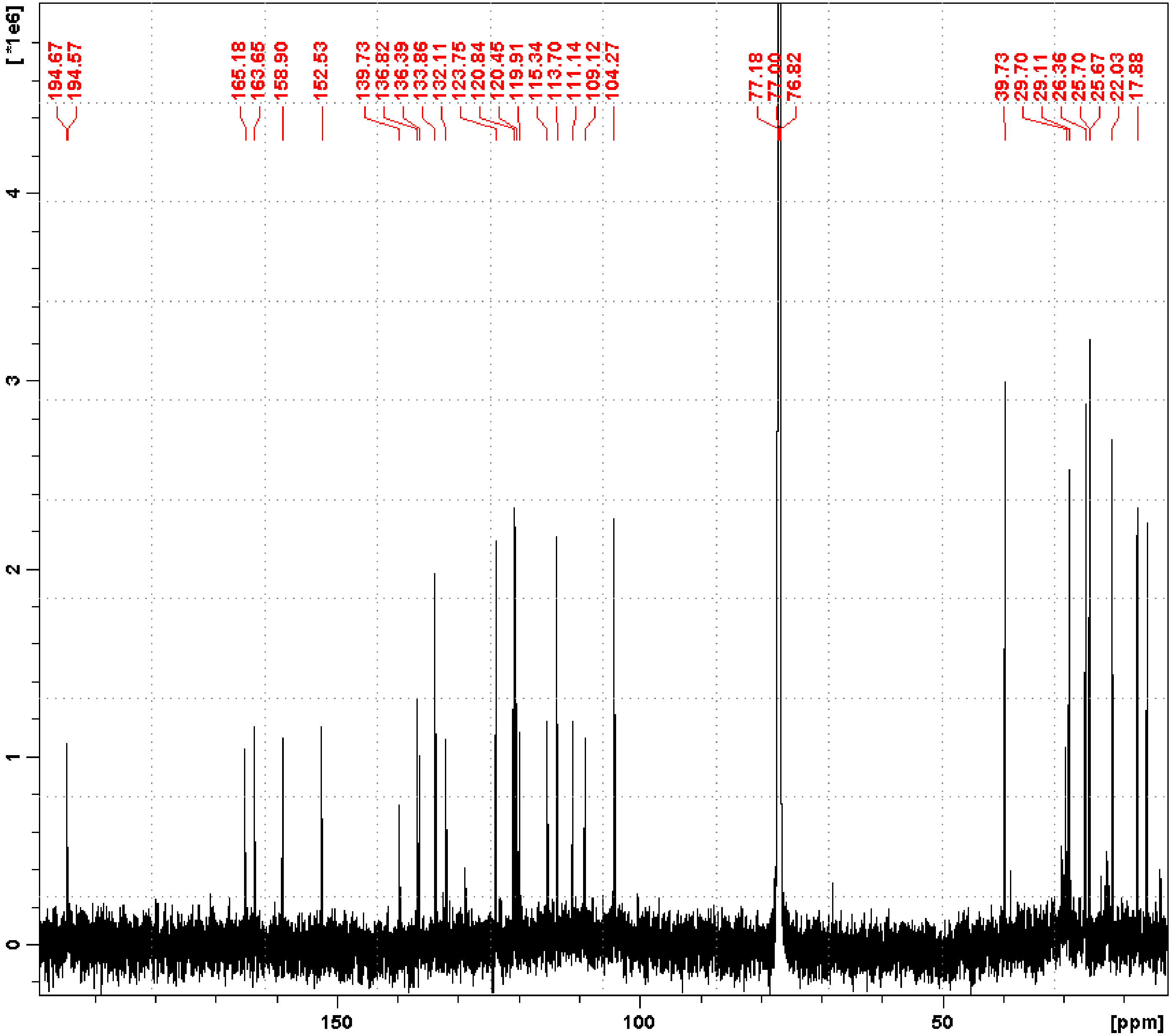

Figure S34. ^{13}C NMR spectrum of **11**.

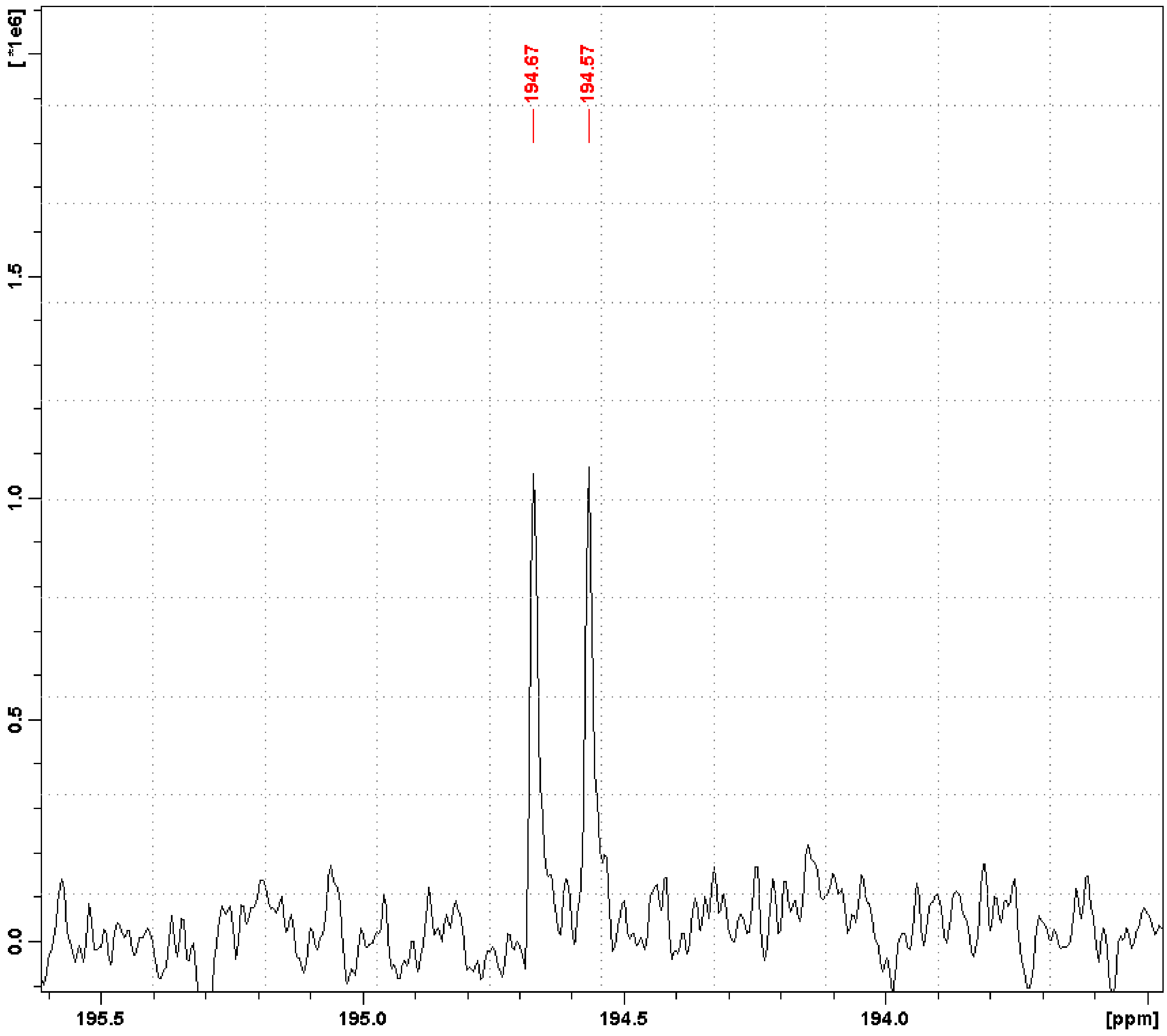

Figure S35. ^{13}C NMR spectrum of **11** (enlarged).

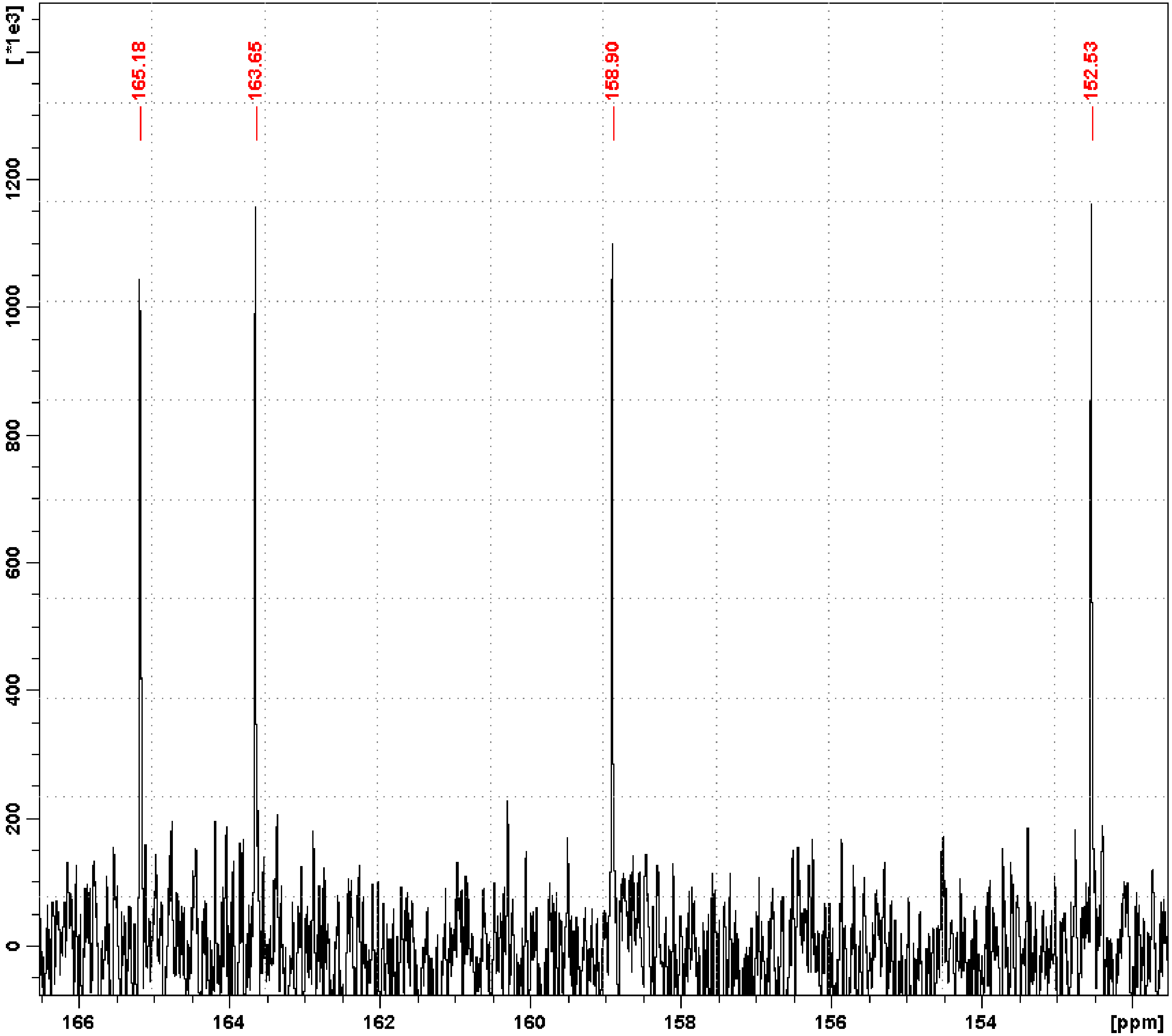

Figure S36. ^{13}C NMR spectrum of **11** (enlarged).

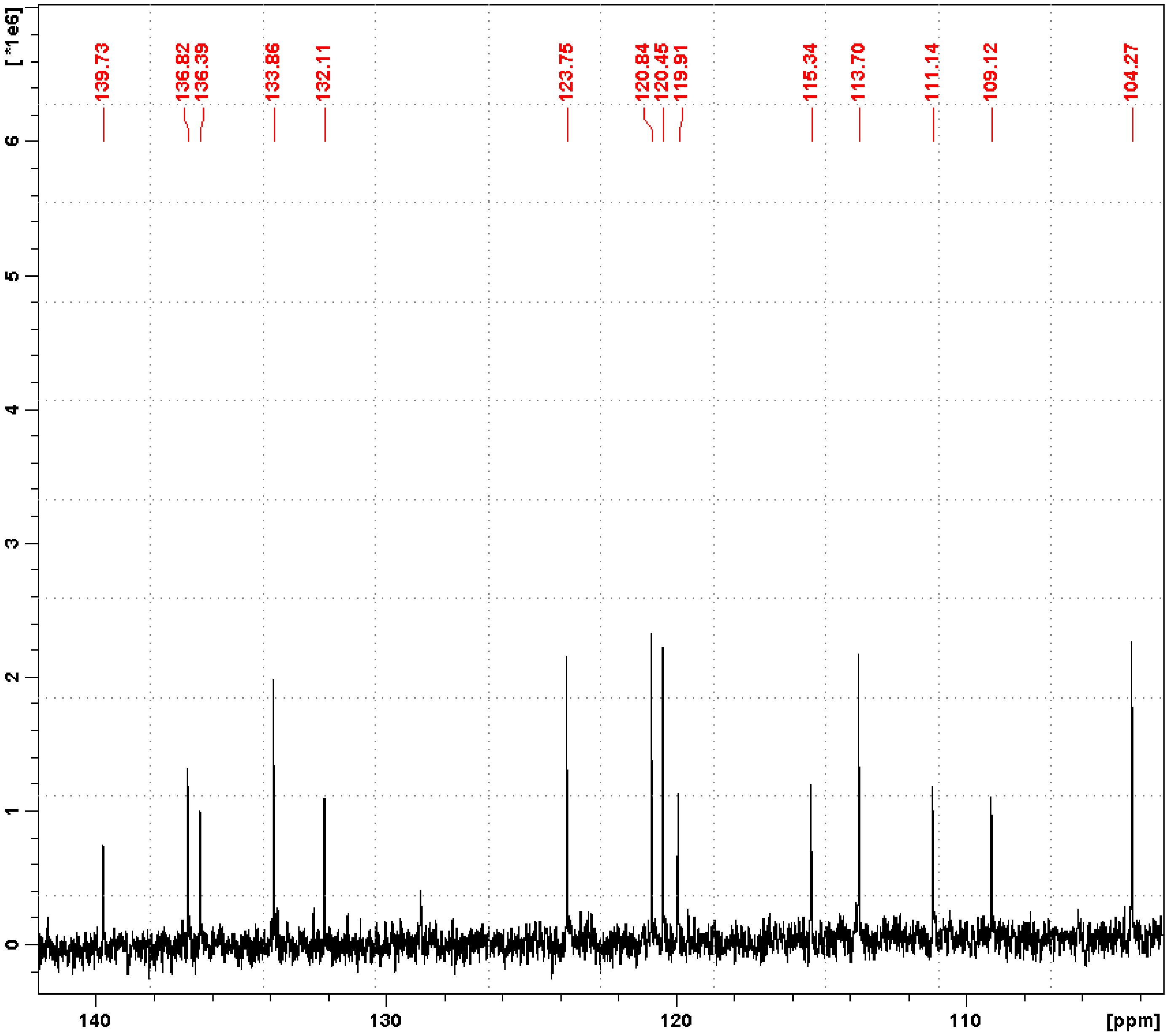

Figure S37. ^{13}C NMR spectrum of **11** (enlarged).

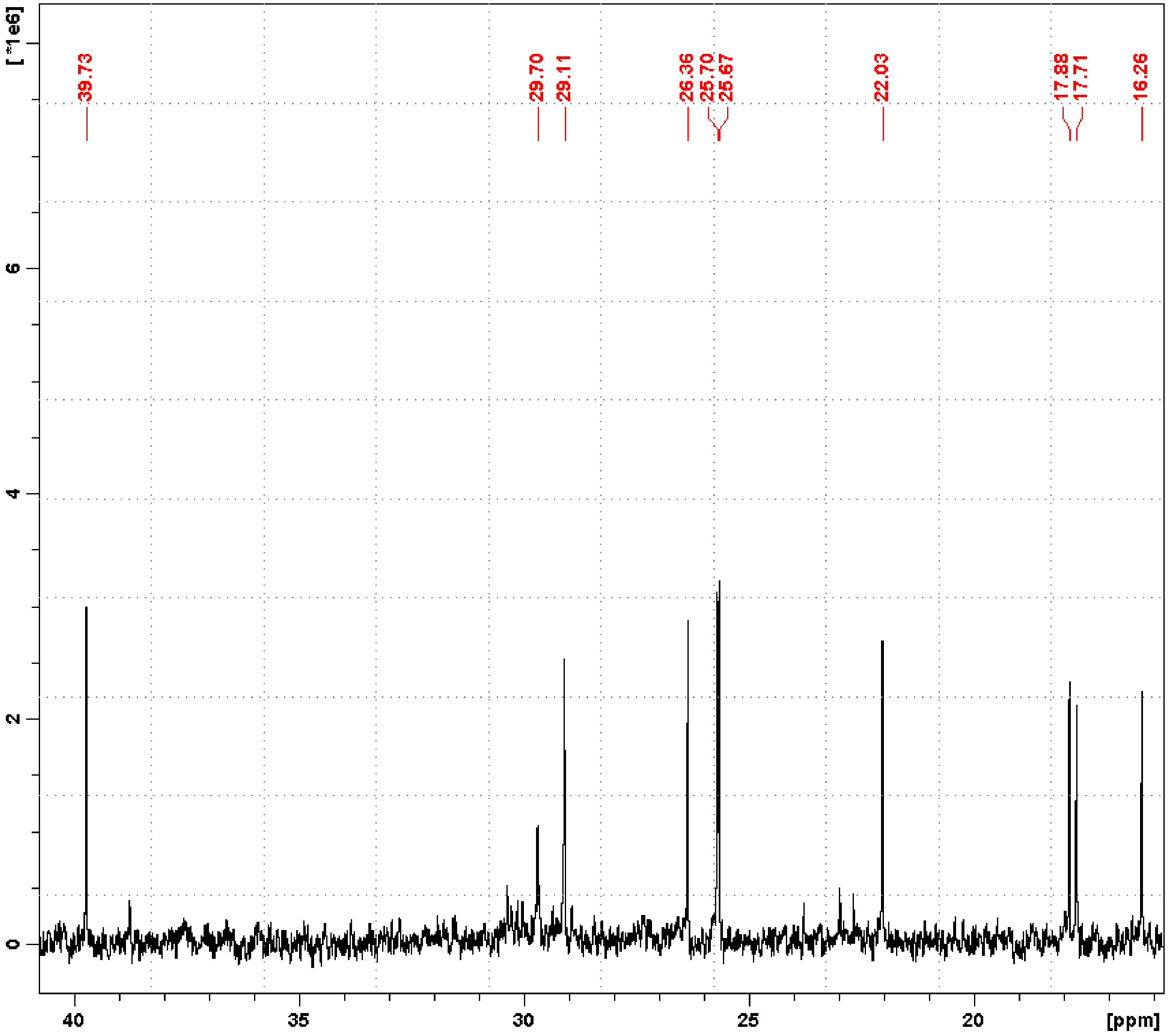

Figure S38. ^{13}C NMR spectrum of **11** (enlarged).

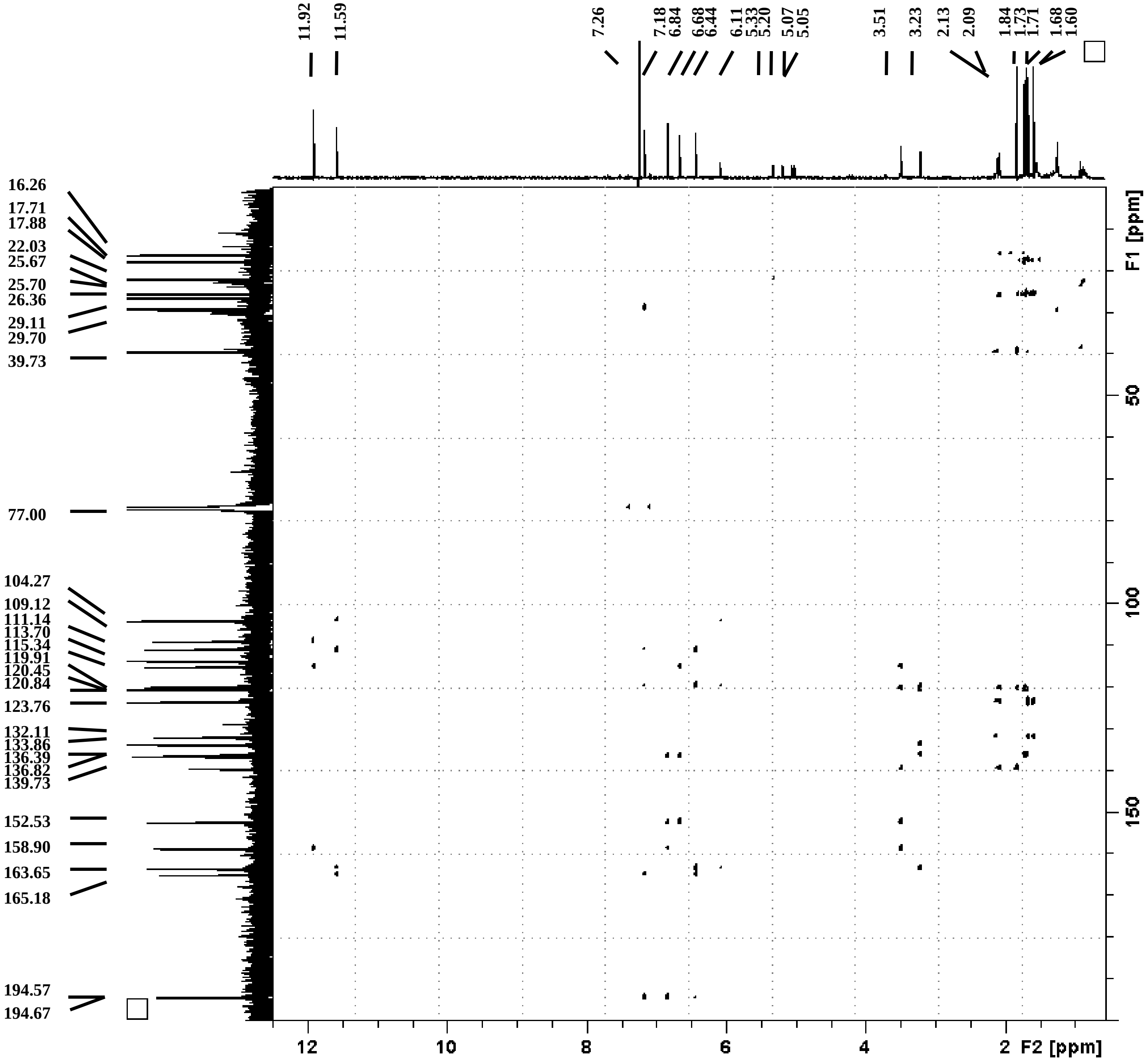

Figure S39. HMBC NMR spectrum of **11**.

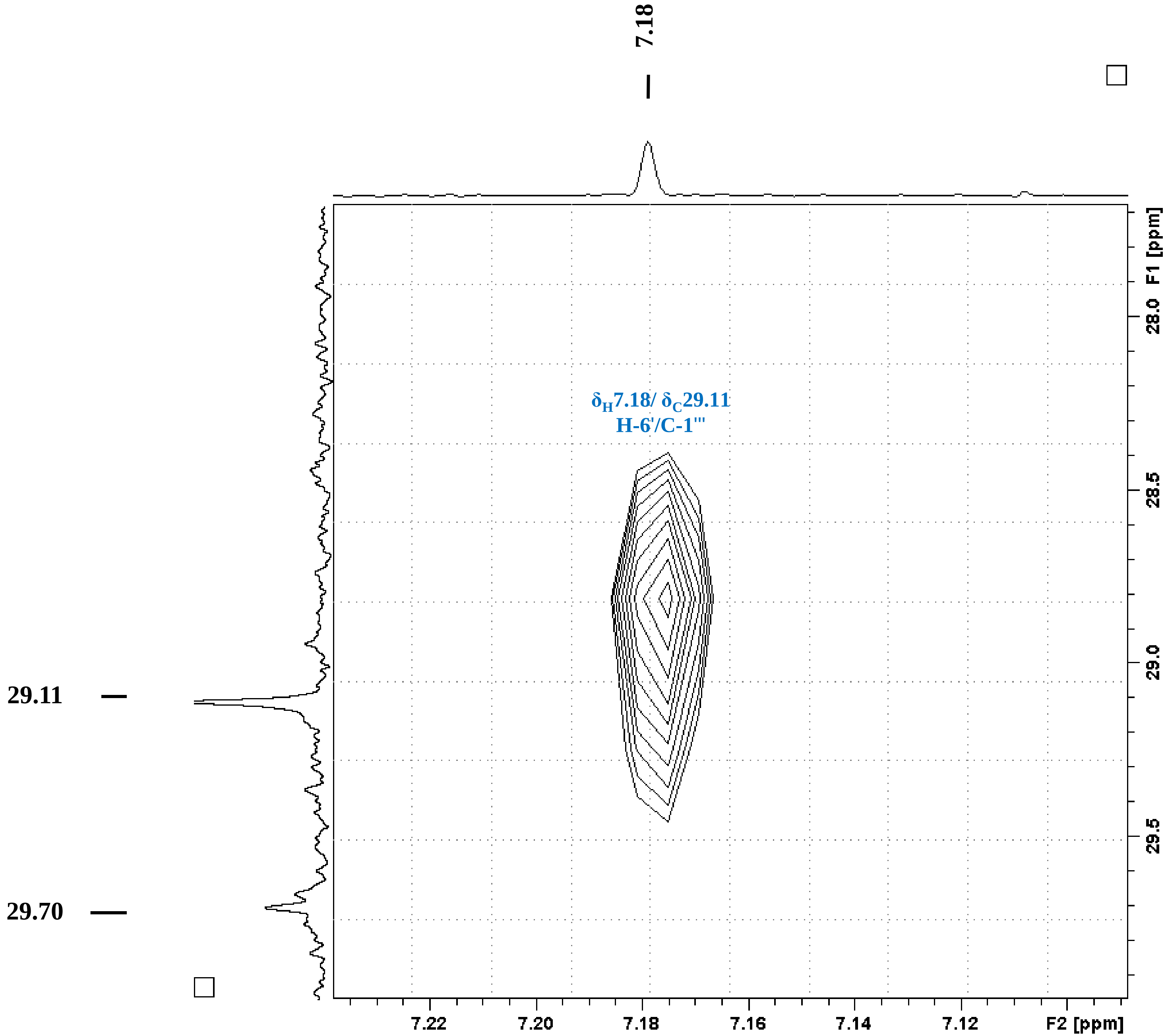

Figure S40. HMBC NMR spectrum of **11** (enlarged).

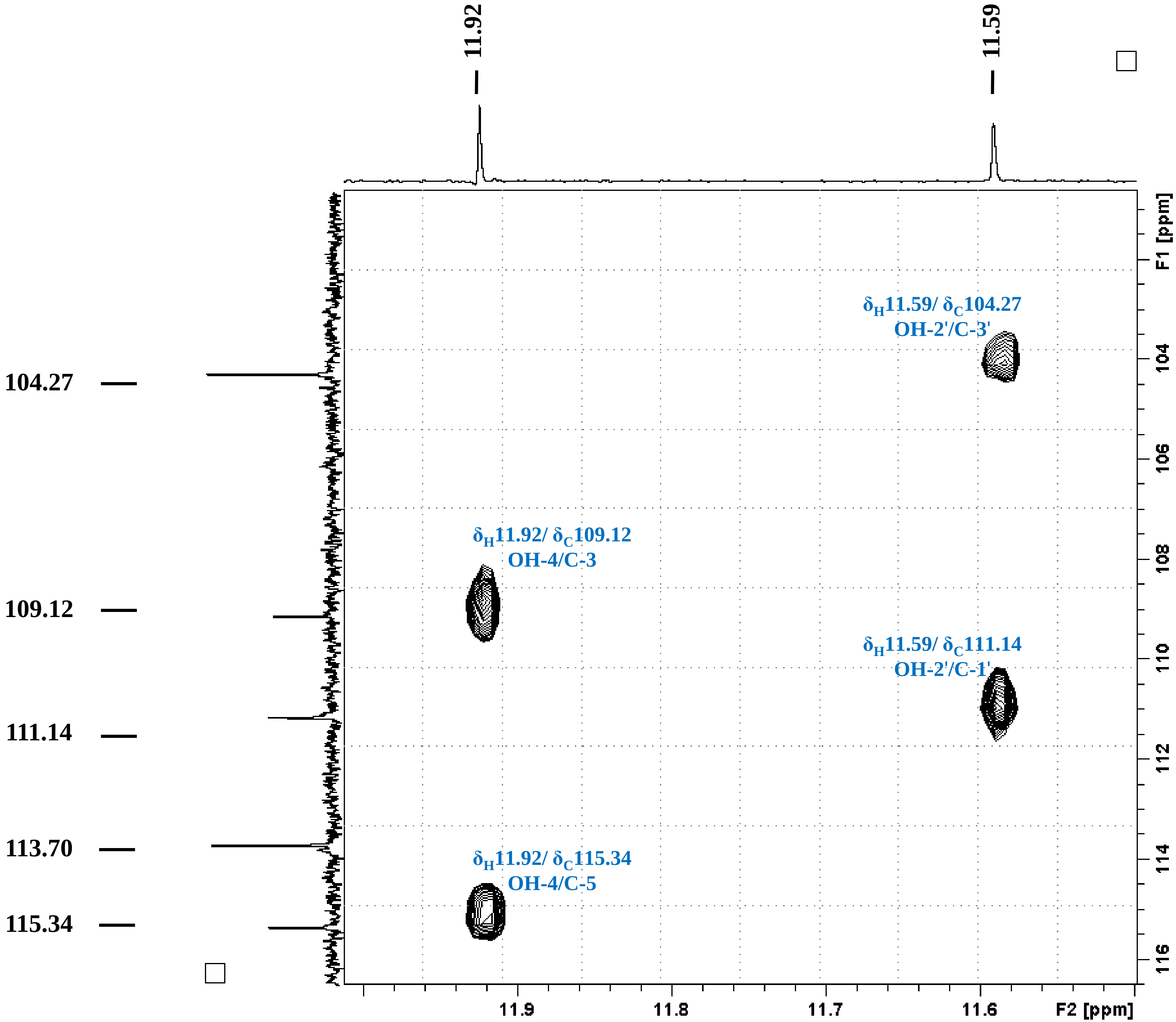

Figure S41. HMBC NMR spectrum of **11** (enlarged).

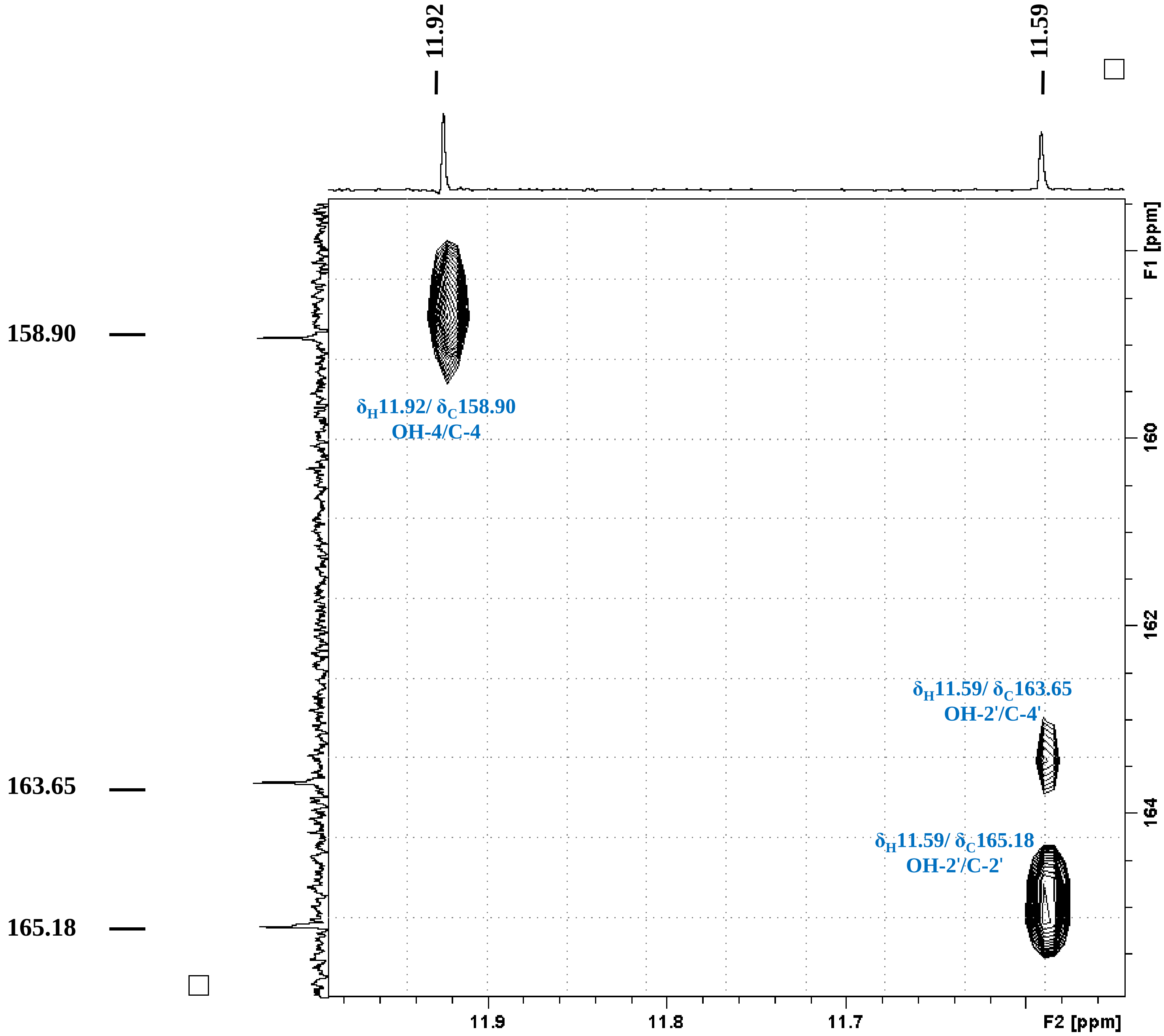

Figure S42. HMBC NMR spectrum of **11** (enlarged).

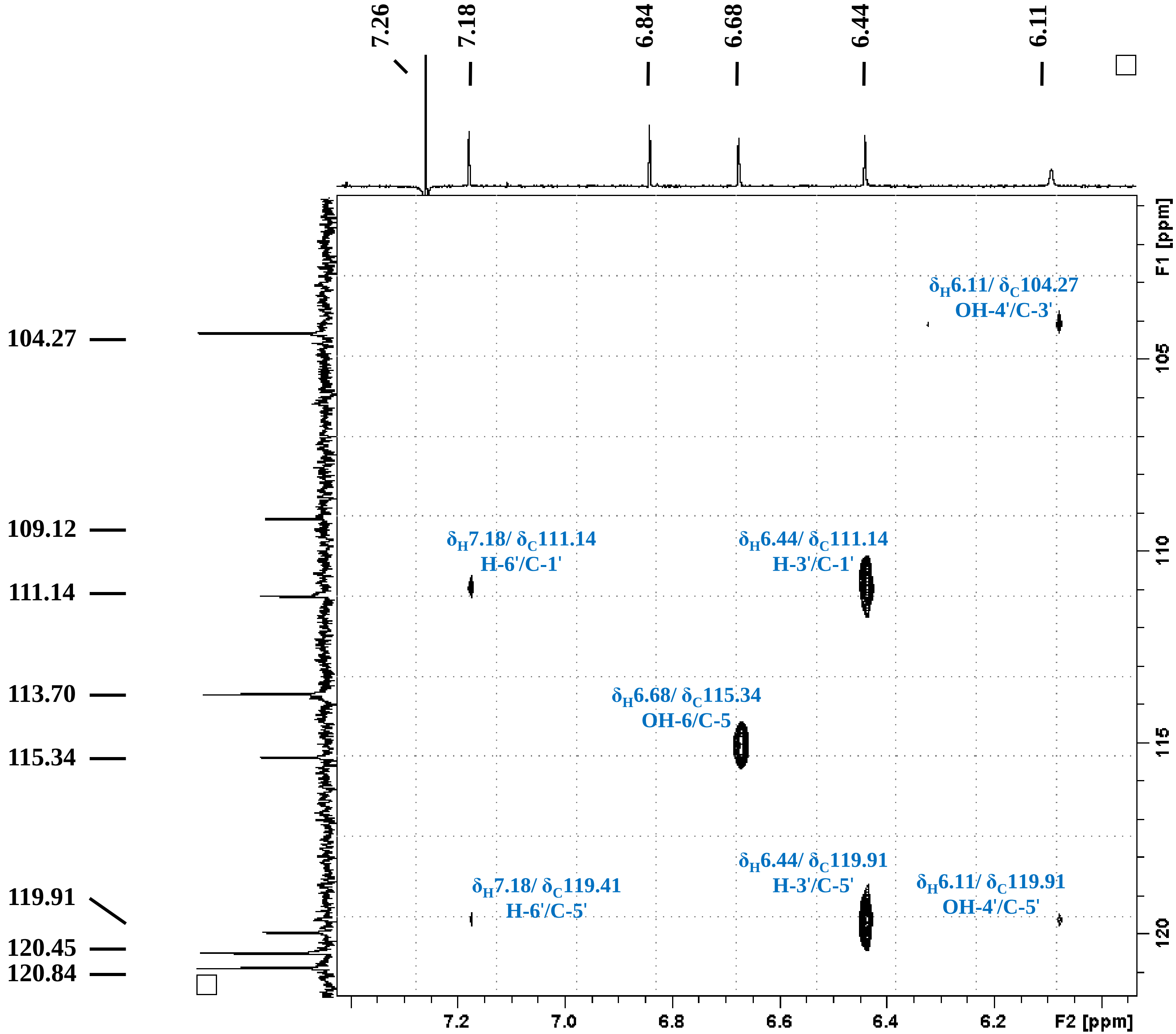

Figure S43. HMBC NMR spectrum of **11** (enlarged).

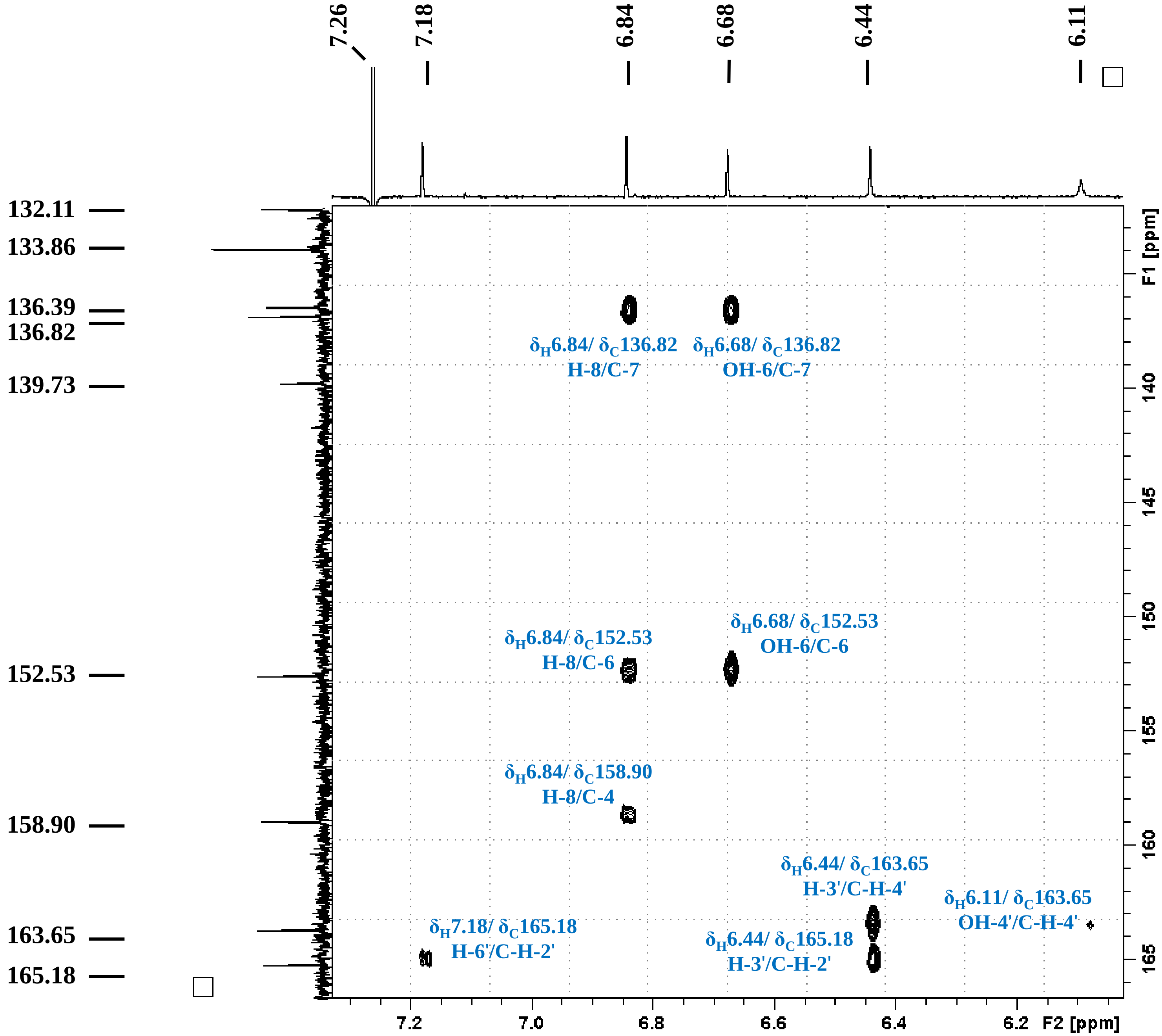

Figure S44. HMBC NMR spectrum of **11** (enlarged).

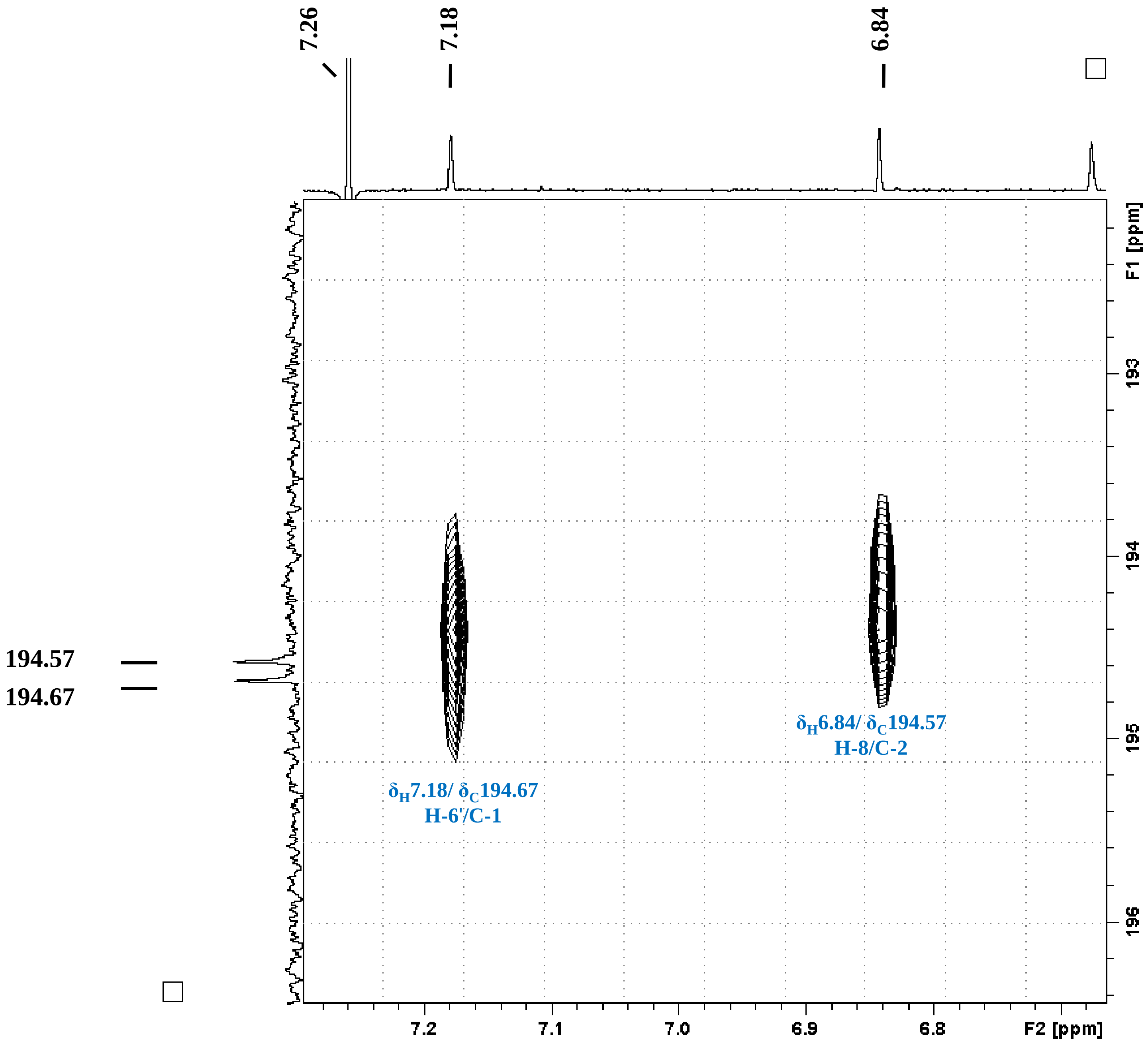

Figure S45. HMBC NMR spectrum of **11** (enlarged).

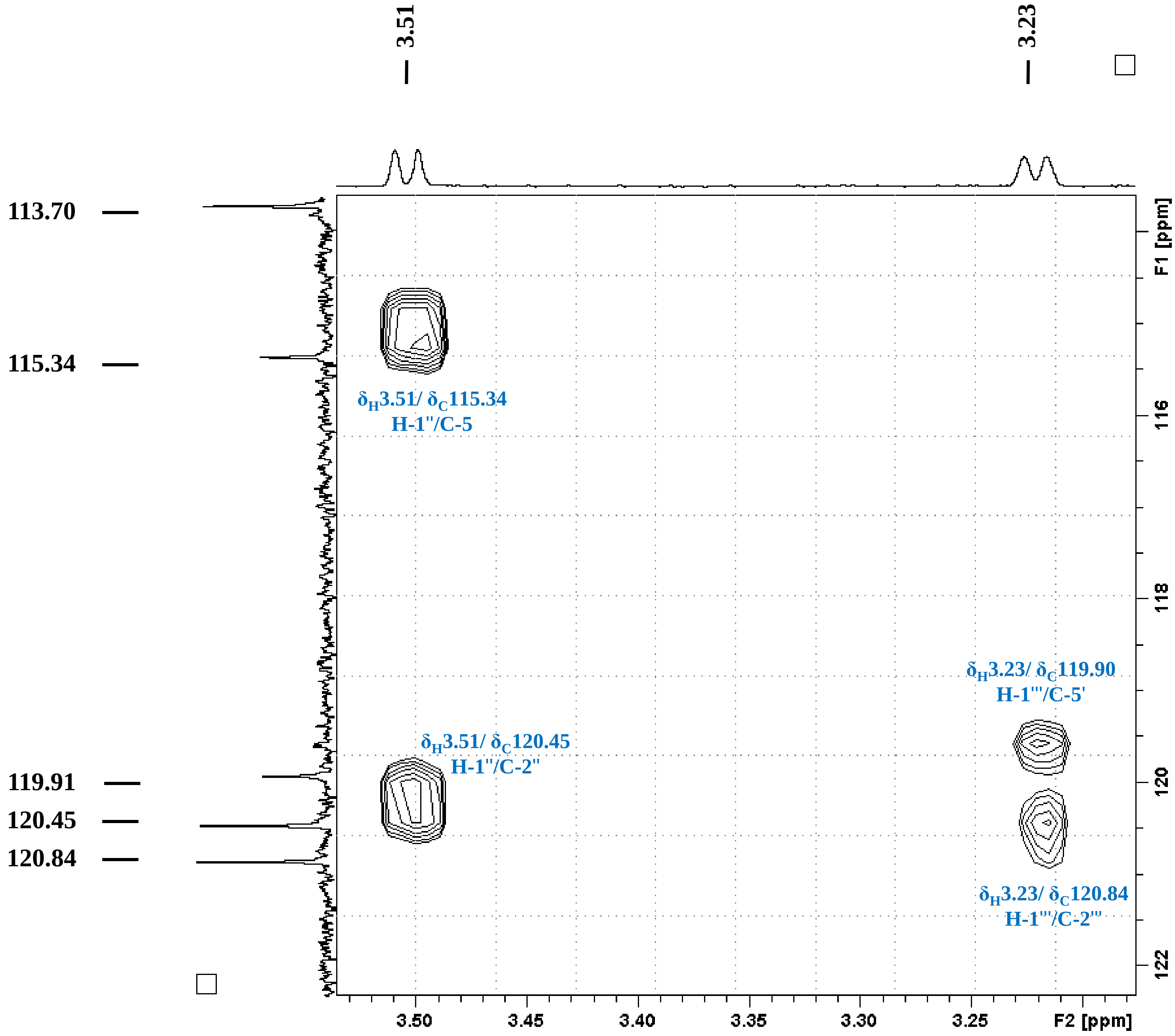

Figure S46. HMBC NMR spectrum of **11** (enlarged).

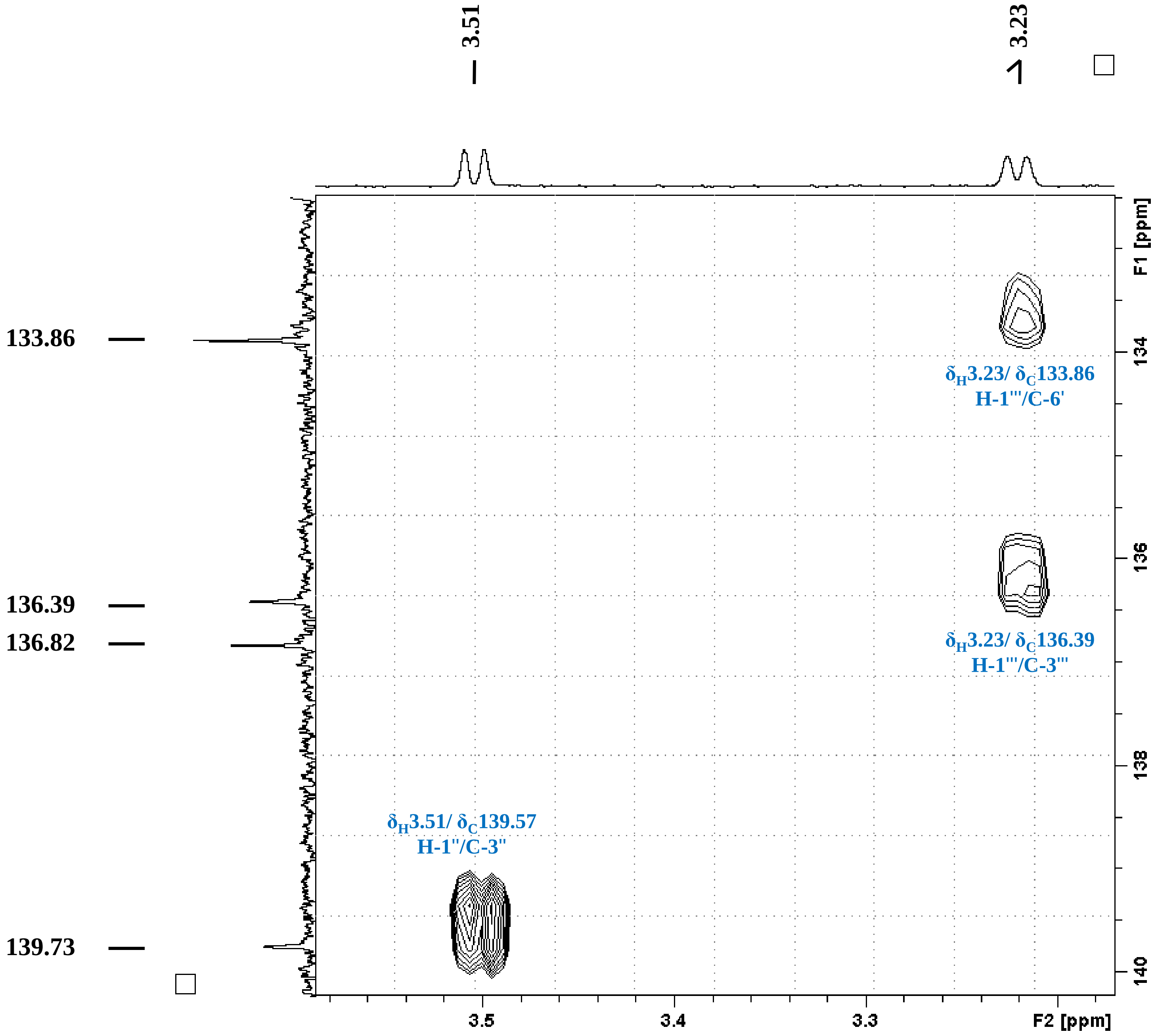

Figure S47. HMBC NMR spectrum of **11** (enlarged).

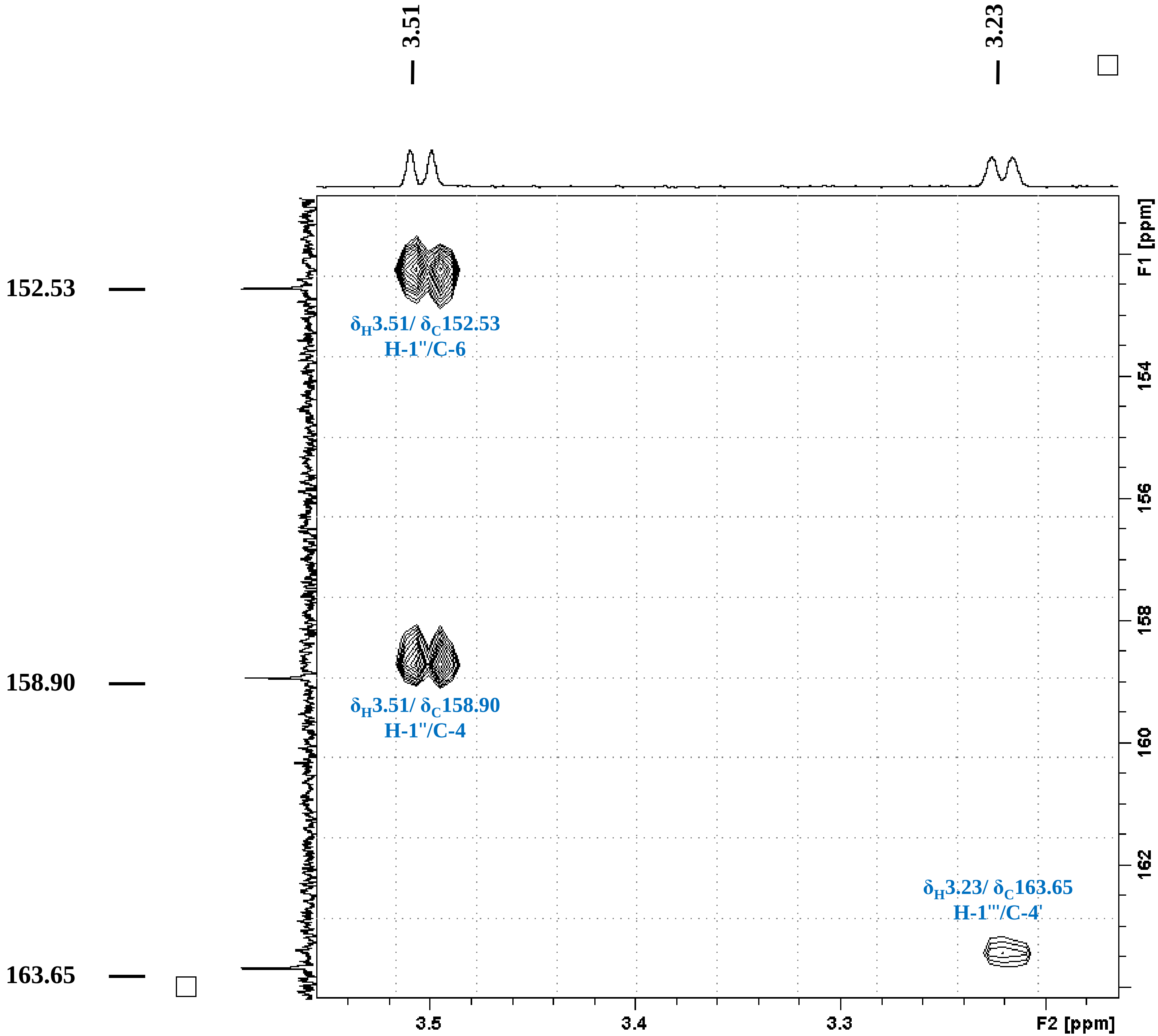

Figure S48. HMBC NMR spectrum of **11** (enlarged).

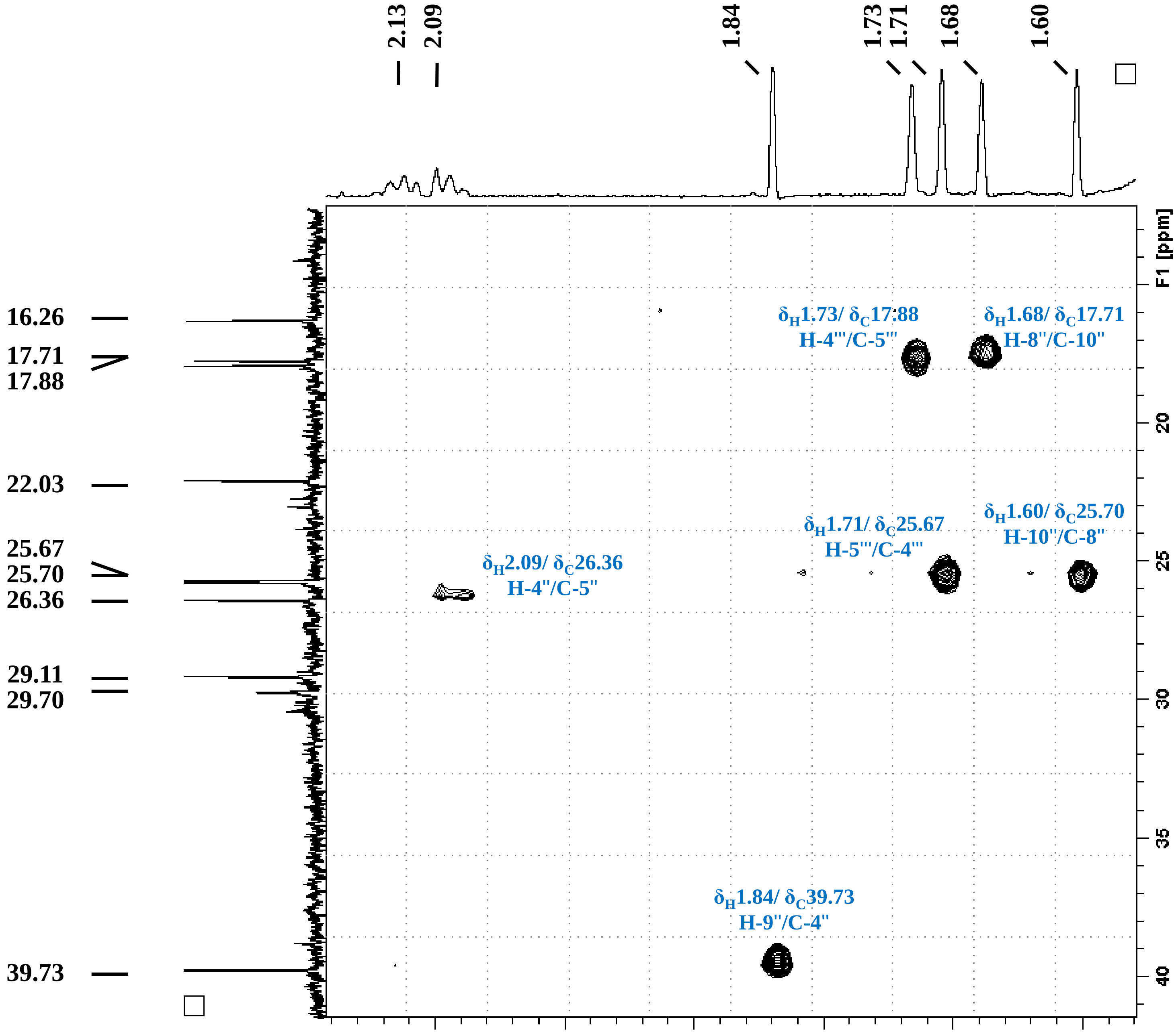

Figure S49. HMBC NMR spectrum of **11** (enlarged).

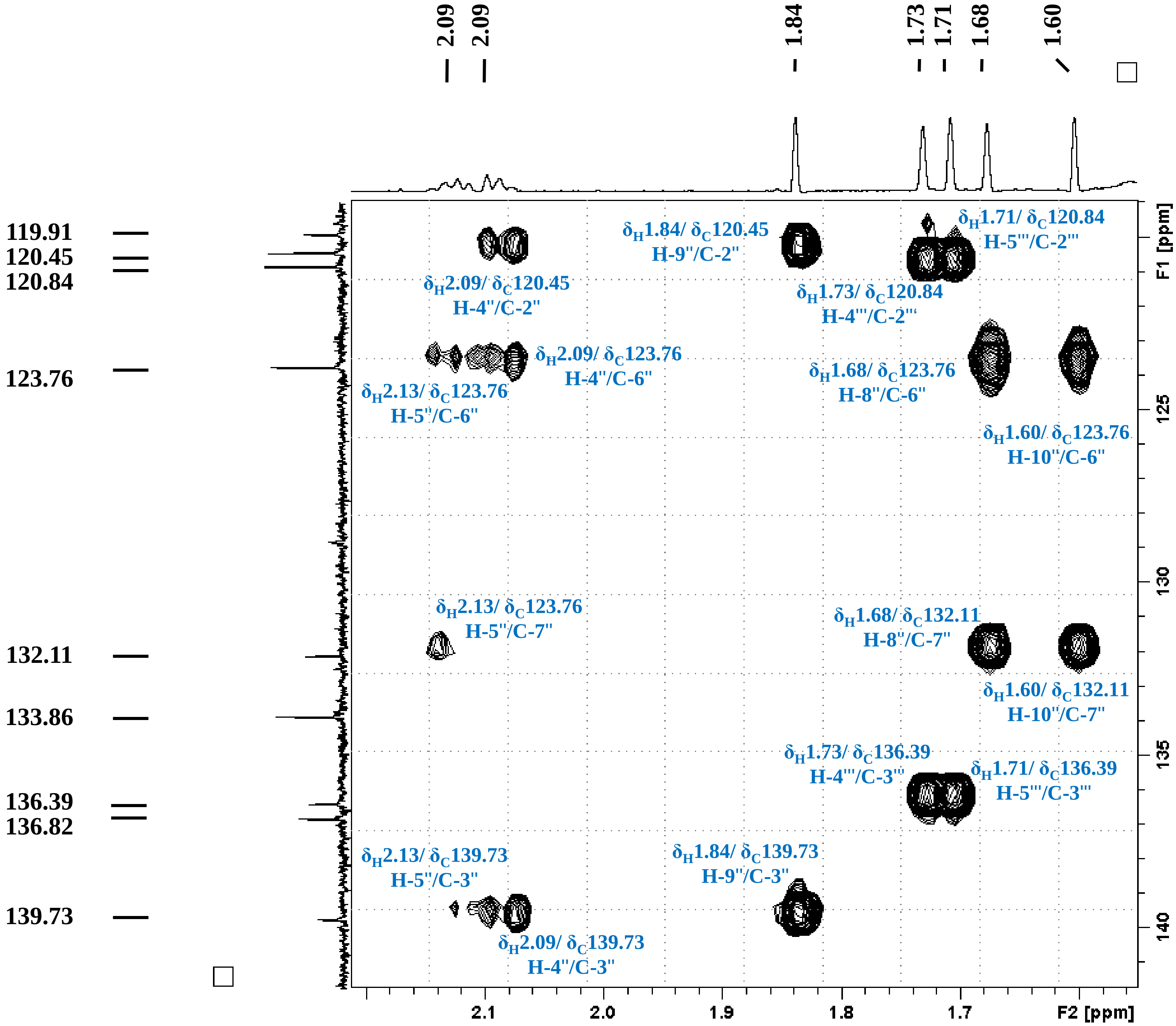

Figure S50. HMBC NMR spectrum of **11** (enlarged).

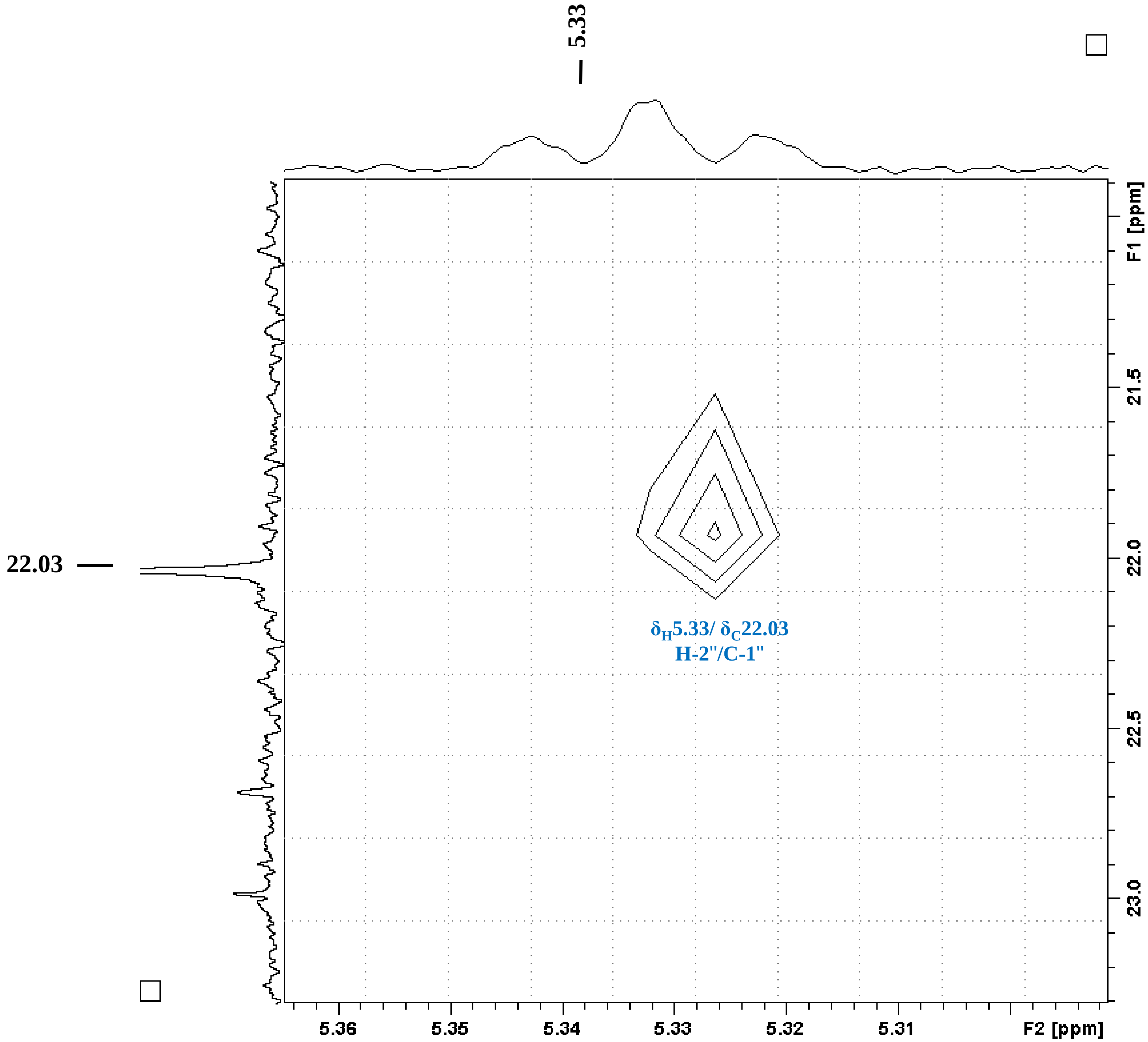

Figure S51. HMBC NMR spectrum of **11** (enlarged).

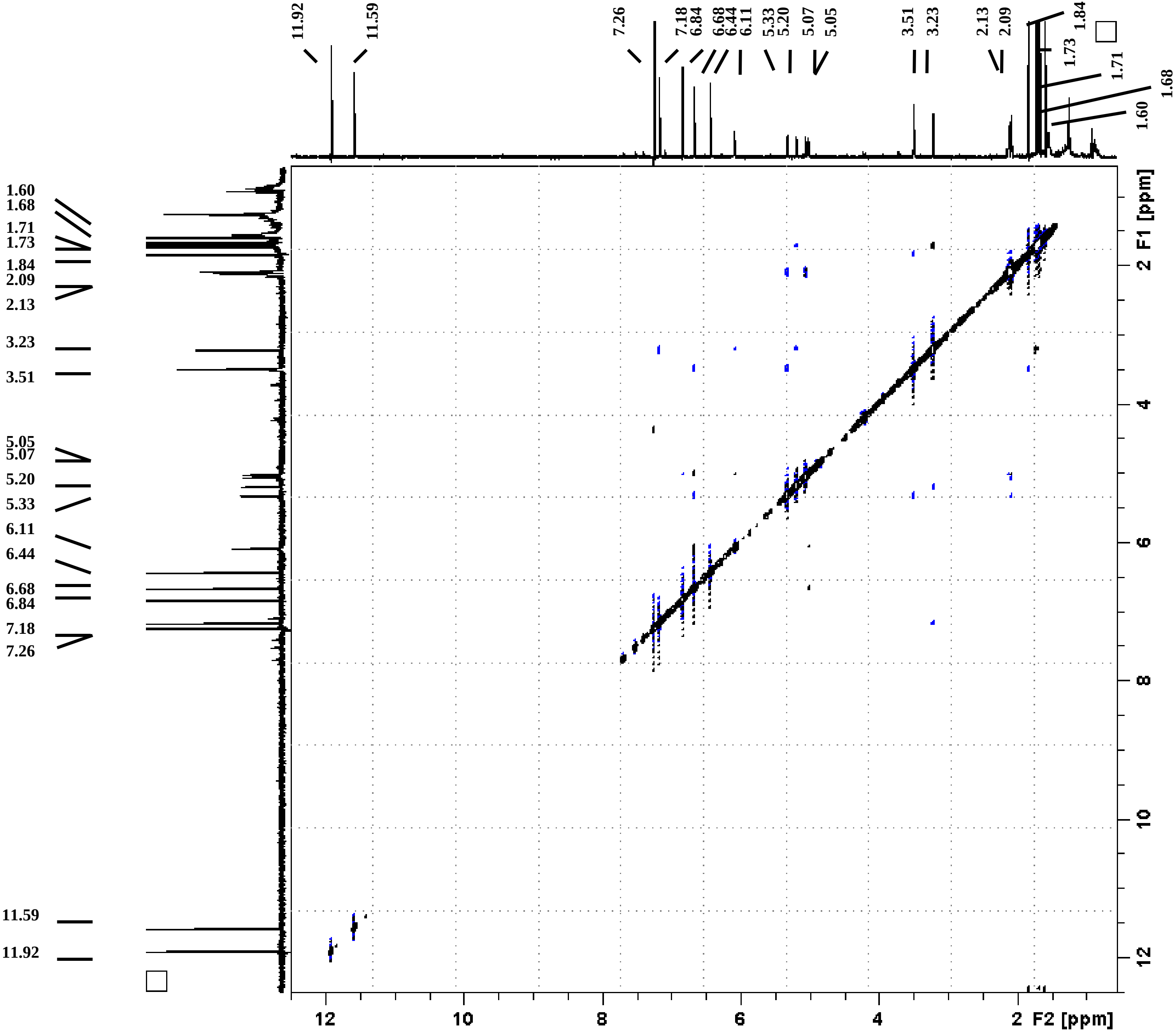

Figure S52. ROESY NMR spectrum of **11**.

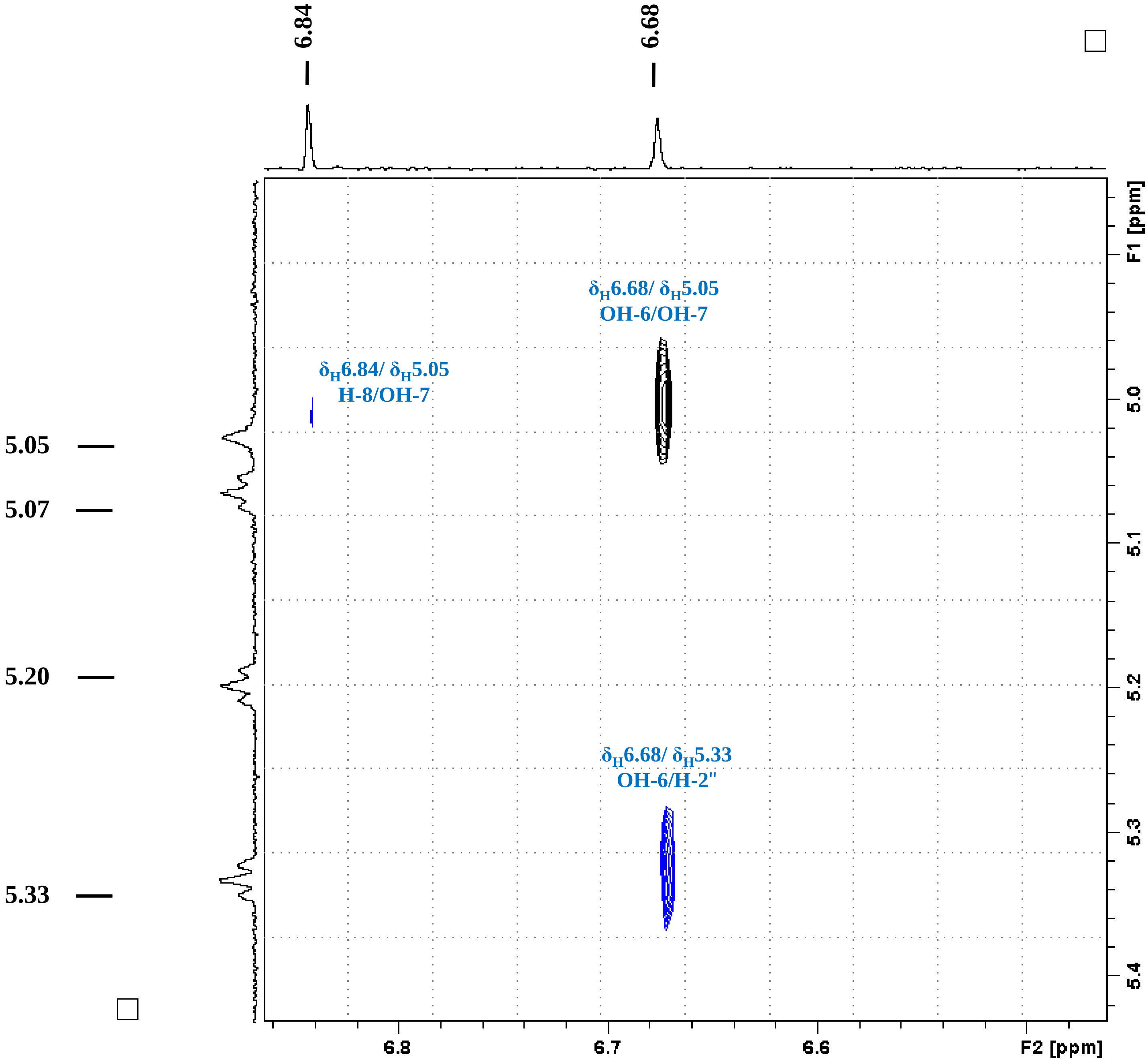

Figure S53. ROESY NMR spectrum of **11** (enlarged).

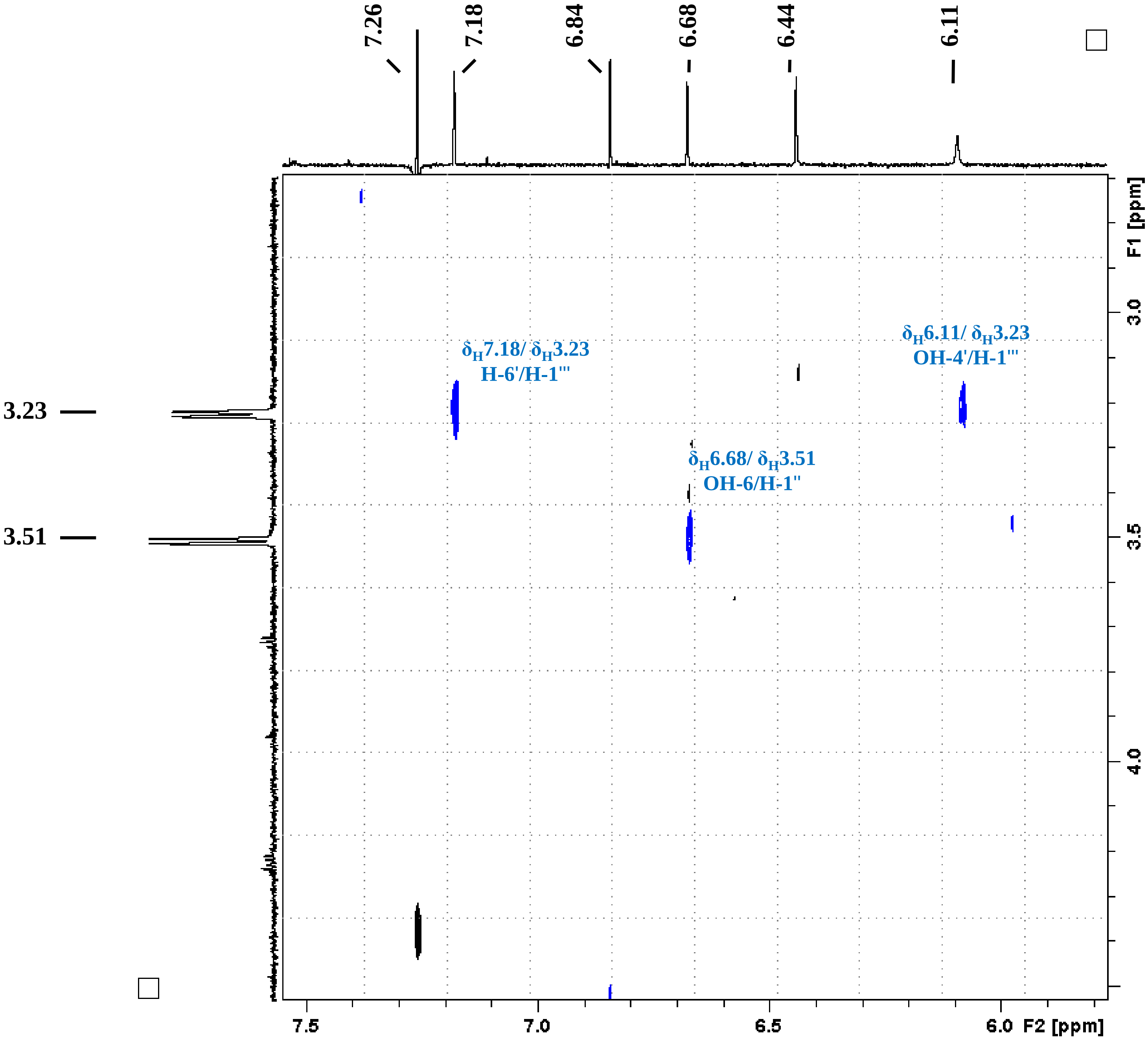

Figure S54. ROESY NMR spectrum of **11** (enlarged).

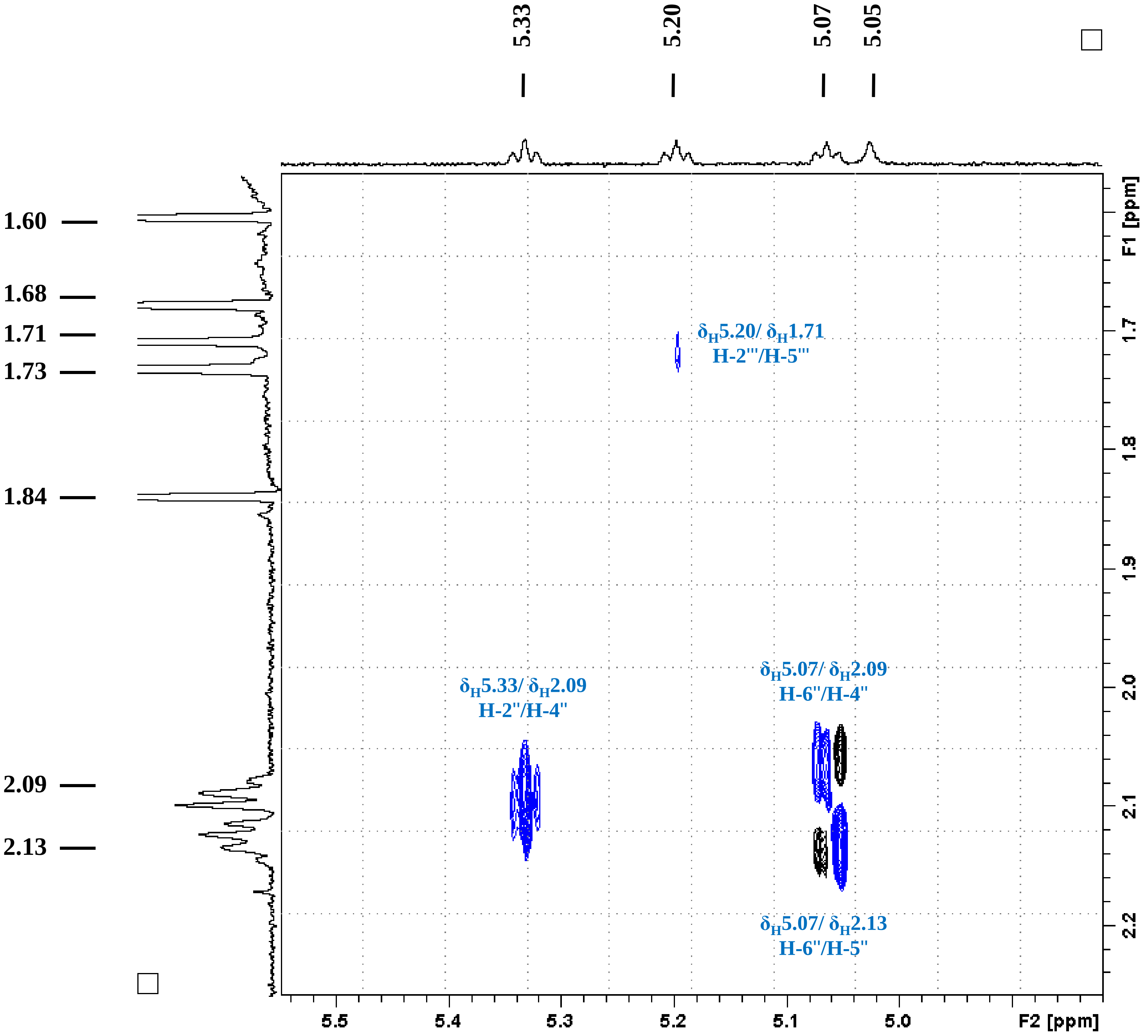

Figure S55. ROESY NMR spectrum of **11** (enlarged).

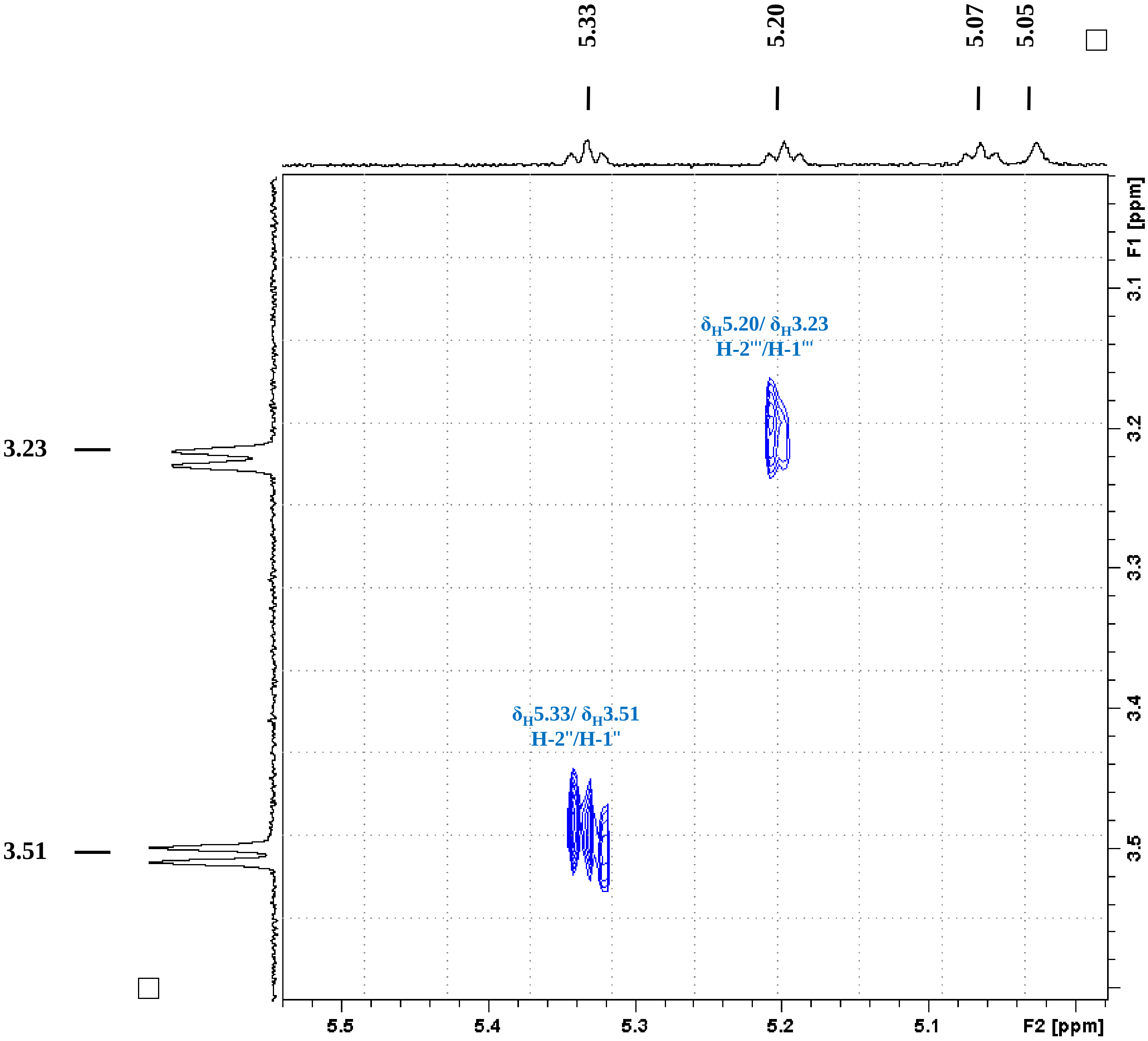

Figure S56. ROESY NMR spectrum of **11** (enlarged).

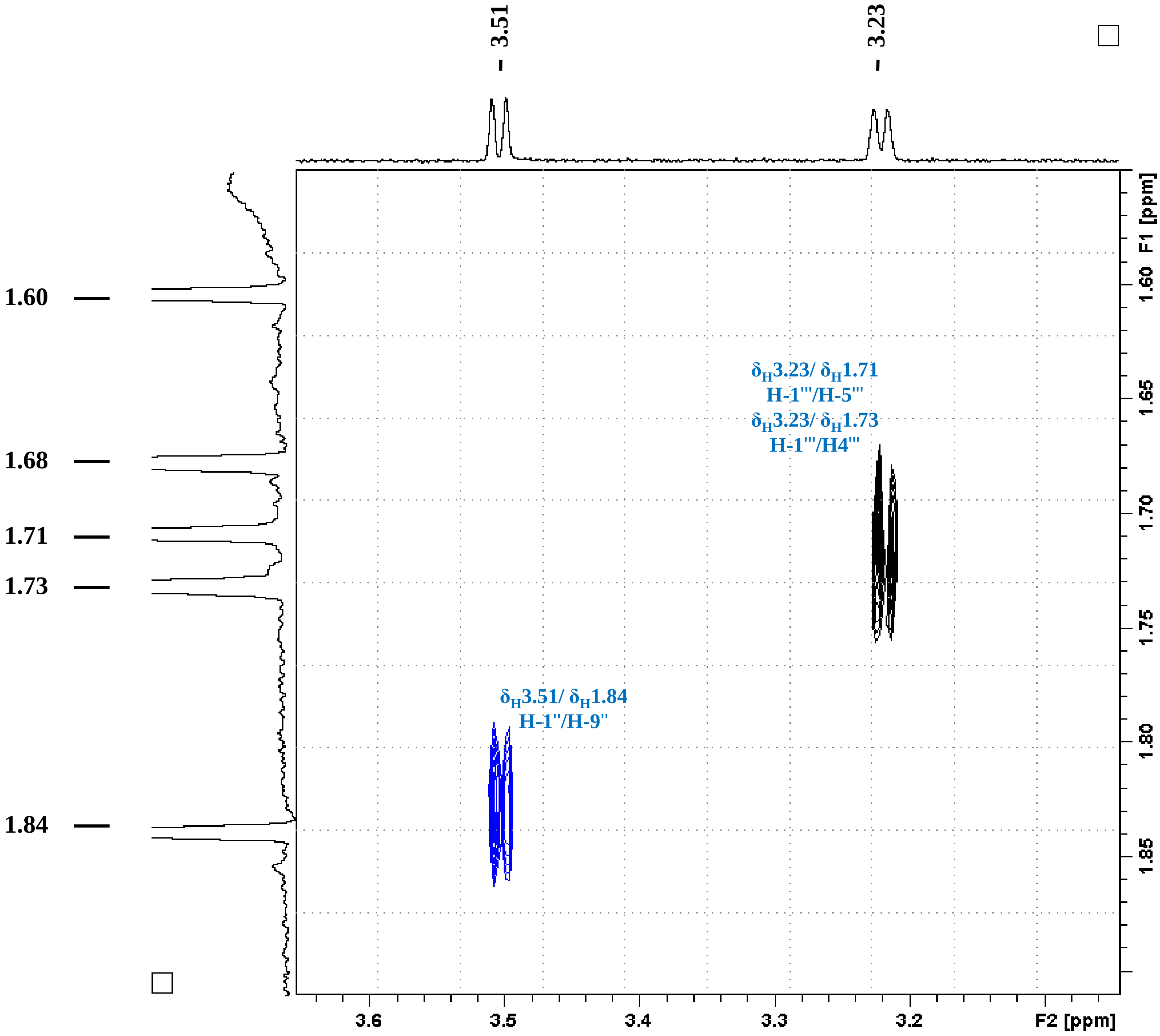

Figure S57. ROESY NMR spectrum of **11** (enlarged).

The specificity of compound **9** and **10** relative to compounds from our previous report [24] is that **9** and **10** have nearly flat geometry. The atoms of cycles A-D lie in one plain. But ECD spectra of **9** and **10** were similar to the spectrum of previously isolated 6a*R*,11a*R*,3'*S*-6a,11a-dihydrolespedezol A_3 (**2**) [24]. This let us to suppose, that the asymmetric centre in these molecules has the same nature – C-3'*S*.

The geometrical structure of compounds **9** and **10** was investigated using density functional theory (DFT). Special attention was devoted to their structural mobility. We investigated the inversion of cycle E and the internal rotation of the substituent at C-3'. Calculations showed that for **9** and **10**, dissolved in acetonitrile, the global minimums on the potential energy surface (PES) realize when dihedral angle $\theta_E \equiv \angle$C3'–O(ring E)–C2'–C10 ≈ -150°. The most stable conformations are characterized with “trans’ orientation of atom C-6' relative to atom C-3' – the dihedral angle $\theta_2 \equiv \angle$C3' –C4' –C5'–C6' ≈ 180° (the total amount of conformations, corresponding to this type, for each compound is nearly 95%). From them the more stable are conformations with “trans’ orientation of atom C-5' relative to atom C-9' – the dihedral angle $\theta_1 \equiv \angle$C5'–C4'–C3'–C9' ≈ 180°. Ring E is very flexible, its inversion proceeds with the overpassing a low potential barrier ($\Delta E^{\#} \approx 3$ kcal · mol^{-1}). But when **9** and **10** exist in conformation, for which $\theta_1 \approx 180°$ and $\theta_2 \approx 180°$, due to the steric factors the inversions of ring E can not proceed completely – the potential energy surfaces did not contain minimums, corresponding to conformations, characterized by dihedral angle $\theta_E \approx +150°$ (Supplementary data, S59). Nevertheless, the scanning of PES along dihedral angle θ_E showed, that the area near global minimum on PES (denoted as “**RE**”) is very flat – thus, the change of θ_E on the value $\Delta\theta_E = \theta_E - \theta_{E,re} = +30°$ leads to a change in electronic energy $\Delta E_{el} \approx 1$ kcal · mol^{-1}. This means that at room temperatures the conformations, corresponding to these values of dihedral angle θ_E, will be observed in spectroscopic experiments. This fact was accounted for during theoretical simulations of ECD spectrum of compounds **9** and **10**.

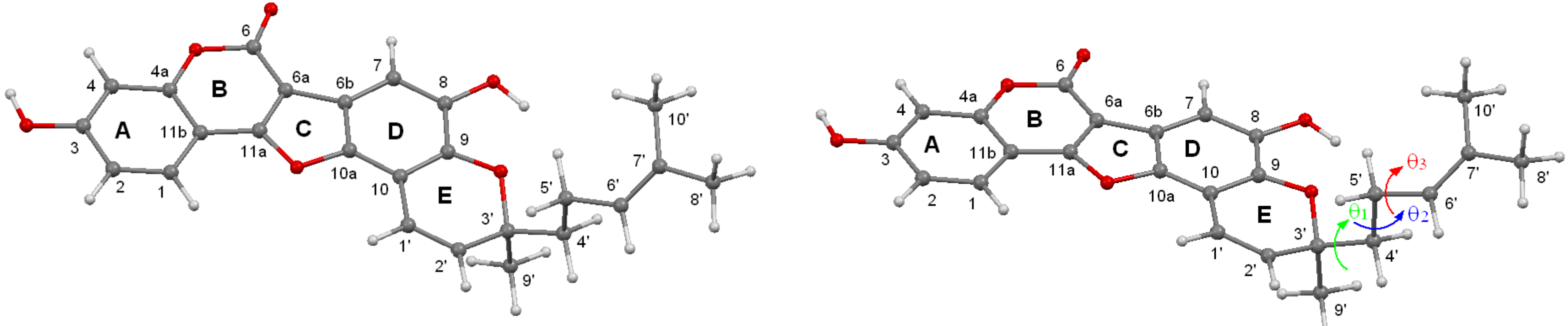

Figure S58. The geometrical structure and atom numeration for compounds **9** and **10**.

Different conformations were denoted according to abbreviation:

Conformation = B^iE^j–αβγ , where indexes “*i*” and “*j*” point the conformations of rings B and E, according to “+” or “–” signs of dihedral angles θ_B and θ_E, respectively♣:

i = “–” when $\theta_B \equiv \angle$C4a–C11b–C11a–C6a ≈ -10°; i = “+” when θ_B ≈ + 10°;

j = “–” when $\theta_E \equiv \angle$C3' –O(ring E)–C2' –C10 ≈ - 150°; j = “+” when θ_E ≈ + 150°;

indexes “α”, “β” and “γ” point the rotameric forms of substituent at C-3', which are determined according to the values of dihedral angles θ_1, θ_2 and θ_3, respectively:

$\theta_1 \equiv \angle$C5' –C4' –C3' –C9'; $\theta_2 \equiv \angle$C6'–C5'–C4'–C3'; $\theta_3 \equiv \angle$C7'–C6'–C5'–C4';

a: $0° \le \theta \le 140°$
b: $-140° \le \theta \le 0°$
t: $140° \le \theta \le 220°$

♣ the angle’s values correspond to minimums on PES.

Scheme S59. Abbreviations for conformations of compounds **9** and **10**.

The rotation of substituent at C-3', as well as the rotations of it's fragments around corresponding C-C bonds, - all these large-amplitude motions (LAM) influence the shape of ECD spectrum. When compound **9** is under study, one more LAM had to be accounted for – the inversion of ring B. This LAM causes the swing of Plain 1, in which lie atoms C11a, O (ring B) and atoms of ring A, relative to Plain 2, in which lie atoms C6, O (at C6) and atoms of cycles C and D (C11a – C11b bond is an axis of rotation). The swing of Plain 1 and Plain 2 relative to each other causes several subsequences. They may be clarified with the usage of Scheme S60.

During the inversion rearrangement the θ_B angle changes from ≈ +9.5° to ≈ -9.5° (and vice versa). As a result, in many cases the atoms of cycles B, C and D, which before inversion "conventionally stay upper Plain 1" (the "+" area, Scheme S60), after inversion "conventionally stay under Plain 1" (the "-" area, Scheme S60). This results in changes in the rotatory strengths of many electronic transitions, and what is especially famous – for these transitions not only the absolute value, but also the sign of the rotatory strengths change. As a result the statistical averaging of ECD spectra for conformations, differing only in the "state of inversion" of cycle B (as are, for example, conformations **B^-E^-_bta** and **B^+E^-_bta**, Scheme S60) may result in that for several energy diapasons (wave-lengths regions) the bands in averaged ECD spectrum may be for an order less intensive than they are for the one chosen conformation.

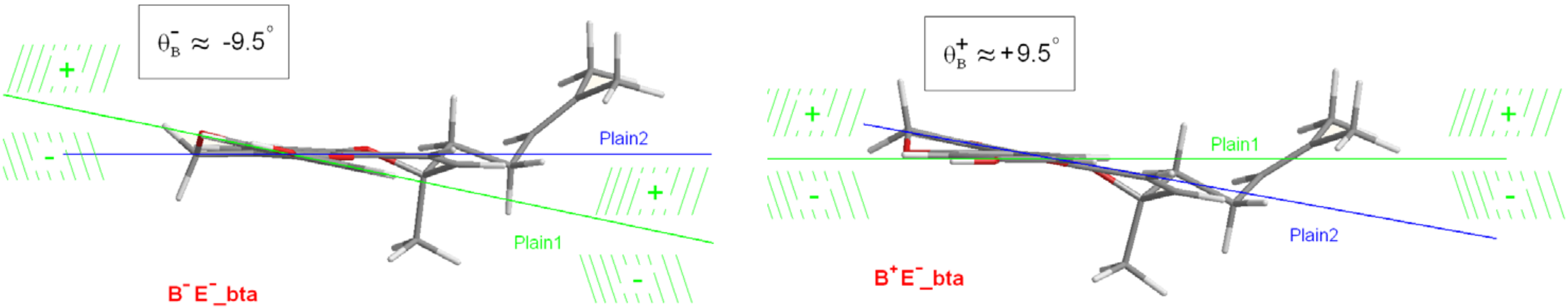

Scheme S60. The influence of inversion of ring B on geometrical structure of compound **9**.

The shapes of calculated statistically averaged ECD spectra are influenced by the relative amounts of the E^- and E^+ conformations. We performed a number of simulations of the averaged ECD spectrum for **9**, in which amounts of the E^- and E^+ conformations (denoted as $a(\zeta)$ and $b(\zeta)$, respectively) were varied manually according to the equations:

1. *The data, obtained with B3LYP/6−311G(d)_PCM method*

$$a_0 = \sum_{i=1}^{N1} g_{i,calc}(E^-); \quad b_0 = \sum_{j=1}^{N2} g_{j,calc}(E^+);$$

$$a_0 + b_0 = 1;$$

2. *We used one varional parameter* ζ:

$$a(\zeta) = \zeta \cdot a_0; \quad b(\zeta) = 1 - a(\zeta);$$

3. *Recalculated statistical weights of individual conformations are*:

$$g_{i,\zeta}(E^-) = \zeta \cdot g_{i,calc}(E^-);$$

$$g_{j,\zeta}(E^+) = \frac{1 - \zeta \cdot a_0}{b_0} \cdot g_{j,calc}(E^+);$$

We found that the shapes, positions and relative intensities of individual bands in characteristic region $200 \leq \lambda \leq 300$ nm are stable to these variations, while they change pronouncedly in $\lambda \geq 300$ nm region (Fig. S62). The analogous behavior we found earlier for 6a*R*,11a*R*,3'*S*-6a,11a-dihydrolespedezol A_3 (**2**) [24].

Scheme S61. Equation for recalculation of the relative contributions of E^- and E^+ conformations to ECD spectra of compounds **9** and **10**.

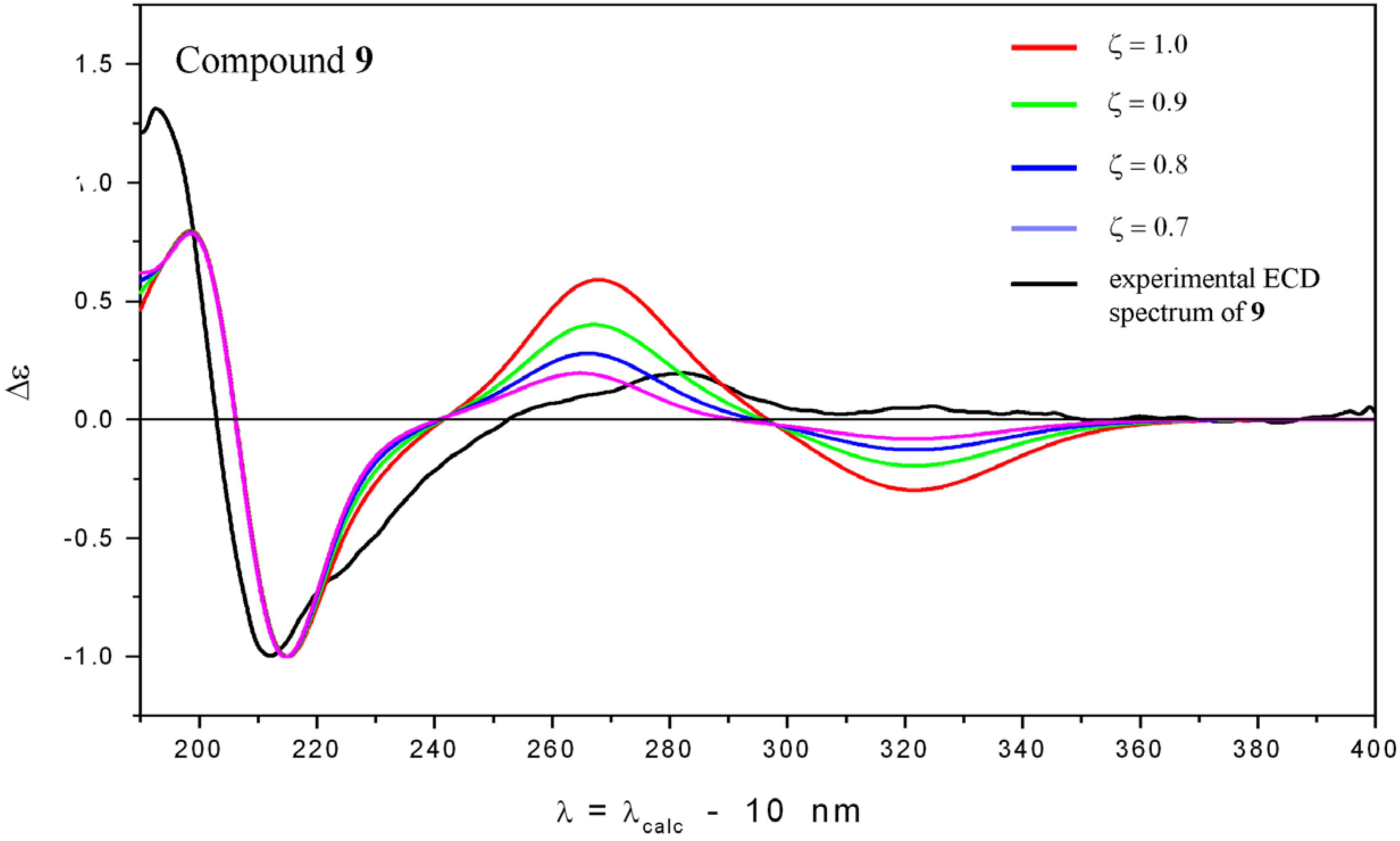

Figure S62. The influence of variation of relative amounts of E^{-} and E^{+} conformations on the shape of statistically averaged scaled ECD spectrum of compound **9.** The scaled theoretical and experimental ECD spectra were obtained according to equation:

$$\Delta\varepsilon_{scaled}(\lambda) = \Delta\varepsilon(\lambda) \Big/ \left|\Delta\varepsilon(\lambda_{peak})\right|$$

where the denominator $|\Delta\varepsilon(\lambda_{peak})|$ is a modulo of the peak value for the characteristic band at $\lambda \approx 216$ nm in the corresponding ECD spectrum.
